# Meeting materials from the 3rd Annual Meeting of the *International Society for the Prevention of Tobacco Induced Diseases*

**DOI:** 10.1186/1617-9625-2-4-168

**Published:** 2004-12-15

The meeting materials from the 3rd Annual Meeting of the *International Society for the Prevention of Tobacco Induced Diseases* are available as a zip drive in additional file 1

## Supplementary Material

Additional file 1 Click here for file

