# Supplementary material for: Meeting materials from the 3rd Annual Meeting of the International Society for the Prevention of Tobacco Induced Diseases
Source: Tob Induc Dis. 2004 Dec 15;2(4):168. doi: 10.1186/1617-9625-2-4-168 (PMC2671527; doi:10.1186/1617-9625-2-4-168)
Supplement: Additional file 1 [file 1617-9625-2-4-168-S1.zip › Abstract 25-Two week nicotine treatment selectively increases bone vascular constriction in.pdf]

Abstract 25

Two week nicotine treatment selectively increases bone vascular constriction in response to norepinephrine

Fleming J.T., J.B.A. Feitelson, P.P. Rowell, C.S. Roberts
Departments of Physiology and Biophysics and Pharmacology and Toxicology, University of Louisville, Louisville, Kentucky, USA

Smoking has been associated with delayed bone fracture healing and non-union in humans and animal models. Since an adequate blood supply is essential for normal osseous union, we hypothesized that the delayed union among smokers is due to a vasoconstrictor effect of nicotine on the blood vessels which regulate blood flow to bones. Nicotine (1.7 mg/kg/day for 2 weeks) was delivered to rats via osmotic mini-pumps. Control rats were treated with nicotine-free physiologic solution. On day 14, *in vivo* experiments were performed to assess the constrictor function of the vascular smooth muscle (constriction to norepinephrine and endothelin), and the dilator function of the vascular endothelium (dilation in response to acetylcholine). The results revealed that nicotine significantly enhanced bone vascular constriction in response to norepinephrine but not to endothelin. Endothelial cell dilator function was not altered. Thus, nicotine selectively enhances bone vascular constriction to norepinephrine. The augmented constriction to norepinephrine alone was not due to a decrease in the dilator function of the endothelium. An increase in bone vascular constriction to norepinephrine would be expected to reduce blood flow to bone and could explain, in part, the delay in bone fracture healing among smokers.
