# Supplementary material for: Meeting materials from the 3rd Annual Meeting of the International Society for the Prevention of Tobacco Induced Diseases
Source: Tob Induc Dis. 2004 Dec 15;2(4):168. doi: 10.1186/1617-9625-2-4-168 (PMC2671527; doi:10.1186/1617-9625-2-4-168)
Supplement: Additional file 1 [file 1617-9625-2-4-168-S1.zip › Abstract 26-Evaluation of a statewide population-based cessation contest.pdf]

Abstract 26

Evaluation of a statewide population-based cessation contest

Ellen J. Hahn, DNS, RN; Mary Kay Rayens, PhD; Dawn M. Christie, MA;
Lisa W. Greathouse, MSN, RN; Nancy L. York, MSN, RN
College of Nursing • University of Kentucky • Lexington, Kentucky

The purpose of this study was to evaluate the impact of a Quit and Win Contest on tobacco quit rates at 3, 6, and 12 months after a 30-day quit period.

A volunteer sample of 494 Quit and Win Contest registrants comprised the Treatment Group and a random sample of 512 tobacco users not exposed to the promotional media campaign formed the Control Group. The intervention included a 30-day quit period to be eligible for large cash prizes; and provided gender-specific cessation advice via weekly mailings; on-line and telephone quit assistance; media campaign; and community support. Quit rates were measured using 7-day point prevalence for tobacco use. Urine cotinine measurements confirmed self-reported quitting at all follow-up interviews.

Treatment group participants were significantly more likely than Controls to experience quitting during the one-year follow-up, as determined by both self-report and urine confirmation. After adjusting for baseline differences in demographics, tobacco use, and Stage of Change, those in the Treatment group were 2.6 times more likely than Controls to report quitting in the post intervention period, and 5.3 times more likely to experience quitting confirmed by urine cotinine. Women, minorities, and low-income tobacco users were just as likely to quit as men, Caucasians, and those with higher incomes.

The fact that the contest was minimally intensive and yielded a relatively high quit rate demonstrates the potential effectiveness of the intervention.
