# Supplementary material for: Meeting materials from the 3rd Annual Meeting of the International Society for the Prevention of Tobacco Induced Diseases
Source: Tob Induc Dis. 2004 Dec 15;2(4):168. doi: 10.1186/1617-9625-2-4-168 (PMC2671527; doi:10.1186/1617-9625-2-4-168)
Supplement: Additional file 1 [file 1617-9625-2-4-168-S1.zip › Abstract 27-Reducing Secondhand Tobacco Smoke.pdf]

Abstract 27

Reducing Secondhand Tobacco Smoke: Cardiac and Asthma Outcomes

Ellen J. Hahn, DNS, RN; Debra K. Moser, DNSc, RN, FAAN;
Patricia Burkhart, PhD, RN; Mary Kay Rayens, PhD; Nancy L. York, MSN, RN
College of Nursing • University of Kentucky • Lexington, Kentucky

The specific aim of this study is to evaluate the effects of a community initiative to reduce exposure to secondhand tobacco smoke (SHTS) on cardiac and asthma outcomes, including the rate of hospital and emergency department (ED) discharges, length of stay, and total hospital costs. The broad, long-term objective is to assess the relationship between reduction in exposure to SHTS at the community level and health care outcomes associated with acute coronary syndromes and asthma.

Although many local communities have adopted measures to reduce exposure to SHTS, the tobacco-growing states lag behind in protecting workers from SHTS. The Lexington-Fayette (Kentucky) Urban County Government enacted a smoke-free law on July 1, 2003, making all public buildings smoke-free including, but not limited to, restaurants, bars, bowling alleys, bingo halls, laundromats, and racetracks. Enforcement of the initiative began April 27, 2004 after a favorable Kentucky Supreme Court ruling. Lexington's initiative is considered by the Americans for Nonsmoker's Rights as one of the strictest local laws in the U.S.

The study is a time series design using five 6-month time periods; three time points prior to and two time points after the April 27th enforcement of the Smoke-Free Lexington initiative. The administrative data base (Comp Data) used to summarize and track all Kentucky hospital billing records will be accessed for all Lexington hospitals during each of the five time periods. Comp Data will be queried for hospital discharge diagnosis by DRGs and ICD-9 codes, length of stay, total hospital charges, dates of service, gender, age, and zip code of residence. Since Comp Data does not compile ED discharge information, each Fayette County hospital will provide all data fields of interest above for the ED discharge diagnosis by DRGs and ICD-9 codes. Multiple linear regression will be used to determine predictors for length of stay and total costs; predictors will include year, month, gender, age, and an indicator variable for whether the observation occurred before or after the initiative. For the number of events (hospital and ED discharges) per at-risk population, an incidence rate, Poisson regression will be used with the same predictors.
