# Supplementary material for: Meeting materials from the 3rd Annual Meeting of the International Society for the Prevention of Tobacco Induced Diseases
Source: Tob Induc Dis. 2004 Dec 15;2(4):168. doi: 10.1186/1617-9625-2-4-168 (PMC2671527; doi:10.1186/1617-9625-2-4-168)
Supplement: Additional file 1 [file 1617-9625-2-4-168-S1.zip › Abstract 28-Underestimation of mortality due to chronic obstructive pulmonary disease (COPD).pdf]

Abstract 28

Underestimation of mortality due to chronic obstructive pulmonary disease (COPD) in Kentucky

Therese S. Hughes, MPA, Susan B Muldoon, PhD, MPH
University of Louisville School of Public Health and Information Sciences
Department of Epidemiology and Clinical Investigation Sciences

COPD mortality may be underestimated because COPD is frequently listed as a contributory cause of death, and not as the underlying cause of death on death certificates. One of the limitations of state mortality statistics is that many decedents with COPD have their deaths attributed to other causes due to co-morbidity. For example, COPD and heart disease are often co-morbid conditions, and it is not uncommon for COPD patients to die from heart failure in end-stage disease. On death certificates, these deaths are often attributed to heart disease, however the more accurate cause of death would be COPD. This study's objective was to evaluate the underestimation of COPD as cause of death per Kentucky (KY) mortality records by determining the frequency with which COPD was listed as a contributory cause of death, rather than an underlying cause of death per state mortality records for a one year period, 2000-2001.

We examined all deaths due to diseases most often associated with COPD, notably, heart disease, pneumonia/influenza, and asthma. 15,026 mortality records from KY death certificates were examined for the year 2000. COPD was listed as a contributory cause of death rather than an underlying cause of death in 5.6% (845 out of 15,026) of cases studied. Number of deaths due to asthma, pneumonia and influenza was relatively small, each contributing less than 1% to the total number of death records. Overwhelmingly, the most common underlying cause of death was heart disease for which 6.8% (824 out of 12,084) cases of heart disease had COPD listed as the contributing cause of death. COPD is a chronic, often severe disease commonly associated with co-morbid conditions such as heart disease that ultimately lead to death Thus it may be more accurate to list COPD as the underlying cause of death on these death certificates to give a more accurate estimate of COPD mortality. If one includes the 845 cases described above in which COPD is listed as a contributory cause of death into the COPD age-adjusted rate for the year 2000, it increases 40%, from 52.4 to 73.2/100,000 people. COPD would then surpass cerebral vascular accidents (CVA) as the third leading cause of death in KY. Since mortality data is used to monitor and evaluate health status in terms of current mortality levels and long-term mortality and morbidity trends, it is imperative to have a true estimate of COPD mortality.

Kentucky is ranked one of the highest in the nation for COPD mortality and morbidity, and also had the highest percentage of adults who reported smoking in year 2000. The link between smoking and COPD is well established. COPD prevalence was higher in several districts in Eastern KY where smoking rates were highest. COPD is the only common cause of death that is increasing in incidence throughout the state, the nation, and the world, and although COPD continues to rise in epidemic proportions, research and new treatment modalities in this area have been surprisingly neglected. Accurate reporting is essential for health planning, education, research, and treatment options.
