# Supplementary material for: Meeting materials from the 3rd Annual Meeting of the International Society for the Prevention of Tobacco Induced Diseases
Source: Tob Induc Dis. 2004 Dec 15;2(4):168. doi: 10.1186/1617-9625-2-4-168 (PMC2671527; doi:10.1186/1617-9625-2-4-168)
Supplement: Additional file 1 [file 1617-9625-2-4-168-S1.zip › Abstract 29-Tobacco compromises periodontal health in a maintenance population.pdf]

## Abstract 29

**Tobacco compromises periodontal health in a maintenance population**

Kells L*[1], Picard J-P[1], Gelskey SC[1], Lix L[2], Singer DL[1], Scott DA[1,3]
Depts. Of DDSS[1], Community Health Medicine[2], and Oral Biology[3], University of Manitoba, Canada

**Background:** Tobacco smoke is recognized as a major risk factor for periodontal disease.

**Aim:** To examine the clinical features of periodontal destruction in smoking and non-smoking adults with periodontitis on maintenance therapy, ion a cross sectional manner.

**Subjects and methods:** Smoking status was determined by interview, and by salivary cotinine and expired-air carbon monoxide assay, in chronic periodontitis subjects (n=94, mean age 57 yrs). Probing depths and clinical attachment level were used as periodontal disease indices.

**Results:** Current smokers had deeper pockets and more attachment loss than past and never smokers (both $p < 0.05$). However, no site specific susceptibility to periodontitis levels was noted in the smoking population.

**Conclusions:** Despite maintenance therapy, smokers experience a greater amount of periodontal attachment loss and deeper probing depths, compared to non-smokers. These results confirm the negative influence of tobacco use on periodontal health and confirm the obligation of oral health professionals to provide patients with tobacco cessation advice and counseling.
