# Supplementary material for: Meeting materials from the 3rd Annual Meeting of the International Society for the Prevention of Tobacco Induced Diseases
Source: Tob Induc Dis. 2004 Dec 15;2(4):168. doi: 10.1186/1617-9625-2-4-168 (PMC2671527; doi:10.1186/1617-9625-2-4-168)
Supplement: Additional file 1 [file 1617-9625-2-4-168-S1.zip › Abstract 30-Friends, family, and the school environment a multi-level analysis of their relationship.pdf]

Abstract 30

Friends, family, and the school environment: a multi-level analysis of their relationship to student smoking behaviour

Scott Leatherdale, PhD
Cancer Care Ontario, Toronto, Ontario, Canada

The purpose of this study was to examine how older smoking peers at school and the smoking behaviour of friends and family members are related to youth smoking behaviour. Multi-level logistic regression analyses were used to examine correlates of experimental and regular smoking in a sample of 10,843 grade 9, 10 and 11 students from 29 secondary schools in Ontario, Canada. Each 1% increase in the smoking rate among high school seniors increased the odds that a junior student was an experimental smoker versus never smoker [OR 1.03, 95% CI 1.01 to 1.04], and a regular smoker versus an experimental smoker [OR 1.02, 95% CI 1.00 to 1.04]. Significant contextual interactions were found; the senior student smoking rate at a school moderates the negative influence of having close friends who smoke for both experimental and regular smokers. Findings indicate that the prevalence of smoking among older students at a school is related to both experimental and regular smokers if they have smoking friends and family members. Prevention programs should target both at-risk schools and students with prevention activities.
