# Supplementary material for: Meeting materials from the 3rd Annual Meeting of the International Society for the Prevention of Tobacco Induced Diseases
Source: Tob Induc Dis. 2004 Dec 15;2(4):168. doi: 10.1186/1617-9625-2-4-168 (PMC2671527; doi:10.1186/1617-9625-2-4-168)
Supplement: Additional file 1 [file 1617-9625-2-4-168-S1.zip › Abstract 31-Knowledge, attitudes, and clinical practices involving tobacco cessation among.pdf]

Abstract 31

Knowledge, attitudes, and clinical practices involving tobacco cessation among Kentucky physicians

J.L. Marmorato*[1], C.T. Worth[2], J.L. Studts[1], and C.L. Sorrell[2]
[1]University of Louisville School of Medicine, [2]Kentucky Cancer Program

Smoking tobacco has been identified as a prominent factor in the development of pulmonary disease, cardiovascular disease, and several cancers (e.g., lung and head/neck cancers). Although there is evidence that physicians can facilitate tobacco cessation using brief interventions, many implement cessation interventions inconsistently (Ellerbeck et al., 2003). It has been found that tobacco cessation counseling significantly increases the likelihood that patients will stop using tobacco (Fiore, Bailey, Cohen, 2000). The current study utilized data from a sample of Kentucky physicians who completed a free continuing education program, *Providers Practice Prevention: Treating Tobacco Use and Dependence*, based on the recently published United States Public Health Service Clinical Practice Guideline. Prior to participating in the program, participants completed a survey of professional practices regarding tobacco cessation, which also included questions addressing attitudes toward and knowledge of tobacco cessation. Of the physicians who completed the survey (n=356), most worked in a suburban setting, while years of experience (M=15.7, SD=11.1) and physician specialties varied greatly. Physicians strongly agreed that discussing tobacco use and encouraging tobacco cessation are important health issues for their patients. Furthermore, they demonstrated a respectable fund of knowledge related to tobacco use and cessation; however, physicians reported less confidence in their ability to treat tobacco use and nicotine dependence. While a large percentage of physicians reported that they ask their patients about tobacco use, far fewer reported that they subsequently assist their patients who are willing to quit. Only 46% of participants indicated that they “always” or “almost always” assist patients with the development of a tobacco cessation plan. In conclusion, it is clear that physicians need additional training regarding tobacco cessation. Based on these results, it is recommended that physicians receive additional tobacco cessation training as part of the medical school curriculum or continuing education programs. It is likely that applied training would provide the most benefit by targeting practical skill development, leading to increased comfort in discussing tobacco use and cessation and greater confidence in physicians’ abilities to treat nicotine dependence.
