# Supplementary material for: Meeting materials from the 3rd Annual Meeting of the International Society for the Prevention of Tobacco Induced Diseases
Source: Tob Induc Dis. 2004 Dec 15;2(4):168. doi: 10.1186/1617-9625-2-4-168 (PMC2671527; doi:10.1186/1617-9625-2-4-168)
Supplement: Additional file 1 [file 1617-9625-2-4-168-S1.zip › Abstract 32-Smoking, exposure to environmental tobacco smoke (ETS), and home policies on.pdf]

Abstract 32

Smoking, exposure to environmental tobacco smoke (ETS), and home policies on smoking in the border city of Tijuana, Mexico

Ana P. Martinez-Donate, Melbourne F. Hovell, Marc A. Adams, Jose de Jesus Sanchez, Margarita Viñas, Gabriela Guzman, Jesus R. Gonzalez, Ernesto Moreno Almaraz, M. Guadalupe Sanchez Hernandez, Oscar I. Jimenez Garcia
Center for Behavioral Epidemiology and Community Health Graduate School of Public Health, San Diego State University.

Tijuana and San Diego are twin cities at either sides of the US-Mexico border. The minimal tobacco control efforts in Mexico contrast strikingly with the comprehensive and effective California tobacco control program. As a result of the proximity and high degree of interaction between Tijuana and San Diego residents, the effects of the California program may have permeated the Mexican border city, yielding lower rates of smoking and exposure to environmental tobacco smoke (ETS) among Tijuana residents as compared to other Mexican regions. This paper presents the results of a probabilistic survey on tobacco use, ETS exposure, and residential policies on smoking among the Tijuana adult population. From June 2003 to May 2004, a household survey was carried out in Tijuana using a probabilistic sampling frame. A total of 400 adults were surveyed on current smoking, history of tobacco use, exposure to ETS, and use of home bans on smoking. A response rate of 66.5% was achieved. Weighted estimates for the overall adult population and for specific gender and age groups were computed. An estimated 22.8% (95% CI: 18.6-27.0) of the adult residents in Tijuana are current smokers, while 15.3% (95% CI: 11.7-18.9) are former smokers. Approximately 65.9% (95% CI: 61.1-70.7) of Tijuana adults live in a home where no smoking is allowed. However, 53.7% (95% CI: 48.7-58.8) of them are regularly exposed to ETS inside their home, a car, their workplace, or other places. These results are interpreted in the context of available estimates from Mexico and Latinos in the U.S. Implications for international tobacco control are discussed.
