# Supplementary material for: Meeting materials from the 3rd Annual Meeting of the International Society for the Prevention of Tobacco Induced Diseases
Source: Tob Induc Dis. 2004 Dec 15;2(4):168. doi: 10.1186/1617-9625-2-4-168 (PMC2671527; doi:10.1186/1617-9625-2-4-168)
Supplement: Additional file 1 [file 1617-9625-2-4-168-S1.zip › Abstract 33-Development of a Computer Program for Translation of human.pdf]

Abstract 33

Development of a Computer Program for Translation of human N-acetyltransferase-1 and -2 SNP data into genotype and phenotype: Applications to tobacco use risk assessment

Benjamin D. Martini, Mark A. Doll and David W. Hein
Department of Pharmacology & Toxicology and James Graham Brown Cancer Center, University of Louisville School of Medicine, Louisville, Kentucky USA

Cigarette smoke contains a large number of carcinogenic chemicals including aromatic amines such as 4-aminobiphenyl. The aromatic amines present in cigarette smoke are activated and inactivated by enzymes within each individual person. Genetic polymorphisms in these enzymes may infer genetic predisposition to cancer following exposure to cigarette smoke. N-acetyltransferase-1 (NAT1) and -2 (NAT2) are important enzymes in the metabolism of aromatic amines. Both NAT1 and NAT2 exhibit genetic polymorphism (over 25 human alleles for both NAT1 and NAT2 have been identified) in human populations primarily due to the presence of single nucleotide polymorphisms (SNPs) in their respective genes. New high throughput methods to assess the presence of these SNPs in the human NAT1 and NAT2 genes have been developed recently and have facilitated much larger molecular epidemiological studies to assess the role of NAT1 and/or NAT2 phenotype on cancer risk following cigarette smoke exposure. However, interpretation of the large data sets generated through these high throughput methods has been hindered by genotype misclassifications (e.g., Cancer Epidemiology, Biomarkers & Prevention 13: 1543-1546, 2004) and human errors inherent in manually translating SNP data to genotype and phenotype in large data sets. For example, there are over 6500 and 2100 possibilities for NAT1 and NAT2, respectively. To resolve this problem, Microsoft Visual Basic for Applications (VBA) was used to develop a computer program that accepts SNP data directly from Microsoft Excel. The program easily and rapidly converts the NAT1 and NAT2 SNP data into alleles, genotypes, and phenotypes and is quite useful in assessing the modifying effects of NAT1 and/or NAT2 genotype on cancer risk from tobacco use. The new program results in substantial decreases in time and human error. Examples of the applications of this program will be provided. Partially supported by USPHS grant CA34727 from the National Cancer Institute.
