# Supplementary material for: Meeting materials from the 3rd Annual Meeting of the International Society for the Prevention of Tobacco Induced Diseases
Source: Tob Induc Dis. 2004 Dec 15;2(4):168. doi: 10.1186/1617-9625-2-4-168 (PMC2671527; doi:10.1186/1617-9625-2-4-168)
Supplement: Additional file 1 [file 1617-9625-2-4-168-S1.zip › Abstract 34-Tobacco smoke components and metabolites alter fetal and neonatal lung cell function.pdf]

Abstract 34

Tobacco smoke components and metabolites alter fetal and neonatal lung cell function

J. Merluza*, C. Hillier, B. Weltman, P. Irani, X. Xie, D.A. Scott, and J.E. Scott
Department of Oral Biology, Faculty of Dentistry, University of Manitoba & Biology of Breathing Group, Manitoba Institute of Child Health, Children's Hospital Research Foundation, Winnipeg, Canada

At birth, the infant's lungs must be adequately mature to meet quickly the demands of gas exchange. One extremely important factor required to meet this demand is an adequate production and secretion of the pulmonary surfactant (PS). This complex phospholipid/protein mix enables inflation of the lungs and prevents their collapse at end expiration. PS is synthesized and secreted by type II alveolar cells (TII) within the lung. These cells appear at about _ of the gestational period and begin to release PS as parturition approaches. To determine the effects of smoke components (nicotine, N) and smoke metabolites (cotinine, C) on TII cells, these cells were isolated from fetal and/or neonatal rat lungs by chopping the lungs and digesting with trypsin. The cell suspension was filtered through Nitex, and two differential attachment sequences of 1h and 30m used. Cells were exposed th N (1.0×10^{-6} – 1.0×10^{-3}M) for 24hrs and the incorporation of [^{3}H]thymidine into DNA and metabolic breakdown of MTT formazan, a mitochondrial marker, measured. As well, the effects of N and C on the DNA of the TII cells were measured through the alkaline unwinding technique following single cell gel electrophoresis (Comet assay). Comets were quantitated using CASP. The results showed that N and C inhibited DNA synthesis and mitochondrial activity at doses level of 10^{-3}M in both fetal and neonatal TII cells. Furthermore, the Comet assay showed that DNA damage was induced in TII alveolar cells exposed to these agents as significant laddering of DNA was observed. These results support the hypothesis that components of tobacco smoke are detrimental to function of the surfactant producing type II alveolar cells. **Supported by the Natural Sciences and Engineering Research Council of Canada and the Manitoba Institute of Child Health.**
