# Supplementary material for: Meeting materials from the 3rd Annual Meeting of the International Society for the Prevention of Tobacco Induced Diseases
Source: Tob Induc Dis. 2004 Dec 15;2(4):168. doi: 10.1186/1617-9625-2-4-168 (PMC2671527; doi:10.1186/1617-9625-2-4-168)
Supplement: Additional file 1 [file 1617-9625-2-4-168-S1.zip › Abstract 35-Knowledge, attitudes, and clinical practices involving tobacco cessation among.pdf]

## Abstract 35

### Knowledge, attitudes, and clinical practices involving tobacco cessation among Kentucky psychologists

B.F. Miller*[1], C.S. Lotz[1], C.T. Worth2, C.L. Sorrelll[2], and J.L. Studts[3]
[1]Spalding University, [2]Kentucky Cancer Program, [3]University of Louisville School of Medicine

Approximately 80% of individuals diagnosed with sever mental illness also experience nicotine dependence. Further, tobacco use plays an important role in many other health conditions. The recently published clinical practice guideline, *Treating Tobacco Use and Dependence* (TTUD), is designed to assist clinicians to deliver effective tobacco cessation treatments. The guidelines are available for all clinicians in response to the growing need for training in the area of nicotine dependence. With training in behavior change, psychologists are uniquely qualified to provide tobacco cessation interventions which could reduce the burden of tobacco-related illness. Despite significant rates of tobacco use among the mentally ill and the general population, data regarding tobacco cessation practices among psychologists is virtually non-existent. The purpose of this study was to collect data regarding psychologists': (1) knowledge of tobacco cessation; (2) attitudes toward tobacco cessation; and (3) practice of tobacco cessation strategies. Participants included 75 licensed psychologists (40 females) in Kentucky who volunteered to participate in a free continuing education program, *Providers Practice Prevention: Treating Tobacco Use and Dependence*. Participants represented various practice settings, including urban (33%), suburban (31%), and rural (25%). Participants had practiced for an average of 14 years (SD = 11). Results indicated that study participants believed that tobacco use and cessation were important topics to discuss with patients, but only a minority of participants reported asking patients about tobacco use and subsequently advising them to quit. Despite reporting feeling comfortable discussing cessation, few psychologists reported actively engaging in the practice of tobacco cessation. Participants indicated that the major barrier to offering tobacco cessation was that they did not believe it to be a major priority for their patients. Interestingly, 2 out of every 3 psychologists sampled had not previously heard of the TTUD Guideline. These results indicate that while psychologists maintain a positive attitude toward tobacco cessation, they frequently fail to incorporate important tobacco cessation into their daily practice. Future educational programming for psychologists should be undertaken to encourage psychologists to play a more prominent role in reducing tobacco-related illness.
