# Supplementary material for: Meeting materials from the 3rd Annual Meeting of the International Society for the Prevention of Tobacco Induced Diseases
Source: Tob Induc Dis. 2004 Dec 15;2(4):168. doi: 10.1186/1617-9625-2-4-168 (PMC2671527; doi:10.1186/1617-9625-2-4-168)
Supplement: Additional file 1 [file 1617-9625-2-4-168-S1.zip › Abstract 36-Cigarette smoke condensate in oral cancer.pdf]

Abstract 36

Cigarette smoke condensate in oral cancer – apoptosis or inflammation?

Nagathihalli S. Nagaraj1, Nadaraj Vigneswaran[3], and Wolfgang Zacharias[1,2]
[1]Department of Medicine, James Graham Brown Cancer Center, [2]Department of Pharmacology & Toxicology, University of Louisville, Louisville, Kentucky 40202, USA, [3]Departments of Diagnostic Sciences, The University of Texas Health Science Center at Houston, Dental Branch, Houston, Texas 77030, USA

Oral squamous cell carcinoma (OSCC) is the most common malignancy of the head and neck, with a worldwide incidence of 300,000 new cases annually. The major inducer of OSCC is exposure to tobacco, considered to be responsible for 50-90% of cases worldwide, and the incidence of OSCC in cigarette smokers is four to seven times higher than in nonsmokers. Yet the mechanisms and key participants for this process are very poorly understood. Our aim is to identify molecular factors or events which may contribute to the pronounced increased risk of smokers for OSCC. We hypothesize that there is a discrete subset of genes responsive to cigarette smoke exposure of the oral cavity. In the present study, we are investigating the effects of cigarette smoke condensate (CSC) on different cell phenotypes: normal human epidermal keratinocytes (NHEK), oral dysplasia cell lines (Leuk 1), and primary oral carcinoma cell lines (101A). As a first step, we used MTT cell viability assays, caspase 1 and 3 activity tests, caspase 3 Western blots, and DNA fragmentation assays to evaluate possible effects of CSC on apoptosis- or inflammation-related factors. We observed CSC-dependent activation of both caspase 1 and caspase 3 in both 101A and Leuk1 cells; however, no nucleosomal DNA ladder formation was observed in any of the cell lines. As second step, we will compare differential gene expression patterns in CSC-exposed cells versus non-exposed cells by using microarray-based global expression patterns in CSC-exposed cells versus non-exposed cells by using microarray-based global gene expression profiling. Our current results suggest that CSC, at low doses of 0.01-10 ug/ml and only 5 h exposure period, is capable of inducing early apoptotic (caspase 3-mediated) as well as inflammatory (caspase 1-mediated) events in oral keratinocyte cells, with individual responses depending on the cellular phenotype. (Supported by the NIH/NIDCR, **by Philip Morris USA Inc. & Philip Morris International***, and by a postdoctoral fellowship award from the J.G. Brown Cancer Center, Univ. of Louisville, KY)

**Please read the editorial on tobacco-sponsored research in the December 2004 issue of Tobacco Induced Diseases*
