# Supplementary material for: Meeting materials from the 3rd Annual Meeting of the International Society for the Prevention of Tobacco Induced Diseases
Source: Tob Induc Dis. 2004 Dec 15;2(4):168. doi: 10.1186/1617-9625-2-4-168 (PMC2671527; doi:10.1186/1617-9625-2-4-168)
Supplement: Additional file 1 [file 1617-9625-2-4-168-S1.zip › Abstract 37-Acute nicotine treatment does not reduce blood flow to rat mandibles.pdf]

Abstract 37

Acute nicotine treatment does not reduce blood flow to rat mandibles

*Pandya, P., J.T. Fleming,a nd P.P. Rowell
Departments of Physiology and Biophysics and Pharmacology and Toxicology, University of Louisville, Louisville, KY, USA

Rationale: Smokers have a higher incidence of severe periodontal disease, failure of implant osseointegration, and soft tissue complications compared to non-smokers. Oral health is dependent on adequate blood flow to soft and osseous tissues. Though cigarettes contain over 4000 chemicals, nicotine has been shown to have more effects on the cardiovascular system than any other. This study was designed to determine if nicotine reduces blood flow to the rat mandible.

Methods: Osmotic mini-pumps were implanted subcutaneously to deliver nicotine (3.6 mg/kg/day) for 4 weeks. A second group of rats was implanted with mini-pumps to deliver nicotine-free physiologic solution. At the end of 4 weeks, the rats were anesthetized. Arterial blood was collected to measure plasma nicotine levels. Arterial pressure was measured directly from the carotid artery. Blood flow to the mandibles was measured by the classical microsphere technique. Withdrawal of blood from the tail artery at a rate of 0.5 ml/min served as the “phantom limb”. The number of microspheres in each mandible was determined by neutron activation.

Results: Plasma nicotine in the treated rats was 28.5 ng/ml, compared to 0/45 ng/ml in the saline-treated rats (129 ± 3 mmHg and the untreated rats (129 ± 7 mmHg). Blood flow to the mandibles of nicotine-treated rats (0.23 ± 0.01 ml/min) was not different from flow in control rats (0.29 ± 0.18 ml/min).

Conclusion: Nicotine treatment for 4 weeks does not reduce blood flow to healthy mandibles of rats. A longer period of nicotine exposure (similar to the duration of nicotine consumption in humans) may be required to evoke significant changes in mandibular blood flow in rats.
