# Supplementary material for: Meeting materials from the 3rd Annual Meeting of the International Society for the Prevention of Tobacco Induced Diseases
Source: Tob Induc Dis. 2004 Dec 15;2(4):168. doi: 10.1186/1617-9625-2-4-168 (PMC2671527; doi:10.1186/1617-9625-2-4-168)
Supplement: Additional file 1 [file 1617-9625-2-4-168-S1.zip › Abstract 38-Effects of Tetracyclines on Biomarkers of Systemic Inflammation in Smokers.pdf]

Abstract 38

Effects of Tetracyclines on Biomarkers of Systemic Inflammation in Smokers with Periodontitis

Simon, S., Roemer, E., Carnu, O., Farrell, J., Tenzler, R. and Ryan, M.E.
Stony Brook University, Stony Brook, New York.

Increasing evidence suggests that the link between smoking and cardiovascular (CV) disease may involve an intermediary role of periodontitis. We hypothesize that the combination of smoking and periodontal disease acts as a "double hit" to trigger a systemic inflammatory response. Based on *in vitro* and *in vivo* studies in other "double hit" models of systemic inflammation, treatment with tetracycline derivatives can reverse this upregulation of the inflammatory response and should thereby lower the risk of CV complications. A double-blinded placebo controlled randomized clinical trial in smokers with periodontal disease being treated with scaling and root planing and orally administered sub-antimicrobial dose doxycycline (20mg bid, Periostatθ) or placebo for nine months continuously followed by a six month washout is nearing completion. All subjects receive maintenance periodontal therapy every 3 months, at which times plasma, serum, oral mouth rinse, saliva and gingival crevicular fluid (GCF) samples are obtained and clinical measurements of pocket depth and attachment levels are recorded. Plasma samples are analyzed on an ongoing basis for levels of inflammatory biomarkers and the nicotine metabolite, cotinine by ELISA. Biomarker evaluation is based on analysis of 138 plasma samples collected at baseline and at 3- month intervals in patients recruited on a rolling basis for levels of cotinine, sICAM, IL-8, IL-1_, C-reactive protein (CRP), IL-6, haptoglobin, and TNF-_. Stratification of the samples into quartiles with respect to cotinine levels reveals strong correlation of cotinine to sICAM and IL-8 levels as well as to total GCF fluid volumes within all quartiles but not to CRP or the other biomarkers. On the other hand, stratification with respect to CRP reveals a different correlation within all quartiles, to IL-6 and haptoglobin levels, but not to the other biomarkers or cotinine. These results suggest that sICAM and CRP, which have both been correlated to risk of CV complications, may be regulated by independent mechanisms. (**Supported by Philip Morris External Research Program***)

**Please read the editorial on tobacco-sponsored research in the December 2004 issue of Tobacco Induced Diseases*
