# Supplementary material for: Meeting materials from the 3rd Annual Meeting of the International Society for the Prevention of Tobacco Induced Diseases
Source: Tob Induc Dis. 2004 Dec 15;2(4):168. doi: 10.1186/1617-9625-2-4-168 (PMC2671527; doi:10.1186/1617-9625-2-4-168)
Supplement: Additional file 1 [file 1617-9625-2-4-168-S1.zip › Abstract 39-Association of smoking with increasing vascular involvement in Type 2 diabetic.pdf]

Abstract 39

Association of smoking with increasing vascular involvement in Type 2 diabetic Chinese patients

GN Thomas[1]*; B Tomlinson[2]; Sarah M McGhee[1]; ASM Abdullah[1]; V Yeung[2], JCN Chan[2] and KS Wong[2].

Department of Community Medicine[1], University of Hong Kong, Pokfulam, Hong Kong SAR
Department of Medicine and Therapeutics[2], The Chinese University of Hong Kong, The Prince of Wales Hospital, Shatin, Hong Kong

Objectives: To identify the relationship of between smoking and the metabolic profile and existing vascular disease in Chinese Type 2 diabetic patients.

Methods: 1710 Type 2 diabetic patients were identified and screened for complications, and biochemical and anthropometric vascular risk factors. As most smokers were male, anthropometric and fasting biochemical parameters were compared between male current (n=196) and never smoking patients (n=300). The prevalence of concomitant vascular diseases was also compared between the groups.

Results: The smokers had higher glycosylated haemoglobin levels (8.2±2.0 vs 7.6±1.8%, p<0.001) than never smokers, despite a greater proportion receiving hypoglycaemic agents (87.5 vs 79.6%, p=0.003). Male smokers also had a worse lipid profile compared to never smokers with lower HDL-cholesterol levels (1.12±0.31 vs 1.20±0.30 mmol/L, p=0.006), evidence of renal damage with elevated albumin-to-creatinine ratio (3.57 (2.68-4.75) vs 2.47 (1.99-3.05) mg/mmol, p=0.040), although the proportion with micro or macroalbuminuria did not differ significantly between the groups. However, diastolic blood pressure was lower in the smoking group (78±12 vs 82±12 mm Hg, p=0.001) even though the proportion receiving blood pressure-lowering therapy was also lower (23.8 vs 33.2%, p=0.034). The prevalence of peripheral vascular disease was increased in the diabetic patients who smoked (7.1 vs 2.8%, p=0.039).

Conclusions: Smoking was associated with a more adverse metabolic profile and a greater proportion of patients with peripheral vascular disease. As the mainland Chinese population is undergoing rapid modernisation and urbanisation, the observed effects of smoking in the urbanised Hong Kong population means tobacco control both in Hong Kong and in particular in mainland China becomes increasingly important to prevent or minimise potential health impacts.
