# Supplementary material for: Meeting materials from the 3rd Annual Meeting of the International Society for the Prevention of Tobacco Induced Diseases
Source: Tob Induc Dis. 2004 Dec 15;2(4):168. doi: 10.1186/1617-9625-2-4-168 (PMC2671527; doi:10.1186/1617-9625-2-4-168)
Supplement: Additional file 1 [file 1617-9625-2-4-168-S1.zip › Abstract 40-Knowledge, attitudes, and clinical practices involving tobacco cessation among.pdf]

Abstract 40

Knowledge, attitudes, and clinical practices involving tobacco cessation among Kentucky dentists

*S.D. Thomas[1], C.T. Worth[2], J.L. Studts[1], and C.L. Sorrell[2]
[1]University of Louisville School of Medicine, [2]Kentucky Cancer Program

In the United States, tobacco use is the leading preventable cause of illness and death, and Kentucky maintains the highest smoking rate in the country (32%). Dentists could play an influential role in assisting patients to quit using tobacco since approximately half of all smokers have annual dental checkups. The American Dental Association's position is that all members are urged to become "fully informed" about tobacco cessation intervention techniques. The current study surveyed professional practices of Kentucky dentists (n=266) to determine if they consistently identify patients that use tobacco, advise them to quit, and offer assistance to overcome nicotine dependence. Kentucky dentists were asked to complete the survey as part of a free continuing education program, *Providers Practice Prevention: Treating Tobacco Use and Dependence*, which reflects the United States Public Health Service Clinical Practice Guideline's five A's (Ask, Advise, Assess, Assist, Arrange). The majority of the dentists worked in rural (44%) or suburban (36%) areas and two-thirds were male. The evidence from the current study indicates that dentists do not routinely intervene to assist patients in tobacco cessation. Although a majority of dentists agree that it is a professional responsibility to educate patients about the oral health hazards of tobacco use and that tobacco cessation should be discussed with patients during dental visits, few dentists actually comply with this belief. Remarkably, 63% of dentists surveyed reported that they either "rarely" or "never" assist patients with a tobacco cessation plan. One possible reason for this discrepancy is that almost one-third of the dentists indicated they did not have sufficient skills and knowledge to treat nicotine dependence, and half felt uncomfortable discussing tobacco cessation with patients. Another troubling discovery was that less than 30% of dentists indicated strong agreement that a brief intervention with patients could be effective. Most dental schools have not yet systematically incorporated tobacco cessation into the curriculum. To help integrate tobacco cessation interventions as part of routine dental care, it would be optimal for dental schools to amend the curriculum to offer students educational training and to develop continuing education programs for practicing dentists.
