# Supplementary material for: Meeting materials from the 3rd Annual Meeting of the International Society for the Prevention of Tobacco Induced Diseases
Source: Tob Induc Dis. 2004 Dec 15;2(4):168. doi: 10.1186/1617-9625-2-4-168 (PMC2671527; doi:10.1186/1617-9625-2-4-168)
Supplement: Additional file 1 [file 1617-9625-2-4-168-S1.zip › Abstract 41-Predictors of Smoking Initiation and Smoking Patterns Among College Students.pdf]

Abstract 41

Predictors of Smoking Initiation and Smoking Patterns Among College Students: Personality Factors, Sense of Coherence, Self-Efficacy, & Social Support

Diane Von Ah, PhD, RN, University of Louisville, School of Nursing & Sheryl Ebert, PhD Candidate, University of Alabama at Birmingham, Department of Psychology

Background & Significance: Although cigarette smoking among adults has steadily declined over the past decade, smoking among college students has risen sharply (Kear, 2002). Approximately, one quarter of all college students in the U.S. smoke (Martinelli 1999), and 75% of those continue to smoke into later adulthood (Flay 1993), placing them at greater risk of lung and cardiovascular diseases. Similar trends were noted in Western and Eastern European university students, with 22.9% and 19.8% reporting being regular smokers, respectively (Steptoe and Wardle, 2001). Thus determinants of smoking behavior in this population must be elucidated so that interventions can be better targeted and more effective.

Purpose: The purpose of this study was to examine the impact of demographic information (gender & race) personality factors (neuroticism, extraversion, openness, agreeableness, and conscientiousness), sense of coherence, self-efficacy social support (family and friend support) on smoking behavior (initiation, frequency, and amount of cigarette smoking).

Method: A cross-sectional sample of 161 college students completed self-report questionnaires. Multiple regression analysis was conducted to determine the impact of predictor variables on smoking behavior.

Results: A majority of the students reported having tried smoking (55%); among those that had tried, 43% were current smokers. The majority (77%) who had smoked an entire cigarette did so at age 16 years or younger. Students who reported lower levels of conscientiousness and lower self-efficacy were more likely to report having tried cigarette smoking than those with higher levels. In addition, students who had lower levels of family support and lower self-efficacy reported smoking more frequently. Similarly, students who reported lower self-efficacy reported smoking greater quantities of cigarettes.

Conclusions: Self-efficacy was the most significant predictor of smoking behavior. Future health promotion programs must focus on interventions that maximize self-efficacy to reduce the initiation, frequency, and amount of smoking of college students.
