# Supplementary material for: Meeting materials from the 3rd Annual Meeting of the International Society for the Prevention of Tobacco Induced Diseases
Source: Tob Induc Dis. 2004 Dec 15;2(4):168. doi: 10.1186/1617-9625-2-4-168 (PMC2671527; doi:10.1186/1617-9625-2-4-168)
Supplement: Additional file 1 [file 1617-9625-2-4-168-S1.zip › Abstract 42-Fetal mandibular condyle cell Matrix Metalloproteases (MMPs) secretions are altered by.pdf]

Abstract 42

Fetal mandibular condyle cell Matrix Metalloproteases (MMPs) secretions are altered by tobacco smoke components and metabolites

B. Weltman,* J. Merluzza, C. Hillier, P. Irani, X. Xie, D.A. Scott, and J.E. Scott
Department of Oral Biology, Faculty of Dentistry, University of Manitoba & Manitoba Institute of Child Health, Children's Hospital Research Foundation, Winnipeg, Canada

Introduction: During the smoking process, nicotine and cotinine levels rise precipitously in the blood and both readily cross the placenta in smoking pregnant women. Both nicotine and cotinine may alter cellular metabolic processes and thus have the potential to affect rapidly replicating or very metabolically active tissues of the fetus. In the present study we used fetal mandibular condyle cells as a model to determine the affects of tobacco smoke components and metabolites on cellular specialization.

Methods: Rabbit fetal mandibular condylar cells were isolated and grown in culture. Briefly, mandibular condyles from 24th gestational day fetal rabbits were trypsinized, inverted on gelfoam sponge and cells allowed to grow into the sponge. After several days the sponge was dissolved with collagenase, the cells collected, repeatedly passaged and exposed to IL-1β (0.1ng/ml), or nicotine, or cotinine (10^{-7} to 10^{-4}M), or dioxin (10^{-12} to 10^{-9}M). Single strand DNA breaks were estimated using the COMET assay. Cell vitality was examined with the formazan assay and [^{3}H] thymidine incorporation. Matrix Metalloprotease (MMP) activity was measured by molecular mass gel zymography.

Results: The highest concentrations of nicotine and cotinine reduced DNA synthesis and mitochondrial activity. Zymography showed that these condylar fibroblasts secreted MMP-1, 02, -3, -9 and further, that the pattern of expression was altered by exposure to tobacco smoke components and metabolites. Il-1β and cotinine increased MMP-9 and MMP-2 secretion over 24hours.

Conclusion: MMP's are involved in tissue remodeling during fetal development. It appears that components in tobacco smoke can dysregulate MMP production and alter cellular metabolism and replication rate. These effects may compromise aspects of fetal development, and thus contribute to inappropriate development *in utero*.

Supported by NSERC and Manitoba Institute of Child Health
