# Supplementary material for: Meeting materials from the 3rd Annual Meeting of the International Society for the Prevention of Tobacco Induced Diseases
Source: Tob Induc Dis. 2004 Dec 15;2(4):168. doi: 10.1186/1617-9625-2-4-168 (PMC2671527; doi:10.1186/1617-9625-2-4-168)
Supplement: Additional file 1 [file 1617-9625-2-4-168-S1.zip › Abstract 43-Treating tobacco use and dependence.pdf]

Abstract 43

Treating tobacco use and dependence: One year follow-up of the Providers Practice Prevention Program

* Celeste T. Worth[1], Jamie L. Studts[2], and Connie L. Sorrell[1]
[1]Kentucky Cancer Program-University of Louisville, [2]University of Louisville School of Medicine

Kentucky leads the United States in smoking prevalence as well as overall lung cancer morbidity and mortality. The United States Public Health Service Clinical Practice Guideline, *Treating Tobacco Use and Dependence (TTUD)*, provides evidence-based recommendations for clinicians to promote tobacco cessation and reduce the burden of tobacco-related illness. The Providers Practice Prevention: Treating Tobacco Use and Dependence (PPP-TTUD) program is a self-study continuing education offering for practicing physicians, dentists, and psychologists in Kentucky. The program includes a 30-minute video and a clinician tool-kit which provides information for clinicians and their patients regarding tobacco use assessment and counseling. As part of the offering, participants responded to three surveys of their knowledge, attitudes, and practices regarding tobacco cessation. Participants completed a practice survey (pre-test) prior to reviewing the program, a program evaluation (post-test) immediately following the program, and a follow-up survey (follow-up) one year after program completion. Of the 712 participants who completed the pre and post-tests, 427 (60%) also completed the follow-up questionnaire. Participants included 357 physicians, 275 dentists, and 75 psychologists, practicing in rural (39%), suburban (38%), and urban areas (19%), and representing a wide range of practice experience (1-54 years). Immediately following participation in the PPP program, participants reported dramatic increases in tobacco cessation knowledge, more confidence in their tobacco cessation skills, and greater intentions to adopt clinical practice guideline recommendations. Results of the follow-up survey showed that the intervention significantly promoted tobacco cessation practices among this group of clinicians, although the strength of the intervention lessened over time. Overall, the PPP-TTUD program appeared to promote the integration of the TTUD recommendations into the clinical practice of participating licensed health care providers. To enhance the effects of future interventions of this nature, researchers/educators might consider incorporating follow-up information to help clinicians maintain their initial motivation and knowledge, while expanding their skills in treating tobacco dependence.
