# Supplementary material for: Meeting materials from the 3rd Annual Meeting of the International Society for the Prevention of Tobacco Induced Diseases
Source: Tob Induc Dis. 2004 Dec 15;2(4):168. doi: 10.1186/1617-9625-2-4-168 (PMC2671527; doi:10.1186/1617-9625-2-4-168)
Supplement: Additional file 1 [file 1617-9625-2-4-168-S1.zip › Abstract 44-How do daily and occasional smokers differ in quit behaviour.pdf]

Abstract 44

How do daily and occasional smokers differ in quit behaviour?

Anne Marie Zaborski, B.A., Senior Survey Methodologist, Office of Research, Surveillance and Evaluation, Tobacco Control Programme, Health Canada

Judy Snider, M.Sc., Manager of Surveillance, Office of Research, Surveillance and Evaluation, Tobacco Control Programme, Health Canada

Caren Uhlik, B.P.E., Analyst, Office of Research, Surveillance and Evaluation, Tobacco Control Programme, Health Canada

The Canadian Tobacco Use Monitoring Survey (CTUMS) is an ongoing national survey of over 20,000 Canadians 15 years and older. Data have been collected on quitting behaviours including intention to quit within 30 days and within 6 months, and methods used in recent attempts.

Over the past 5 years, smoking rates among Canadians 15 years and older have decreased from 25% to 21%. Declines were noted in the rates among daily smokers (from 21% to 17%) but rates have remained constant at 4% among occasional smokers. Two-thirds of occasional smokers report having ever been daily smokers. No difference was noted in the stated intentions to quit in the long term (within 6 months) between occasional and daily smokers (55% vs 57%), however in the short term, a substantially higher percentage of occasional smokers stated an intention to quit within 30 days than daily smokers (70% as compared to 44%).

When asked about methods used in their quit attempts over the previous 2 years, reducing the number of cigarettes smoked per day was most often cited by both occasional and daily smokers (67%). A higher percentage of daily smokers reported using the patch (44%), gum (28%) and 'a product like Zyban' (29%) as quit methods compared to 20%, 15% and 15% respectively of occasional smokers.

Research targeted at untangling the composition of the occasional smoking group should be undertaken to better understand the dynamics of this group, specifically the daily smokers who evolve into occasional smokers on the road to quitting, and those who initiate as occasional smokers and remain in this group over time. Results from future studies can further assist cessation programs to tailor different approaches and strategies for people in each of these subgroups.
