# Supplementary material for: Meeting materials from the 3rd Annual Meeting of the International Society for the Prevention of Tobacco Induced Diseases
Source: Tob Induc Dis. 2004 Dec 15;2(4):168. doi: 10.1186/1617-9625-2-4-168 (PMC2671527; doi:10.1186/1617-9625-2-4-168)
Supplement: Additional file 1 [file 1617-9625-2-4-168-S1.zip › Abstract 45-The influence of nicotine on microlymphatics in rat mesentery.pdf]

Abstract 45

The influence of nicotine on microlymphatics in rat mesentery

V.P. Zharov[a], E.I. Galanzha [a,b], *P. Chowdhury [c], V.V. Tuchin [b]
[a]Philips Classic Laser Laboratories, University of Arkansas for Medical Sciences (UAMS), Little Rock, AR, 72205
[b]Saratov State University, Saratov, Russia
[c]Department of Physiology and Biophysics, UAMS, Little Rock, AR, 72205

In recent years, the pathological effects of nicotine on the blood microcirculation have been reported, while the disturbances of lymphatic system remain obscure. The aim of the current study was to identify the nicotine induced response in microlymphatics of rat mesentery with an ultimate goal to evaluate its mechanism of action. METHODS: The microlymphatics of the intestinal mesentery of narcotized Male Sprague Dawley rats have been studied *in vivo* by integrated optical imaging with focus on high resolution digital light transmission microscopy. This technique allowed visualizing, at different magnifications the entire lymphangion, as well as lymph and blood microvessels, their walls, lymphatic valves, lymphocyte motion in lymph flow, and relatively slow-moving single cells in blood and lymph capillaries, with highest resolution up to 300-350 nm. The influence of nicotine on microlymphatics was determined at three nicotine administrations: 1) direct topical application in various concentrations during 15 min; 2) after acute injection of various concentrations through the cannulated vein; and 3) and chronic administration for 14 days via subcutaneous injection of mini-osmotic pumps (0.5 μl/h delivery rate). RESULTS: The topical application showed that the most significant response of microlymphatics on nicotine impact was at the doses of 10 mM and 100 mM. In particular, the concentration of 10 mM caused the significant immediate short-term constriction of 100% lymphangions. The effect started within 3-5th s of application (67% of cases) or after ~3 min of nicotine action (33% of cases). The duration of effect was 12-40 s. The lymphatic diameter was decreased on 34±7% (this is more in ~2 times than amplitude of short-time contraction – phasic contraction – in intact state of lymphangions before nicotine application). With highest concentration of nicotine (100 mM), marked immediate constriction in all cases (starting with 3-5 s, duration – 240-40 s, mean degree of constriction 28±8%), the slowing of lymph flow, local stable constriction of lymph microvessels, asynchronous motion of lymphatic wall, the stasis in blood microvessels and disturbances of respiration was observed. Acute intoxication at the intravenous introduction of nicotine caused a little relaxation of lymphatics with sometimes a slowing of lymph flow and short-term stasis in blood microvessels. The chronic nicotine intoxication in rats did not change markedly the function of lymphatic and blood microvessels and did not differ markedly from that in the intact state. SUMMARY AND CONCLUSIONS: Results indicate that the significant macro-changes of small lymphatic function under direct nicotine impact occurred *in vivo* which was dose and time dependent. We hypothesize that the obtained effects is the result of direct action of nicotine of lymph microvessels and reflect, probably, specific endothelial dysfunction and/or injury of contractile ability of lymphatic wall. The absence of effects at the chronic intoxication may be the result of adaptation of the microcirculation to the action of nicotine. The significance of these facts and the role and sensitivity of small lymphatics neighboring blood microvessels remains to be investigated further.
