# Supplementary material for: Meeting materials from the 3rd Annual Meeting of the International Society for the Prevention of Tobacco Induced Diseases
Source: Tob Induc Dis. 2004 Dec 15;2(4):168. doi: 10.1186/1617-9625-2-4-168 (PMC2671527; doi:10.1186/1617-9625-2-4-168)
Supplement: Additional file 1 [file 1617-9625-2-4-168-S1.zip › ISPTID 2003 Conference Details.pdf]

Congress President

Dr. Denis F. Kinane, University of Louisville

Local Organizing Committee

Dr. David Hein, University of Louisville
Dr. David Tollerud, University of Louisville, Kentucky
Dr. William Wead, University of Louisville, Kentucky
Dr. Christine Ritchie, University of Birmingham, Alabama

PTID Society

International Society for the Prevention of Tobacco Induced Diseases

Society Officers

**Dr. David A. Scott,**<br>**University of Manitoba, Canada**	***President***
**Dr. Anthony J. Hedley,**<br>**The University of Hong Kong**	***Vice-President & Director of Australasia Division***
**Dr. Wojciech Hanke,**<br>**Nofer Institute of Occupational Medicine, Poland**	***Treasurer & Director of the European Division***
**Dr Thanos Zavras, Harvard University, USA**	***Director of the America's Division***

Executive Committee

Dr. Daniel Longo, University of Missouri-Columbia, USA

Dr. Sophia S. Chan The University of Hong Kong

Dr. Tai H. Lam The University of Hong Kong

Dr. Graziella Filippini, National Institute of Neurology, Italy

Dr. Maria Teresa Zenzes, University of Toronto

Dr. Karl Erik Lund, National Institute of Public Health, Norway

Dr. Gideon Koren, The Hospital for Sick Children, Canada
