# Supplementary material for: Meeting materials from the 3rd Annual Meeting of the International Society for the Prevention of Tobacco Induced Diseases
Source: Tob Induc Dis. 2004 Dec 15;2(4):168. doi: 10.1186/1617-9625-2-4-168 (PMC2671527; doi:10.1186/1617-9625-2-4-168)
Supplement: Additional file 1 [file 1617-9625-2-4-168-S1.zip › Abstract 1-The magnitude of health problems related to tobacco use.pdf]

Abstract 1

Saturday 08.40

The magnitude of health problems related to tobacco use

Kinane, DF
Oral Health and Systemic Disease, University of Louisville School of Dentistry

Smoking has over 50 ways to make your life miserable through illness and more than 20 ways of killing you. Based on current CDC statistics, 46 million adults (22.5%) in the US are smokers and smoking predominates among the poor and less well educated. Indeed smoking, although relatively expensive, is more common (33%) below rather than above the poverty line (22%). Smoking increases risk for many medical conditions, some of which are fatal while others merely cause years of debilitating illness or other problems. Smokers in all populations endure poorer health than non-smokers and at least half of teenage smokers will die from diseases caused by tobacco if they continue to smoke. It is estimated that the more severely affected 25% of smokers will lose on average 21 years of life. Between 1950 and 2000, 60 million people worldwide have died from tobacco-related diseases. Data from Britain indicates that deaths caused by smoking are five times higher than deaths arising from: traffic accidents; overdose; alcoholic liver disease; other accidental deaths; murder and manslaughter; suicide; and HIV infection combined. World-wide, almost 5 million people now die prematurely each year as a result of smoking. Based on current trends, this will rise to 10 million within 20 years. Thus a strong theme of this conference 'tobacco kills', is extremely apt as is the positive preventive theme, 'tobacco cessation'. Nicotine is the addictive substance driving tobacco use but the other substances generated from tobacco use cause the fatal and non fatal diseases and these need to be better understood. 'Noxious aspects of tobacco use' is a further theme of this international conference. The expert speakers assembled will address the more prominent disease conditions and update us on current understanding of these issues.
