# Supplementary material for: Meeting materials from the 3rd Annual Meeting of the International Society for the Prevention of Tobacco Induced Diseases
Source: Tob Induc Dis. 2004 Dec 15;2(4):168. doi: 10.1186/1617-9625-2-4-168 (PMC2671527; doi:10.1186/1617-9625-2-4-168)
Supplement: Additional file 1 [file 1617-9625-2-4-168-S1.zip › Abstract 2-The growing consumption of tobacco in China.pdf]

Abstract 2

Saturday 09.20

The growing consumption of tobacco in China: Free trade and British American Tobacco as a vector for tobacco induced disease in the PRC

AJ Hedley
Department of Community Medicine, University of Hong Kong, China

China has 320 million of the world's 1.3 billion smokers, and consumes 1.8 trillion (1.8×10^{12}) cigarettes annually. There are now 1 million tobacco deaths per annum projected to increase to 3 million by 2050. Since 1979 the People's Republic has made tobacco control part of the legal system with 22 laws and other regulations. Bans on smoking in public places have been introduced in municipalities, provinces and autonomous regions. However the Government's interest in tobacco also relates to the income of the State Tobacco Monopoly Administration (STMA) ($30 billion profit in 2003) which generate one tenth of all tax revenues. China's membership of the WTO includes concessions on the marketing of cigarettes in China. In July 2004 British American Tobacco announced agreement on the building of a factory costing to produce 100 billion (1×10^{11}) cigarettes annually, dwarfing the output of BAT's 180 other plants world-wide and leading to a 5% share of China's tobacco sales. Recently STMA officials denied the deal but BAT CEO Paul Adams said "We are committed to China for the long term". Tobacco control advocates should target all organizations, including the British Government and chambers of commerce, which may facilitate BAT's penetration of China and other potential emerging tobacco markets.
