# Supplementary material for: Meeting materials from the 3rd Annual Meeting of the International Society for the Prevention of Tobacco Induced Diseases
Source: Tob Induc Dis. 2004 Dec 15;2(4):168. doi: 10.1186/1617-9625-2-4-168 (PMC2671527; doi:10.1186/1617-9625-2-4-168)
Supplement: Additional file 1 [file 1617-9625-2-4-168-S1.zip › Abstract 3-Secondhand Smoke Exposure.pdf]

Abstract 3

Saturday 09.50

Secondhand Smoke Exposure

S.M. McGhee, A.J. Hedley
Department of Community Medicine, University of Hong Kong, Hong Kong SAR, China

The smoke from a burning cigarette contains a toxic mix of chemical substances and the IARC has classed secondhand tobacco smoke (SHS) as a Group 1 carcinogen. The evidence of harm to adult passive smokers is mounting including lung cancer and respiratory illness, heart disease and stroke as well as more minor acute respiratory symptoms and irritation. Children are even more vulnerable with domestic exposures before and/or after birth resulting in low weight infants, chronic health problems and more frequent hospital admissions for respiratory disease. There is also evidence of damage to lipoproteins in children although much of the longer term harm has not yet been assessed.

For adults, the workplace has been a common site of exposure but, in many countries, smoke-free workplaces are becoming more common. In the developing world however, SHS remains as occupational hazard, In Hong Kong, non-smoking catering workers had urine cotinine levels which indicated a 3% increased risk of mortality from heart disease or lung cancer compared with a level of 0.1% for the US occupational health significant risk level.

Policy-makers everywhere need to protect non-smokers from this serious threat to their respiratory and cardiovascular health and thus protect their communities from serious economic loss.
