# Supplementary material for: Meeting materials from the 3rd Annual Meeting of the International Society for the Prevention of Tobacco Induced Diseases
Source: Tob Induc Dis. 2004 Dec 15;2(4):168. doi: 10.1186/1617-9625-2-4-168 (PMC2671527; doi:10.1186/1617-9625-2-4-168)
Supplement: Additional file 1 [file 1617-9625-2-4-168-S1.zip › Abstract 4-The 2004 Ed Nelson Keynote Lecture.pdf]

Abstract 4

Saturday 11.10

The 2004 Ed Nelson Keynote Lecture

Tobacco litigation vs. grass roots to a smoke free society: Past and future

J.S. Wigand, PhD, MAT
Smoke-Free Kids

There are multiple approaches to the *denormalization* of tobacco that require a consistent community based tobacco control practice, coupled with appropriate state and federal laws. For more than 200 years, tobacco has been an endemic in virtually every environment in America -- from classrooms, doctor's lounges, and patient's rooms to corporate board rooms. Beginning as a fundamental underpinning of the United States' economy, tobacco has become a ubiquitous and accepted part of our society, even though it exacts enormous economic, social and human suffering tolls.

The tobacco industry's enormous profit margins have had several deleterious effects. These monies 1) have supported strong political ties that insulate the industry from any substantive regulations usually associated with any other consumer product; 2) have given the industry the ability to engage in billion dollar marketing and sales programs that target a number of "vulnerable" groups: women, the youth and the marginally educated; 3) have financed multiple high powered law firms in their development of elaborate and convoluted schema to insulate the public from controversial internal company documents that compromise the legal integrity of the industry.

In the 1990's, litigation initiated by both the states' Attorneys General and a consortium of highly successful private plaintiff lawyers unearthed an unprecedented archeology of secret internal documents. These documents unveiled decades of lawyer crime fraud, scientific obfuscations, consumer fraud, and a total disregard for public health and safety for economic gain. Initially these lawsuits yielded an unprecedented surrender by the tobacco industry that encompassed a $368 billon dollar payout, unfettered FDA regulation, production of internal company documents, and a virtual restriction of all product advertising and promotion as well as other tobacco provisions. It had only one caveat...the agreement reach on 20 June 1997 by the 39 states Attorneys General had to obtain US congressional approval. It failed in less than three months to get Congressional approval, due, in part, to the industry's lobbyists who exploited the notion of "free choice," and argued that there would be extensive job loss if Congress approved the deal.

In November 1998, the tobacco industry capitulated by agreeing to pay the remaining 40 states and territories $246 billion dollars over a 25 year period. This money was intended to 1) pay for the past costs absorbed by the states in treating sick smokers, and to 2) launch a major tobacco control and prevention programs directed at the 1 million children that become tobacco addicts each year in the USA. Over 90% of all adult smokers start well before the legal age of 18 with the average age in North America of 13 years old. The 100% preventable death toll is 460,000 in the USA and over 5.1 million world wide.

Denormalization strategies of counter advertising, cessation, surveillance, youth education, stiff price increases, smoke free environments, product regulation, removal of tobacco advertising from public venues, graphic product labeling, deglamorizing the product on the silver screen as well as other tobacco control measures will be discussed. CDC's Best Practices and Canada's approaches have shown some impacts but much still needs to be done.

In 1999 the US Department of Justice filed a lawsuit against the tobacco industry under the RICO federal law. The law has both civil and criminal aspects used almost exclusively for prosecuting gangsters and organized crime. The DOJ seeks to disgorge the tobacco industry of ill gotten profits of $280 billion as well as other remedies. This lawsuit will use over 40 million documents from the industry's files, over 78,000 exhibits, demonstrate over 116 racketeering acts committed by the industry spanning five decades and stems from the Australian Rolah McCabe case. This law suit started on 21 September 2004 and is expected to last well into next year.

This presentation is named in honour of the Society's founder, who passed away in the summer of 2002.
