# Supplementary material for: Meeting materials from the 3rd Annual Meeting of the International Society for the Prevention of Tobacco Induced Diseases
Source: Tob Induc Dis. 2004 Dec 15;2(4):168. doi: 10.1186/1617-9625-2-4-168 (PMC2671527; doi:10.1186/1617-9625-2-4-168)
Supplement: Additional file 1 [file 1617-9625-2-4-168-S1.zip › Abstract 5-Genetic variation Clues as to how tobacco and its products endanger the user.pdf]

Abstract 5

Saturday 13.40

Genetic variation: Clues as to how tobacco and its products endanger the user

Philip Lazarus, Yan Zheng, Doris Wiener, Jia-Long Fang, and Aaron Runkle
Penn State Cancer Institute, Department of Pharmacology, Penn State College of Medicine, Hershey, PA

Genetic variation is thought to play a major role in determining susceptibility to tobacco-induced carcinogenesis. Polymorphisms in a number of enzymes involved in the metabolism of tobacco carcinogens may be linked to phenotypic differences in enzyme activity or expression and to alterations in tobacco-related cancer risk. The UDP-glucuronosyltransferase (UGT) family of enzymes are responsible for the glucuronidation and detoxification of several major tobacco/tobacco smoke carcinogens including metabolites of benzo(a)pyrene (BaP) and the nicotine-derived tobacco-specific nitrosamine, 4-(methylnitrosamino)-1-(3-pyridyl)-1-butanone (NNK). The specific UGTs responsible for the glucuronidation of several of these metabolites have been identified, a number of polymorphisms in the genes coding identified, and a number of these polymorphisms have been studied as to their importance in tobacco-related cancer risk. The goal of the present studies was to demonstrate that potentially important UGT polymorphisms significantly affect tobacco carcinogen glucuronidation capacity in human tissues. For these studies, we examined UGT genotypes in a series of normal human liver specimens (n=96) obtained from subjects undergoing surgical resection for hepatocellular carcinoma, correlating with glucuronidation capacity of the procarcinogen, 4-(methylnitrosamino)-1-(3-pyridyl)-1-butanol (NNAL), the major metabolite of NNK, or BaP-7,8-dihydrodiol (BPD), precursor to the ultimate carcinogen of BaP, BaP-7,8-diol-9,10-epoxide. NNAL glucuronidation is performed primarily by 4 UGT enzymes: UGT1A4, which primarily forms NNAL-*N*-Gluc, and UGTs 1A9, 2B7, and 2B17, which primarily form NNAL-*O*-Gluc. Relatively high-prevalence amino acid-changing polymorphisms have been identified for UGT1A4 (codon 24) and UGT2B7 (codon 268). Significant ($p<0.05$) associations between genotypes for both UGT1A4 and UGT2B7 and altered NNAL-glucuronidating activities were observed in liver microsomes from these subjects. Similarly, a newly-identified polymorphic deletion in the UGT2B17 gene was also significantly ($p<0.005$) correlated with altered liver microsomal NNAL-glucuronidating activities. One of the major UGTs involved in the glucuronidation of BPD is UGT1A1. The Gilbert Syndrome-linked UGT1A1*28 allelic variant contains an additional (TA) dinucleotide repeat $[(TA)_6>(TA)_7]$ in the 'TATAA' box of the transcriptional promoter of the BPD-glucuronidating UGT1A1 gene. Significant ($p<0.05$) decreases in overall UGT1A1 protein levels ($p<0.005$), bilirubin conjugation activity ($p<0.001$), and BPD(-) glucuronidation activity ($p<0.02$) were observed in *in vitro* assays using liver microsomes from individual subjects homozygous for the UGT1A1*28 allelic variant as compared to liver microsomes from subjects homozygous for the wild-type UGT1A1*1 allele. This pattern of decreased expression and glucuronidative capacity is consistent with the decreased bilirubin conjugation observed *in vivo* in subjects homozygous for the UGT1A1*28 allele. Together, these results are consistent with previous studies demonstrating significant correlations between UGT genetic variations and tobacco-related cancer risk, suggesting that UGTs may be important targets for future chemoprevention strategies.
