# Supplementary material for: Meeting materials from the 3rd Annual Meeting of the International Society for the Prevention of Tobacco Induced Diseases
Source: Tob Induc Dis. 2004 Dec 15;2(4):168. doi: 10.1186/1617-9625-2-4-168 (PMC2671527; doi:10.1186/1617-9625-2-4-168)
Supplement: Additional file 1 [file 1617-9625-2-4-168-S1.zip › Abstract 6-Actions of chemicals in tobacco products and smoke.pdf]

Abstract 6

Saturday 14.10

Actions of chemicals in tobacco products and smoke

Russell A. Prough, Ph.D.
Department of Biochemistry & Molecular Biology, University of Louisville School of Medicine

Chemicals in tobacco products and smoke play a critical role in the etiology of tobacco-related diseases. Among these are the tobacco-specific nitrosamines, other nicotine derivatives, polycyclic aromatic hydrocarbon (PAH) pyrolysis products, and aldehydic pyrolysis products. Many other compounds exist in tobacco and its pyrolysis products that can have adverse health effects. The presentation will discuss the formation and biological reactions of these two DNA modifying agents, nitrosamines and PAH, which lead to the initiation of cancer. Both sets of compounds require metabolic activation by enzyme systems in our body that lead to the formation of chemically-reactive compounds, which can alkylate DNA. The repair of DNA adducts from these compounds also affect the action of these chemicals in carcinogenesis. Beyond the effects of nicotine as a stimulant, it can undergo nitrosation to form nitrosamines. These compounds, like nicotine itself, can undergo metabolism in our body, predominantly in the liver, to form the _-hydroxylated metabolite, which can alkylate DNA. Not all metabolic steps are deleterious in this process and detoxification can also occur. A similar process occurs for polycyclic aromatic hydrocarbons, in which only a small fraction of the compound is metabolized to a chemical species that can alkylate DNA. Finally, other smoke-derived produces are formed, like acrolein, which also can alter the function of the vascular system, possibly leading to atherosclerosis, cell proliferation, or other inflammatory processes. The spectrum of chemicals and their epigenetic effects will be reviewed in this presentation.
