# Supplementary material for: Meeting materials from the 3rd Annual Meeting of the International Society for the Prevention of Tobacco Induced Diseases
Source: Tob Induc Dis. 2004 Dec 15;2(4):168. doi: 10.1186/1617-9625-2-4-168 (PMC2671527; doi:10.1186/1617-9625-2-4-168)
Supplement: Additional file 1 [file 1617-9625-2-4-168-S1.zip › Abstract 7-Full PDF presentation.pdf]

Reactive oxygen species production from smokeless tobacco

Ernest W.N. Lam, D.M.D., Ph.D., F.R.C.D.(C)

Alberta Heritage Foundation for Medical Research Clinical Investigator
Department of Dentistry
University of Alberta, DPC 2085
Edmonton, AB T6G 2N8

Background

	Incidence	Deaths
Canada[1]	2,926	1,002
U.S.A.[2]	28,260 (est.)	7,230 (est.)

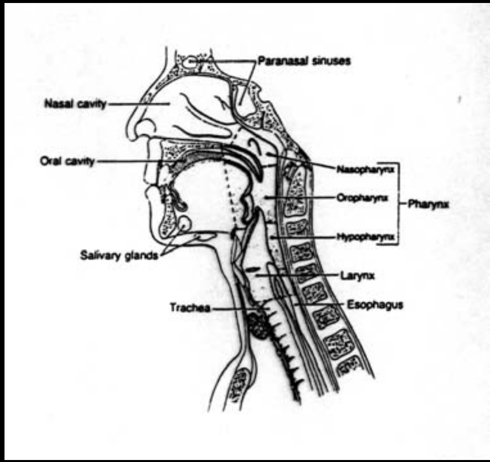

[1]Canadian Cancer Society, 1998
[2]United States National Cancer Institute, 2004
From Vokes *et al.*, *NEJM* **328**:184-194; 1993.

- Free radical
  - *Any species capable of independent existence that contains 1 or more unpaired electrons.*
    - $O_2^{\bullet -}$, $^{\bullet}NO$, $HO^{\bullet}$, $O_2^{\bullet\bullet}$
- Reactive oxygen/nitrogen species
  - *Oxygen or nitrogen-containing molecules that have a higher reactivity than ground state molecular oxygen or nitrogen.*
    - $O_2^{\bullet -}$, $HO^{\bullet}$, H_2O_2, $^1O_2$, $^{\bullet}NO$, $ONOO^-$

Radicals and antioxidants

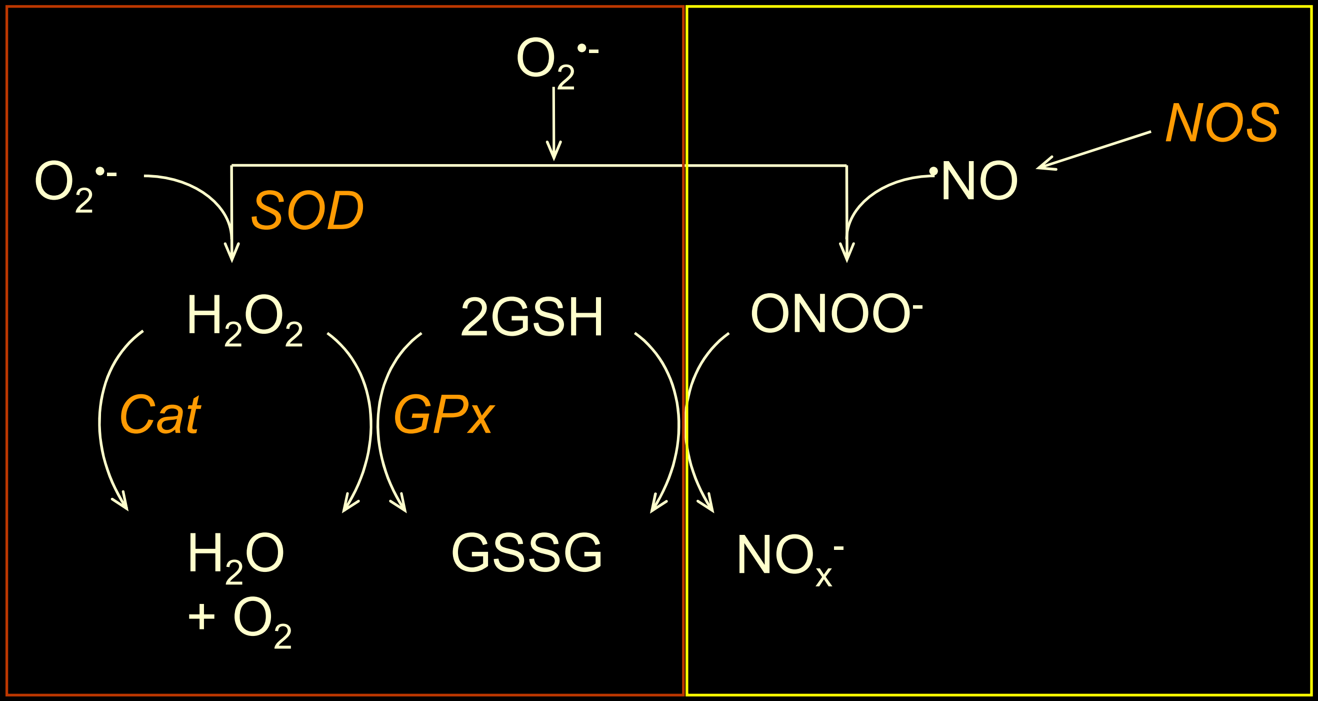

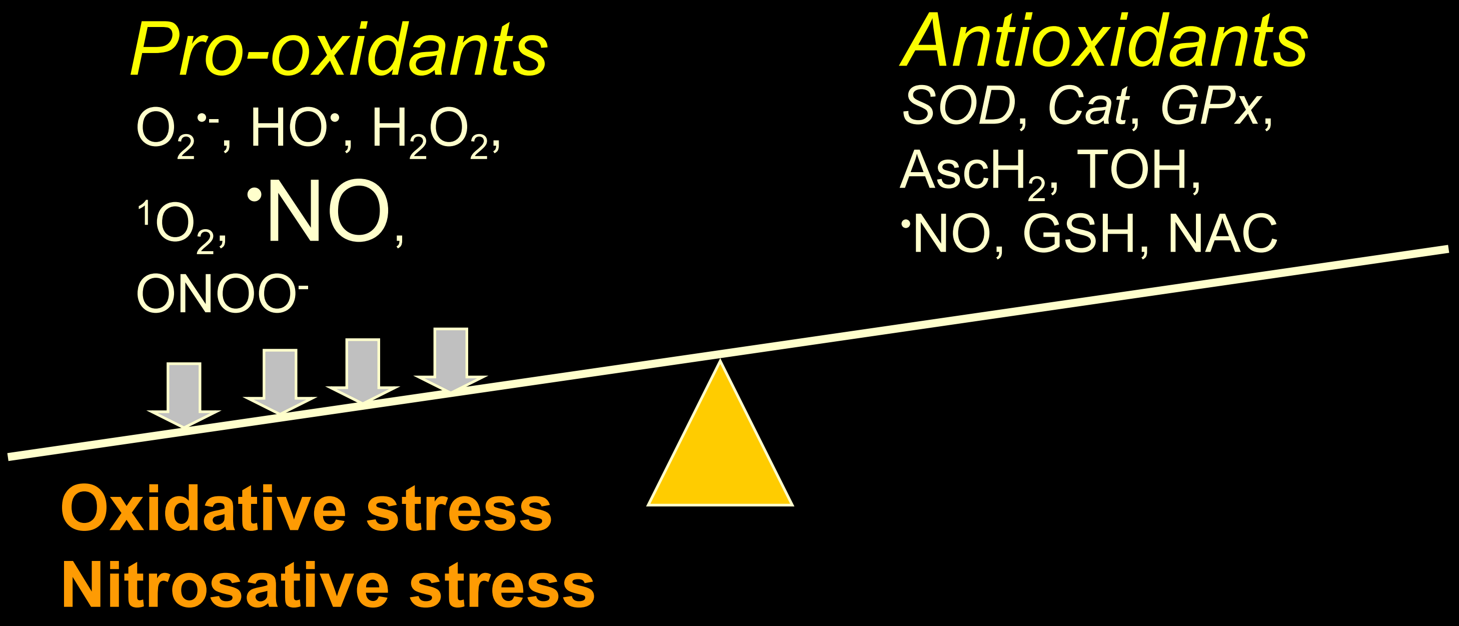
Pro-oxidants
$O_2^{\bullet-}$, $HO^{\bullet}$, H_2O_2,
$^1O_2$, $^{\bullet}NO$,
$ONOO^-$
Antioxidants
SOD, Cat, GPx,
$AscH_2$, TOH,
$^{\bullet}NO$, GSH, NAC
Oxidative stress
Nitrosative stress

- Cellular damage from supra-physiologic levels of $^{\bullet}NO$ are mediated by peroxynitrite ($ONOO^-$), the reaction product of $^{\bullet}NO$ and $O_2^{\bullet -}$.

$$^{\bullet}NO + O_2^{\bullet -} \rightarrow ONOO^-$$

$$\downarrow$$

$$ONOOH \rightarrow {}^{\bullet}NO_2,\ NO_2^+,\ \text{“}HO^{\bullet}\text{”}$$

Smokeless tobacco-related compounds

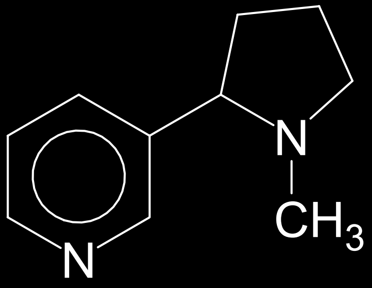

[1]Nicotine

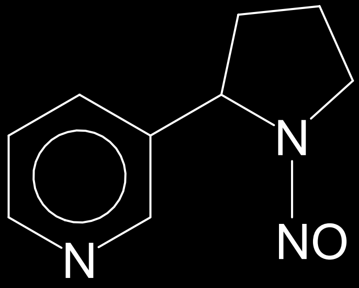

[2]NNN

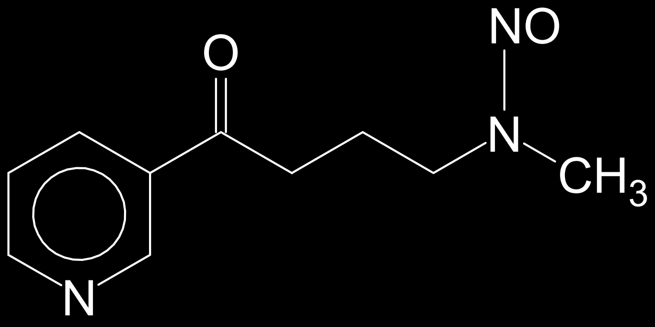

[3]NNK

12.0 ± 0.7 mg/g ST   8.73 ± 1.44 mg/g ST   1.89 ± 0.62 mg/g ST

1Copenhagen brand smokeless tobacco. From Hoffmann D, Djordjevic MV. *Adv Dent Res* **11**:322-339; 1997.

[2]Nitrosonornicotine

[3]4-(N-methyl-N-nitrosamino)-1-(3-pyridyl)-1-butanone

Smokeless tobacco

- 2,7-dichlorofluorescein diacetate fluorescence assay

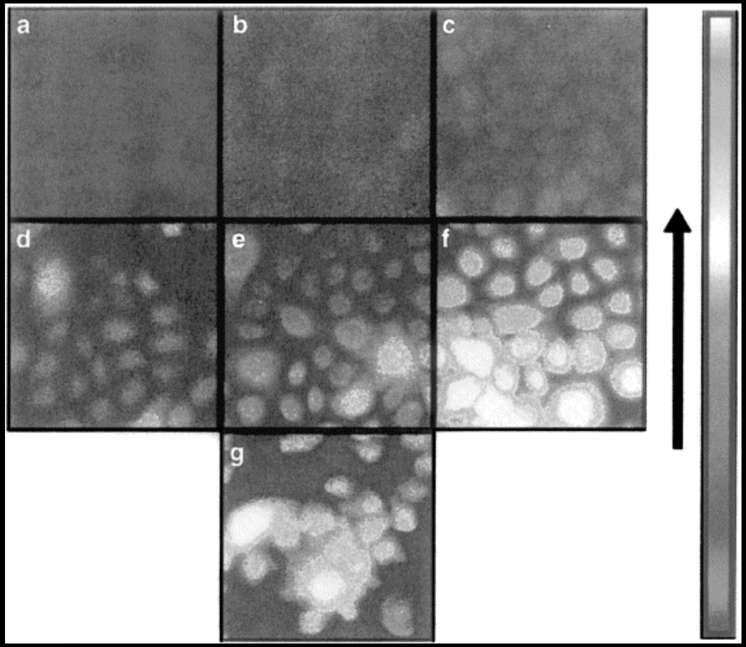

From Bagchi *et al*., *Toxicology* **179**:247-255; 2002.

- Cytochrome c superoxide assay

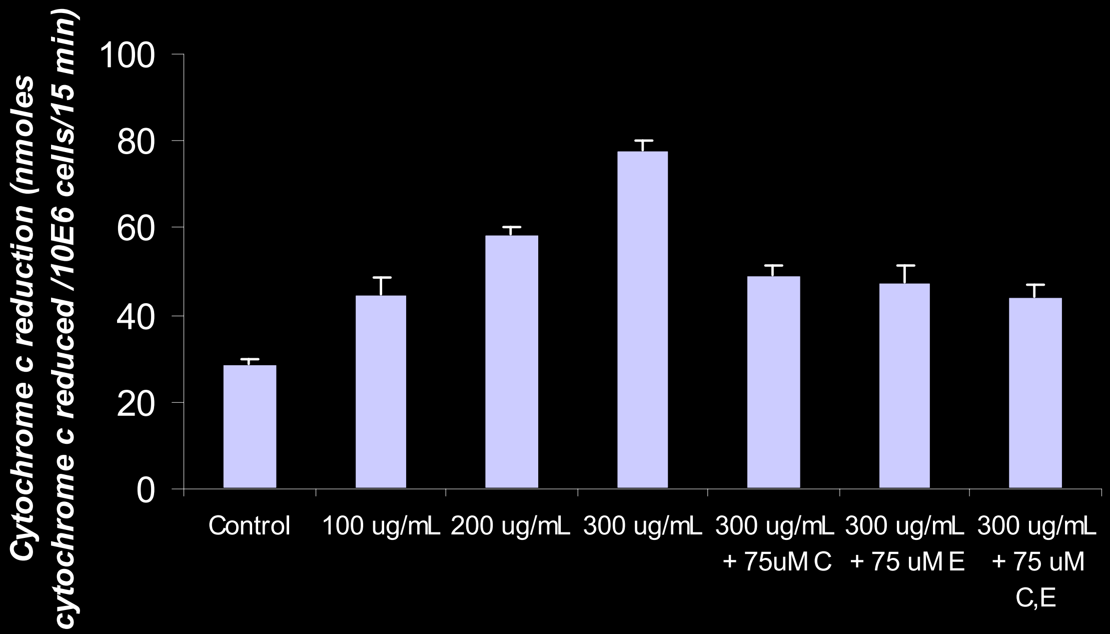

From Bagchi *et al.*, *Free Radic Biol Med* **26**:992-1000; 1999.

- Malondialdehyde (TBARS) lipid peroxidation assay

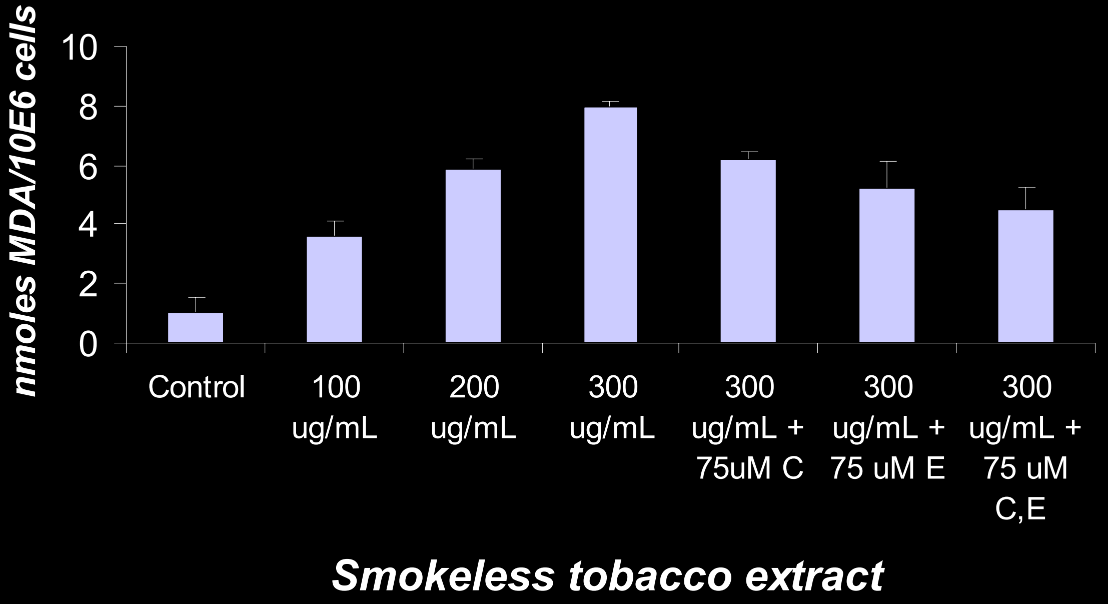

From Bagchi *et al.*, *Free Radic Biol Med* **26**:992-1000; 1999.

- DNA fragmentation assay

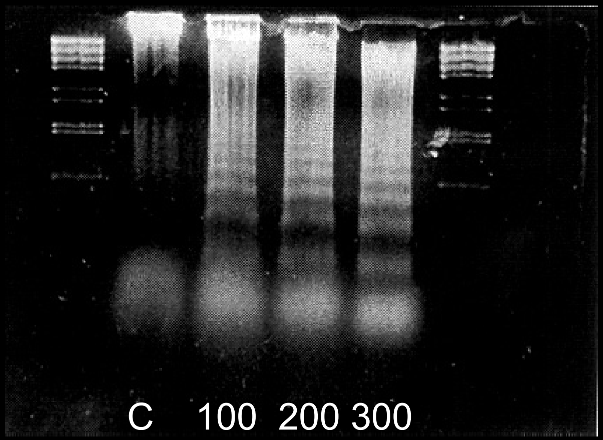

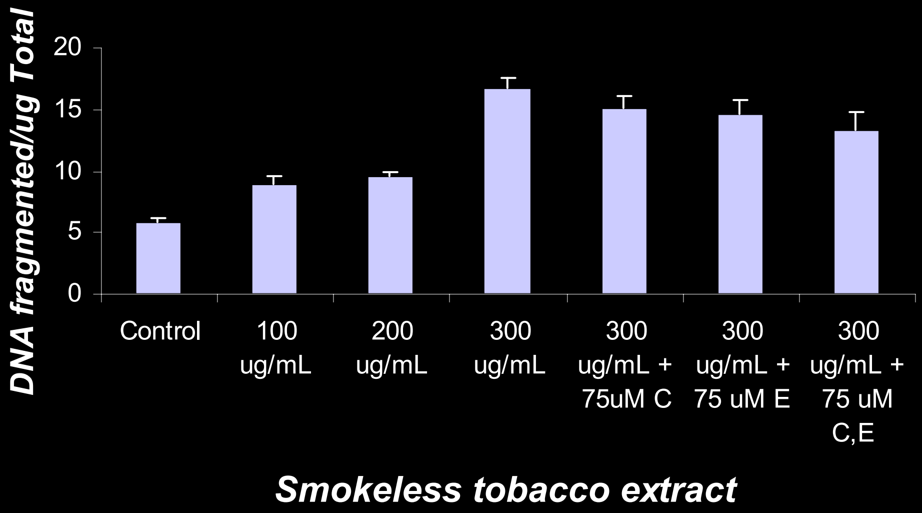

From Bagchi *et al*., *Free Radic Biol Med* **26**:992-1000; 1999.

Nicotine

- Luminol chemiluminescence assay

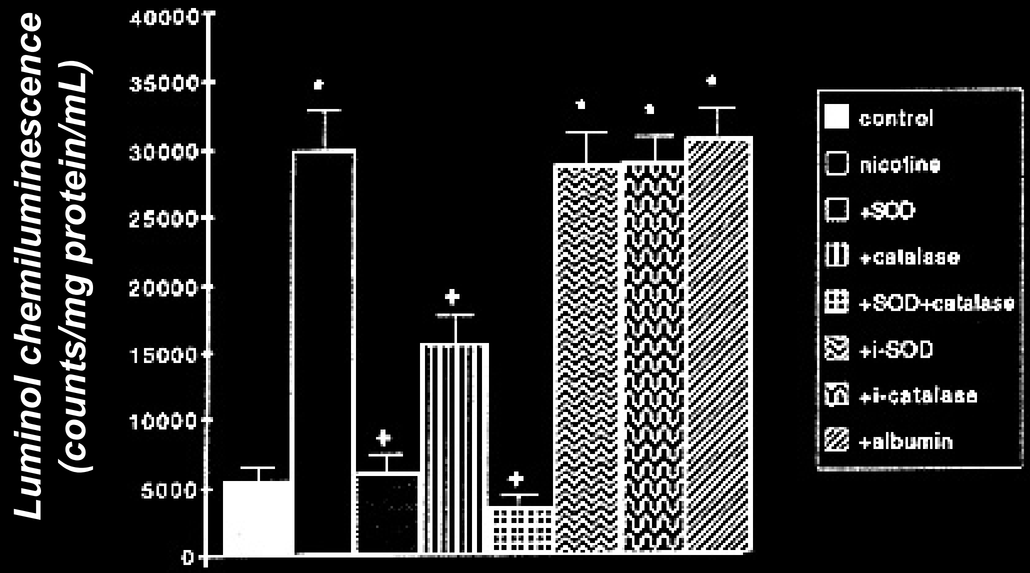

From Wetscher *et al.*, *Dig Dis Sci* **40**:853-858; 1995.

- Malondialdehyde (TBARS) lipid peroxidation assay

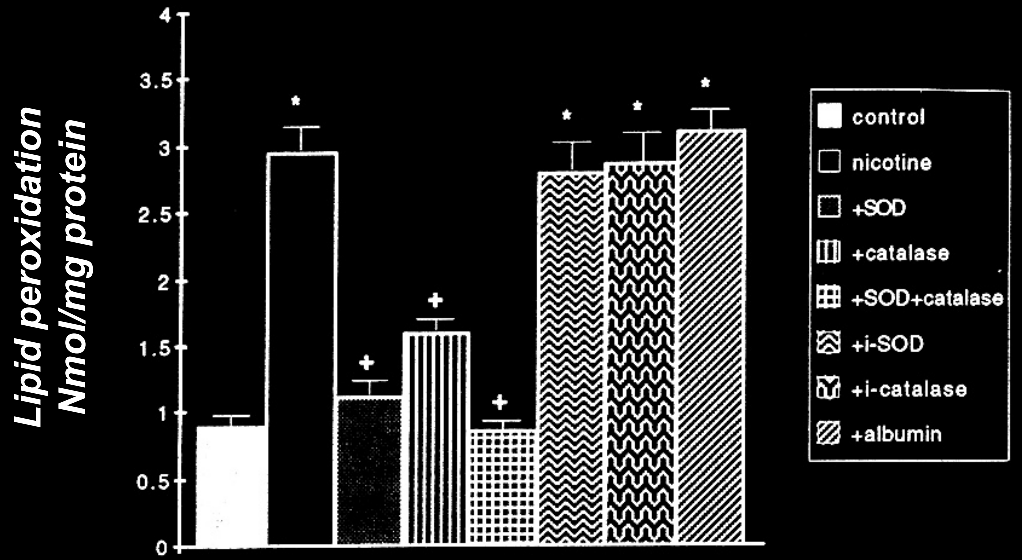

From Wetscher *et al.*, *Dig Dis Sci* **40**:853-858; 1995.

NNN/NNK

- Fluorometric DNA unwinding assay

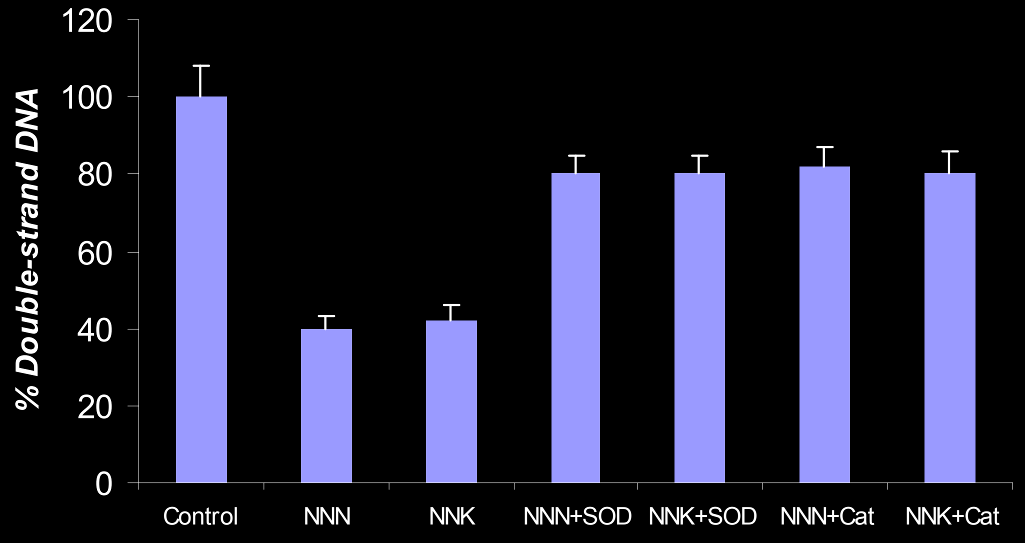

From Weitberg and Corvese, *Clin Genet* **43**:88-91; 1993.

- Can tobacco-related compounds release radicals other than $O_2^{\bullet-}$ or $HO^{\bullet}$ radical?

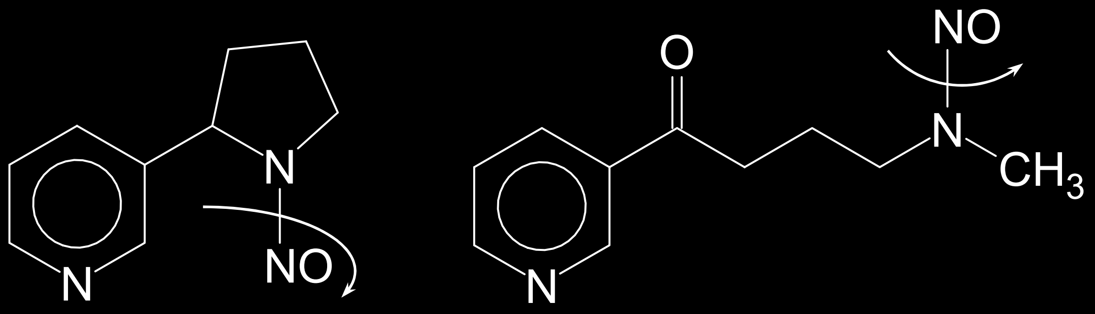

NNN NNK

- Electron paramagnetic resonance spin trapping

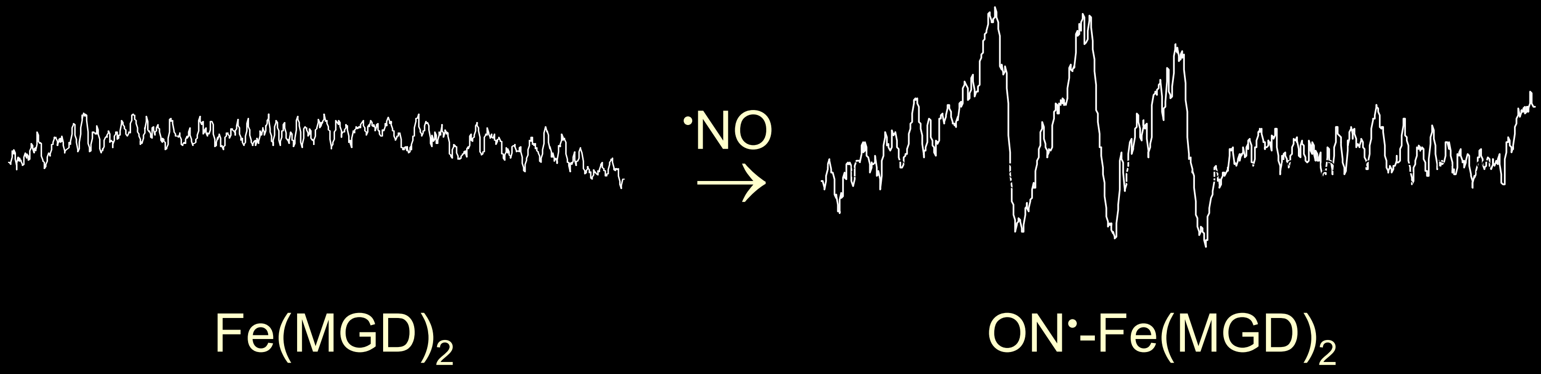

Lam *et al*., *Tobac Ind Dis* **1**:207-211; 2003.

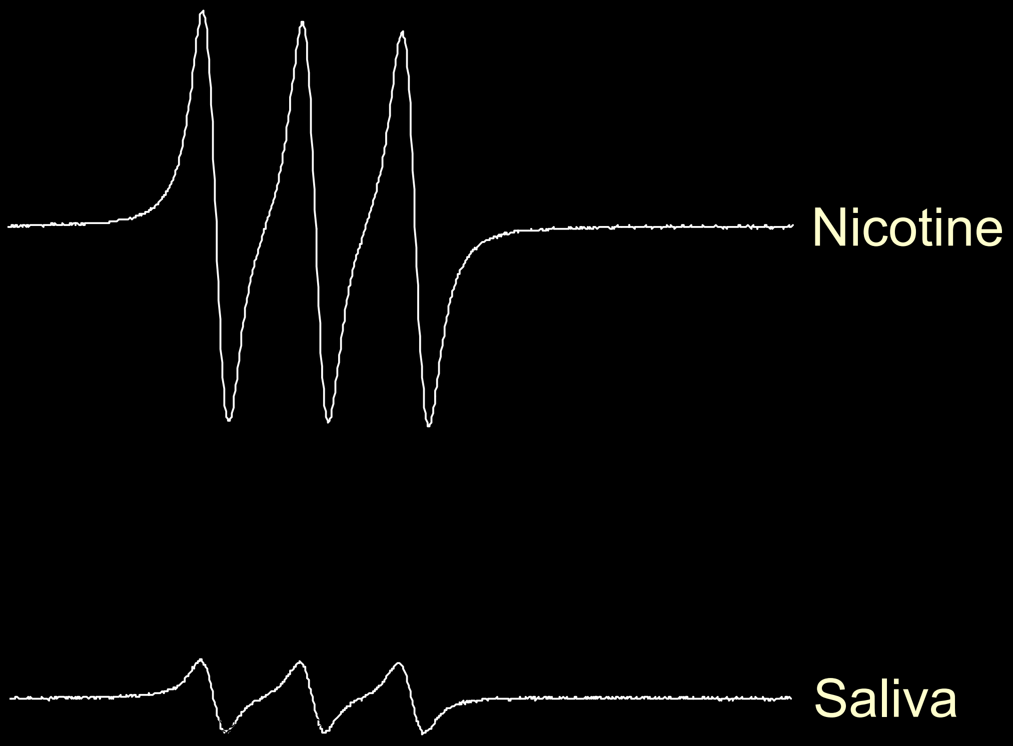

Lam *et al*., *Tobac Ind Dis* **1**:207-211; 2003.

- Chemiluminescent $^{\bullet}NO$ detection

	in PBS (pH 7.4)	in human saliva
**PBS**	5 ± 1 mM	-
**Human saliva**	-	38 ± 17 mM
**ST**	1100 ± 50 mM<br>2.53 ± 0.10 mmol/g ST	1380 ± 80 mM<br>2.76 ± 0.16 mmol/g ST
**Nicotine**	< 0.02 mM	150 ± 12 mM<br>2.81 ± 0.23 nmol/mg nicotine
**NNN**	< 0.02 mM	121 ± 6 mM<br>5.90 ± 0.30 nmol/mg NNN
**NNK**	< 0.02 mM	113 ± 5 mM<br>5.45 ± 0.25 nmol/mg NNK

Lam *et al.*, *Tobac Ind Dis* **1**:207-211; 2003.

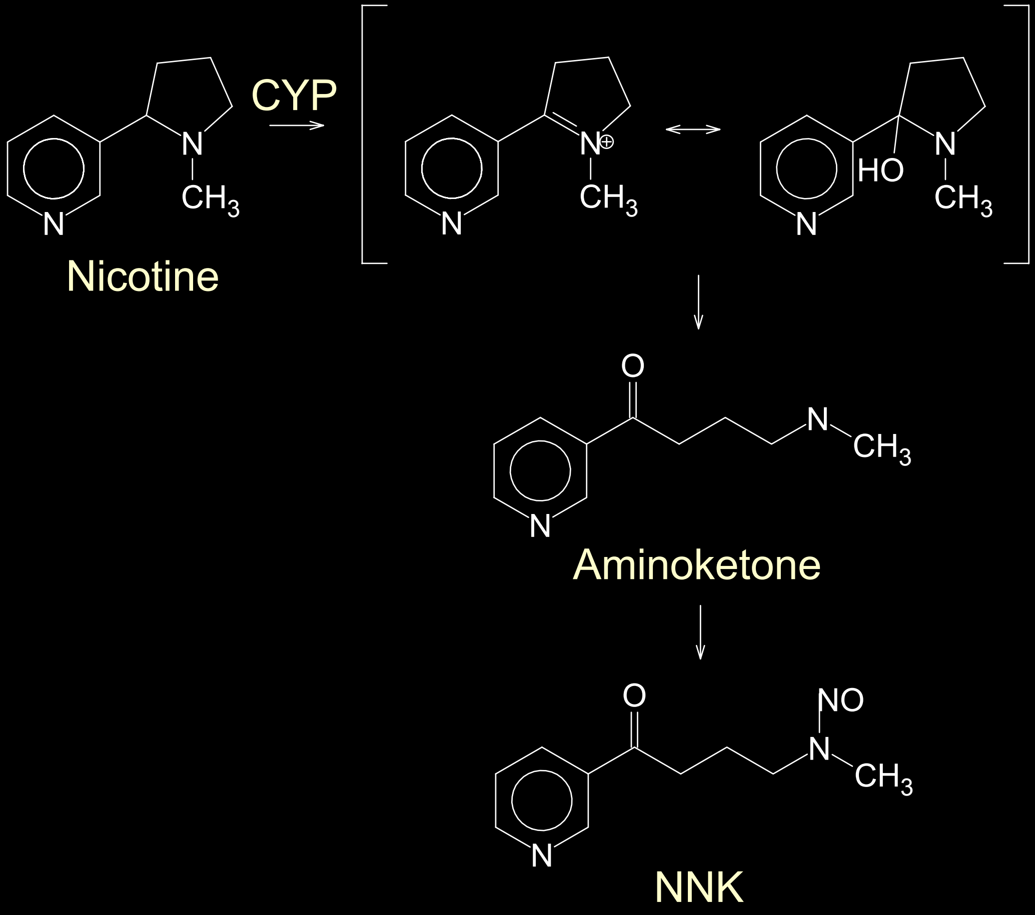

Hecht *et al.*, *PNAS* **97**:12493-12497; 2000.

- Comet assay
  - POII cells treated with 12 mM nicotine

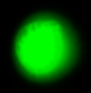

Control

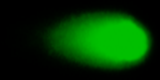

2min

$$\text{Tail moment} = \text{tail length} \times \%\ \text{tail DNA} = \text{tail length} \times \frac{(\text{total intensity} - \text{head intensity})}{\text{total intensity}}$$

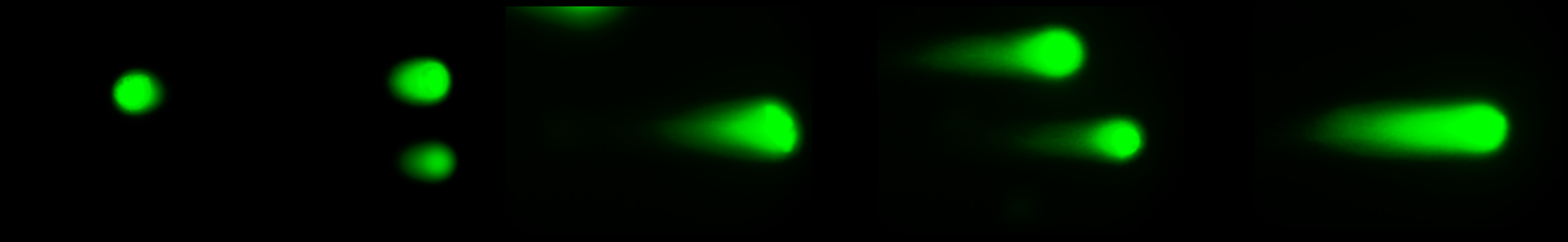

Control 3% ST 6% ST 12% ST 25% ST

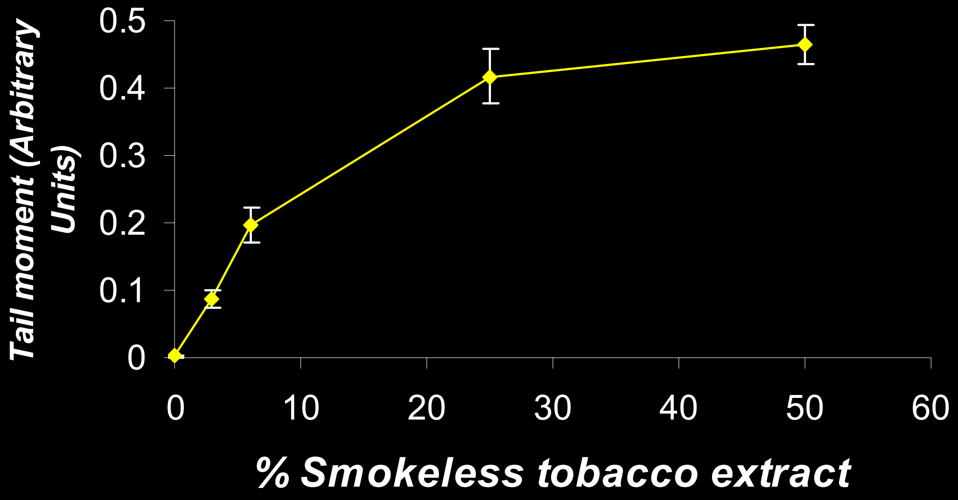

Barley *et al*., *J. Dent. Res.* **83**:903-908*;* 2004.

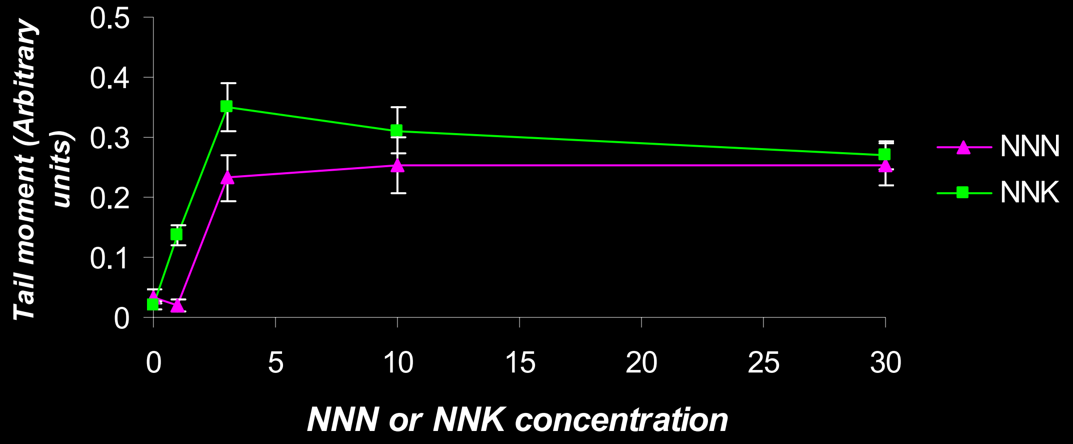
0.5
0.4
0.3
0.2
0.1
0
Tail moment (Arbitrary units)
0
5
10
15
20
25
30
NNN or NNK concentration
NNN
NNK

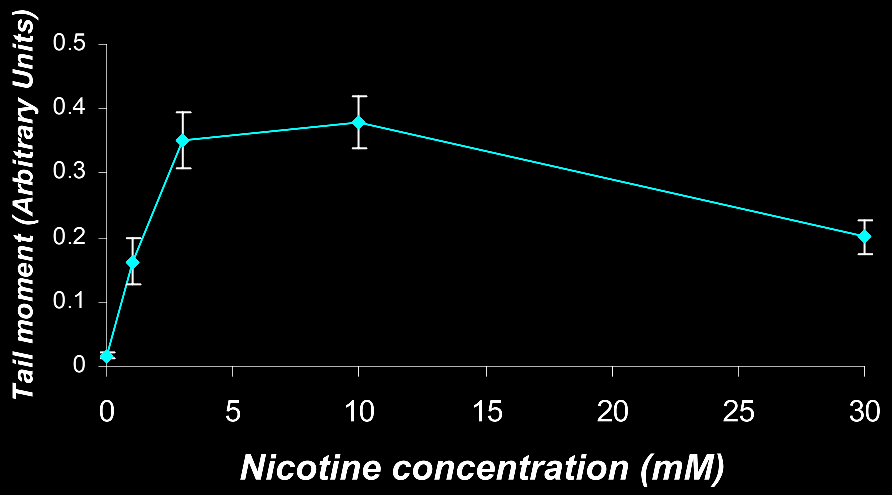
0.5
0.4
0.3
0.2
0.1
0
Tail moment (Arbitrary Units)
0
5
10
15
20
25
30
Nicotine concentration (mM)

- Hamster cheek pouch tissues treated with smokeless tobacco and smokeless tobacco-related compounds

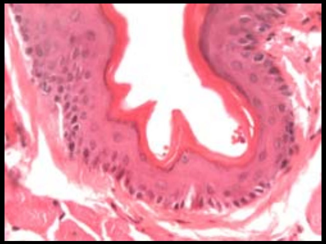

Verruciform pattern

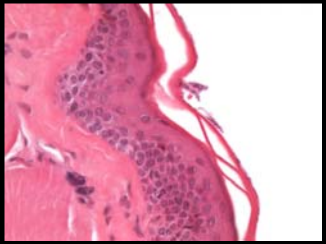

Increased cellularity

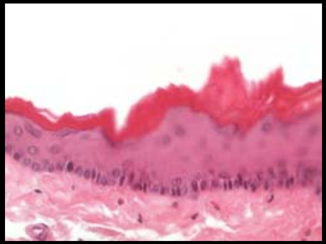

Hyperchromatism

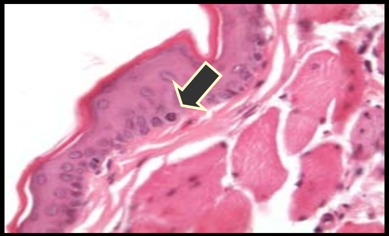

Mitotic figures

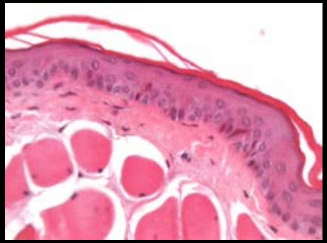

Pleomorphism

Barley *et al*., *J. Dent. Res.* **83**:903-908*;* 2004.

- Ischiropoulous (*Archs. Biochem. Biophys.* **356**:1-11; 1998) suggests that of the major putative mechanisms of macromolecular nitrosation, one of the most plausible is the uncatalyzed nitrosation of macromolecules by $ONOO^-$.

- 3-Nitrotyrosine (3-NT) immunohistochemistry

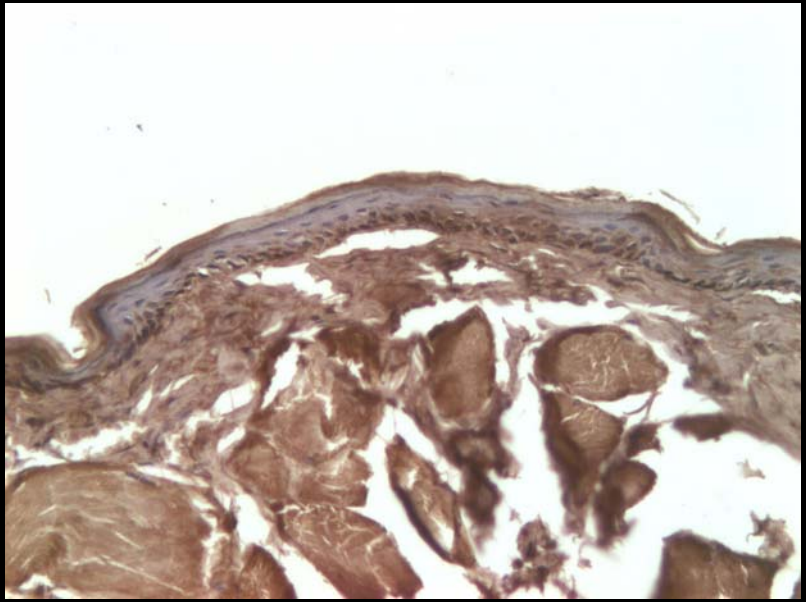

Control

Barley *et al.*, *J. Dent. Res.* **83**:903-908*;* 2004.

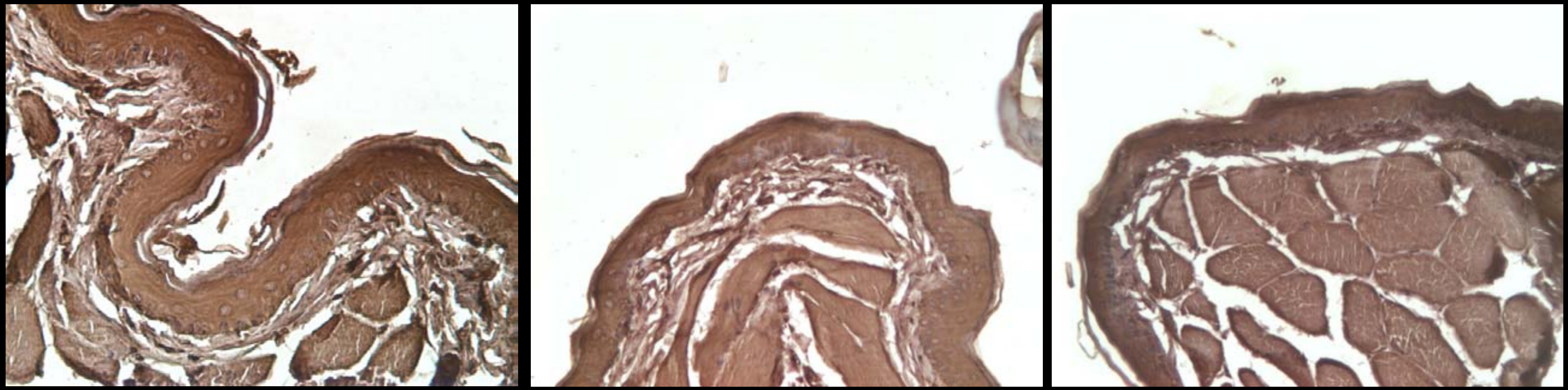

Smokeless tobacco NNN NNK

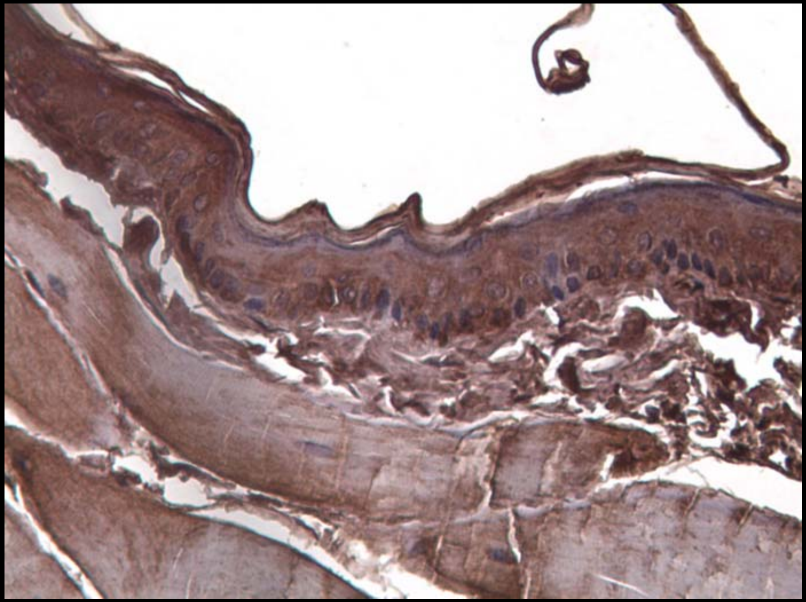

Nicotine

- Semi-quantitative 3-NT immunohistochemistry

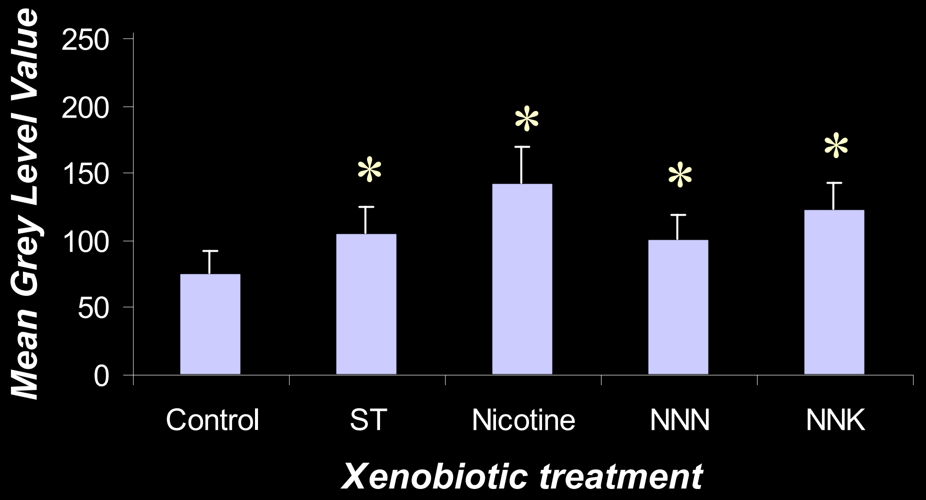

* p<0.005

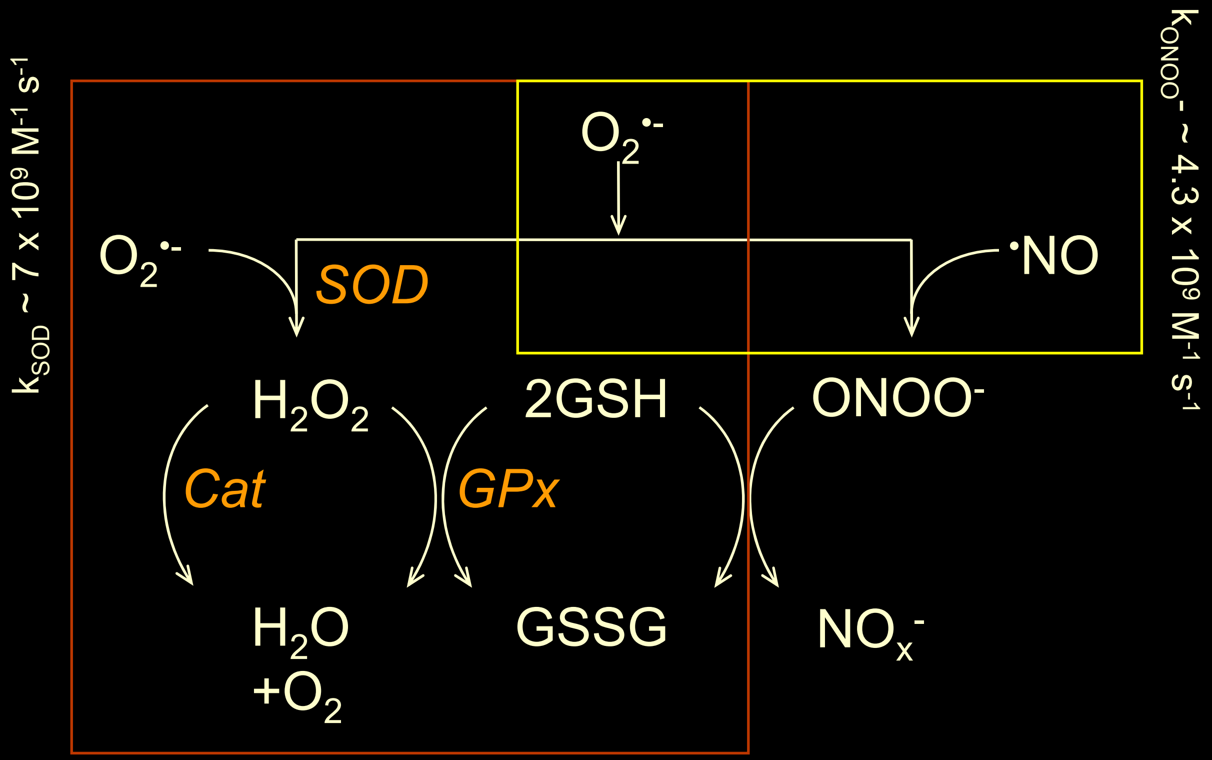

k_{SOD} ~ 7 x 10^9 M^{-1} s^{-1}
$O_2^{\bullet -}$
$O_2^{\bullet -}$
SOD
$^{\bullet}NO$
k_{ONOO^-} ~ 4.3 x 10^9 M^{-1} s^{-1}
H_2O_2
2GSH
$ONOO^-$
Cat
GPx
H_2O
$+O_2$
GSSG
NO_x^-

- *SOD1* transgene overexpression

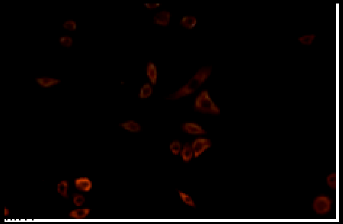

HBEC-1

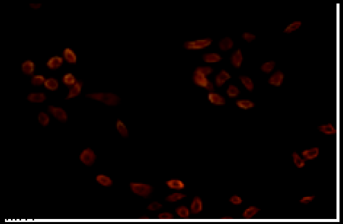

200 MOI Ad5

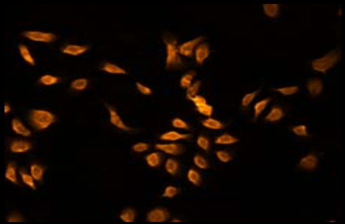

200 MOI Ad*SOD1*

CuZnSOD →

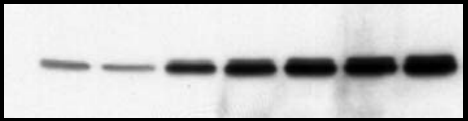

C Ad5 50 100 200 400 800 MOI

CuZnSOD →

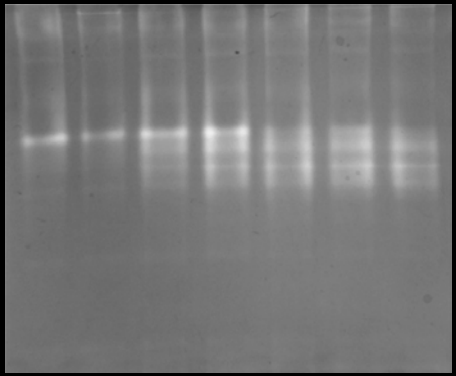

C Ad5 50 100 200 400 800 MOI

- *SOD1* protects against nicotine toxicity

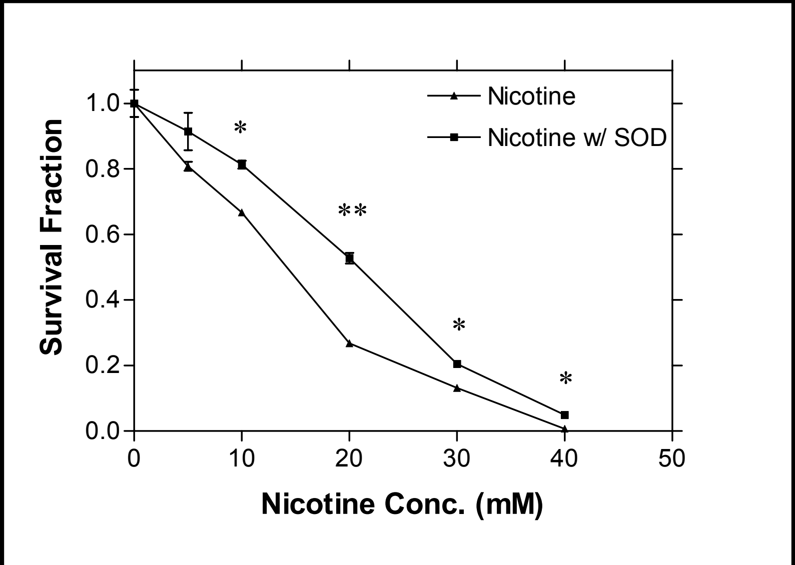

SOD1 protects against DNA SSBs from nicotine

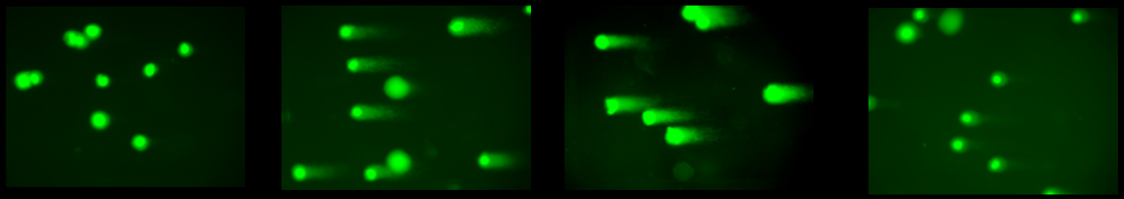

Control HBEC-1 200 MOI Ad5 200 MOI Ad*SOD1*

+ 30 mM nicotine

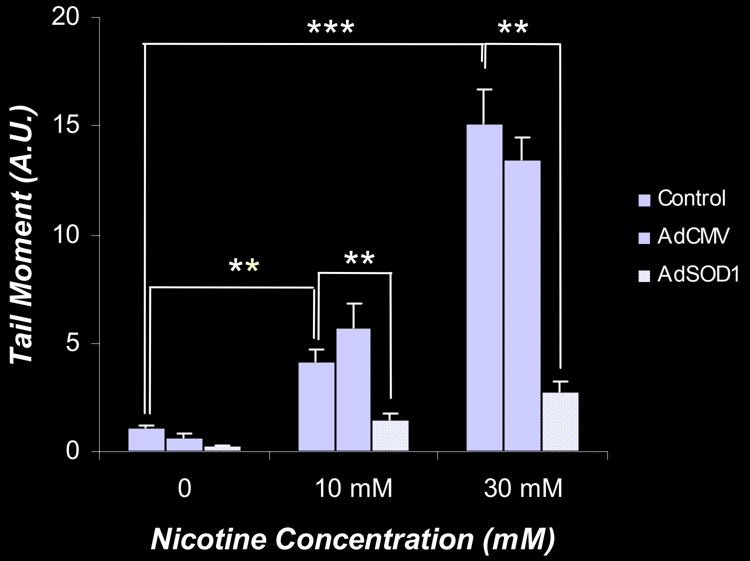

- *SOD1* inhibits 3-NT immunoreactivity

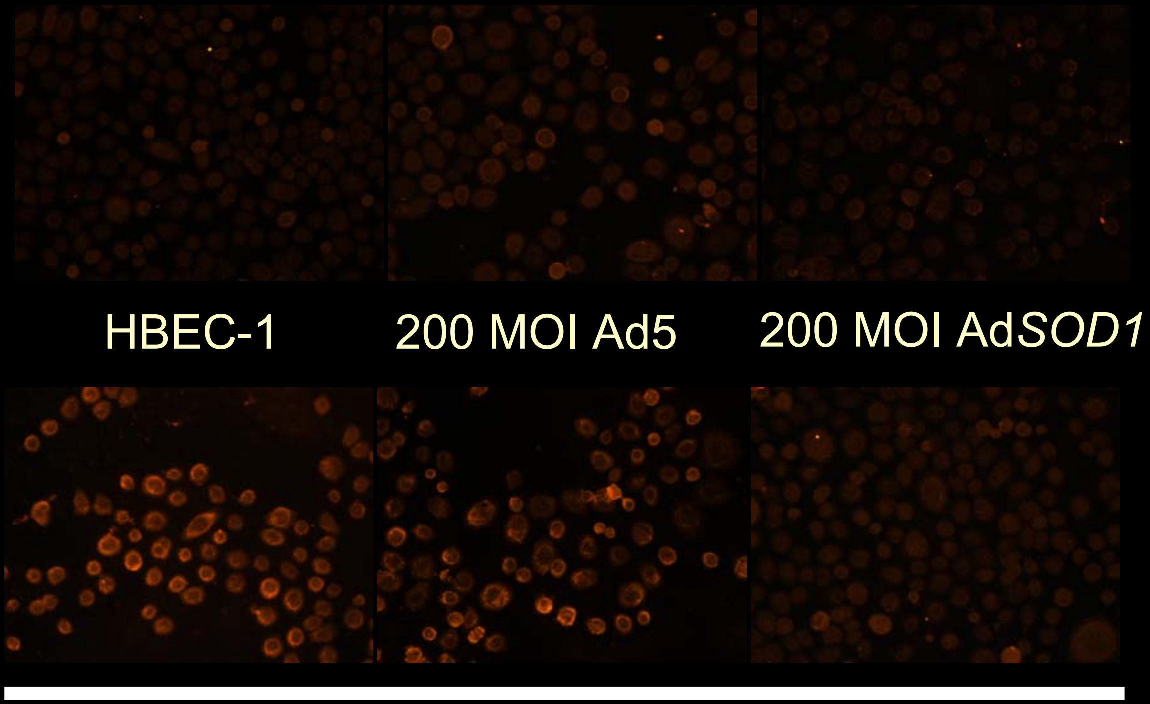

+ 10 mM nicotine

Acknowledgements

- University of Alberta
  - Dentistry
    - R.D.C. Barley, M.Sc.
    - S. Pollock
    - M.C. Shallow, M.Sc.
    - E. Peters, D.D.S., F.R.C.D.(C)
  - Pediatrics and Pharmacology
    - R. Schulz, Ph.D.
- The University of Iowa
  - College of Medicine
    - G.R. Buettner, Ph.D.
    - E.E. Kelley, Ph.D.
    - S.M. Martin, B.S.
- Medical College of Georgia
  - G.S. Schuster, D.D.S., Ph.D.
- Canadian Institutes of Health Research
- Alberta Heritage Foundation for Medical Research
