# Supplementary material for: Meeting materials from the 3rd Annual Meeting of the International Society for the Prevention of Tobacco Induced Diseases
Source: Tob Induc Dis. 2004 Dec 15;2(4):168. doi: 10.1186/1617-9625-2-4-168 (PMC2671527; doi:10.1186/1617-9625-2-4-168)
Supplement: Additional file 1 [file 1617-9625-2-4-168-S1.zip › Abstract 7-Reaction oxygen species production from smokeless tobacco.pdf]

Abstract 7

Saturday 14.40

Reaction oxygen species production from smokeless tobacco

Ernest W.N. Lam
Departments of Dentistry and Oncology, University of Alberta, Edmonton, Canada

A number of well-known xenobiotics exert their pharmacologic effects via the generation of reactive oxygen species (ROS), oxygen-containing molecules that have a higher reactivity than ground state molecular oxygen. One ROS species, superoxide ($O_2^{\bullet -}$), can react with the nitric oxide ($^{\bullet}NO$) free radical to form the powerful biological nitrosating agent, peroxynitrite ($ONOO^-$). Unchecked, ROS and $ONOO^-$ may damage vital cellular macromolecules, including DNA. To protect against this damage, cells have developed a compartmentalized system of antioxidant enzymes that control ROS levels, and by extension, $ONOO^-$ formation, at or near their sites of production. These include the superoxide dismutase (SOD), catalase and glutathione peroxidase (GPx) enzymes. We and others have shown that smokeless tobacco and some of its constituents including nicotine, nitrosonornicotine (NNN) and 4-(methyl-N-nitrosamino)-1-(3-pyridyl)-1-butanone (NNK) have the capacity to induce or directly release ROS and $^{\bullet}NO$ at levels that cause lipid peroxidation, protein tyrosine nitrosation and DNA single strand breaks in cells and tissue explants. Moreover, incubation of these experimental systems with recombinant antioxidant enzymes, or gene transfer of antioxidant enzymes protects cells against this damage. Understanding the mechanisms of smokeless tobacco damage to oral cells and tissues could provide the basis for the development of novel strategies that could prevent, or potentially reverse epithelial changes in the oral mucosa from smokeless tobacco. This work was supported by grants from the Alberta Heritage Foundation for Medical Research and the Canadian Institutes for Health Research.
