# Supplementary material for: Meeting materials from the 3rd Annual Meeting of the International Society for the Prevention of Tobacco Induced Diseases
Source: Tob Induc Dis. 2004 Dec 15;2(4):168. doi: 10.1186/1617-9625-2-4-168 (PMC2671527; doi:10.1186/1617-9625-2-4-168)
Supplement: Additional file 1 [file 1617-9625-2-4-168-S1.zip › Abstract 8-Cytoxic and mutagenic effects of tobacco-borne free fatty acids.pdf]

Abstract 8

Saturday 15.10

Cytoxic and mutagenic effects of tobacco-borne free fatty acids

Xueshan Gao, Mingwei Qian, John W. Eaton and W. Glenn McGregor
J. G. Brown Cancer Center and Departments of Medicine and Pharmacology/Toxicology
University of Louisville, Louisville, KY 40202

We previously reported that tobacco smoke contains substances capable of binding iron in an aqueous medium and transferring the metal into organic solvents and intact mammalian red cells. Most, if not all of this iron binding activity was found to be due to free fatty acids (FFAs) which are abundant in tobacco smoke. Furthermore, micromolar amounts of these same free fatty acids promote phagocyte:endothelial interactions and neutrophil oxidative killing of epithelial cells. These earlier observations suggested that delocalization of iron within the lung by smoke-borne FFAs might contribute to both the chronic pulmonary inflamation and carcinogenesis associated with smoking. We have therefore investigated the cytotoxic and mutagenic effects of micromolar amounts of FFAs and iron on cultured human lung fibroblasts (immortalized by expression of telomerase). The results indicate that neither iron nor FFA alone is cytotoxic in low concentrations. However, when combined, the same low concentrations of iron and FFA exert synergistic toxicity. Furthermore, the combination of FFA and iron is highly mutagenic, inducing almost as many selectable mutations in the *HGPRT* gene as benzo[a]pyrene diolepoxide, a class I carcinogen present in cigarette smoke. The combination also promotes transformation of NIH 3T3 cells into an anchorage-independent phenotype. These results indicate that FFAs in tobacco smoke may be important contributors to both the pulmonary inflammation and carcinogenesis associated with smoking.
