# Supplementary material for: Meeting materials from the 3rd Annual Meeting of the International Society for the Prevention of Tobacco Induced Diseases
Source: Tob Induc Dis. 2004 Dec 15;2(4):168. doi: 10.1186/1617-9625-2-4-168 (PMC2671527; doi:10.1186/1617-9625-2-4-168)
Supplement: Additional file 1 [file 1617-9625-2-4-168-S1.zip › Abstract 9-Full PDF presentation.pdf]

New Treatments for Lung Cancer

Donald M. Miller, M.D., Ph.D.

10/30/04

Change in the US Death Rates* by Cause, 1950 & 2001

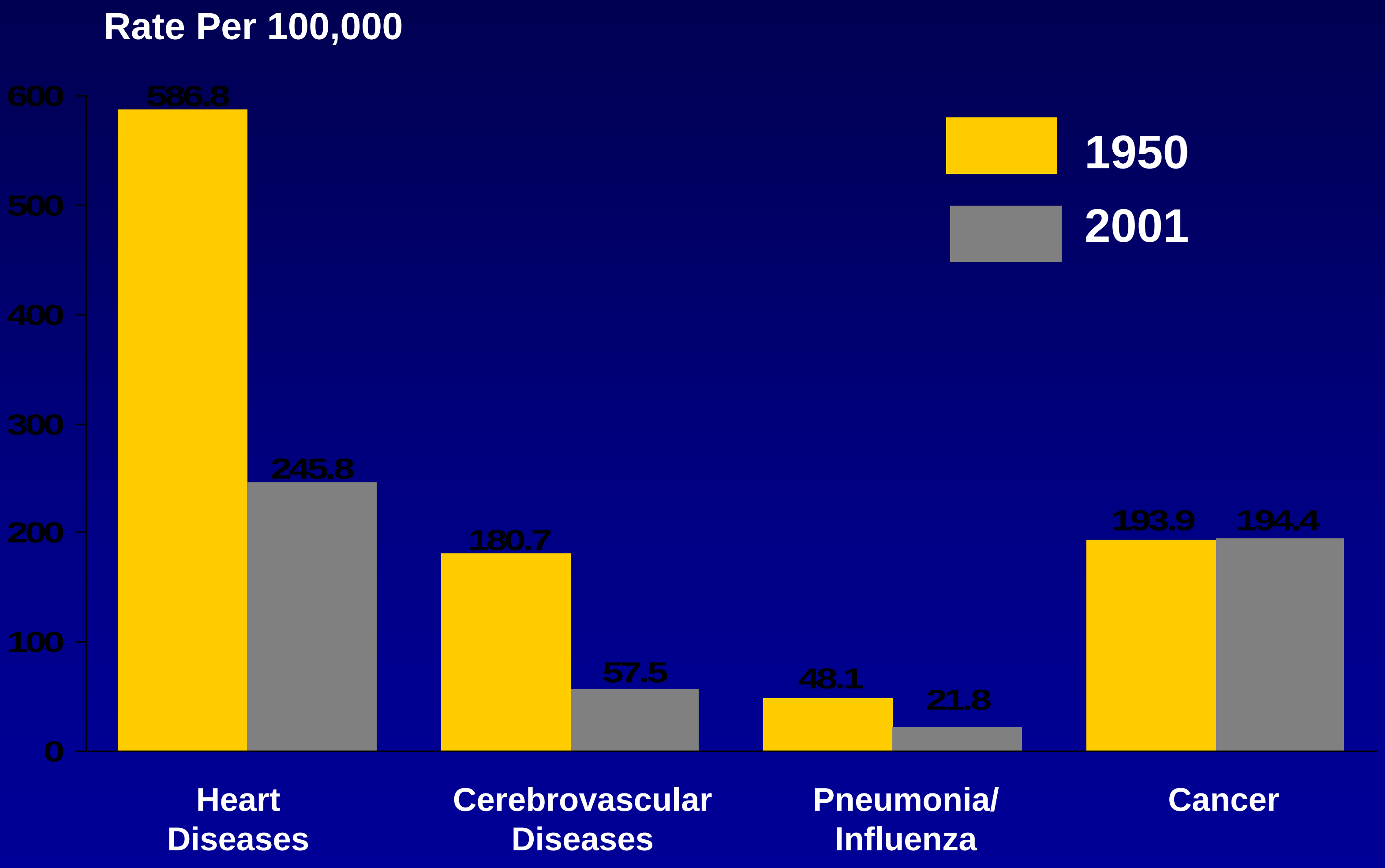

* Age-adjusted to 2000 US standard population.
Sources: 1950 Mortality Data - CDC/NCHS, NVSS, Mortality Revised.
2001 Mortality Data–NVSR-Death Final Data 2001–Volume 52, No. 3.
http://www.cdc.gov/nchs/data/nvsr/nvsr52/nvsr52_03.pdf

Cancer Death Rates*, for Men, US, 1930-2000

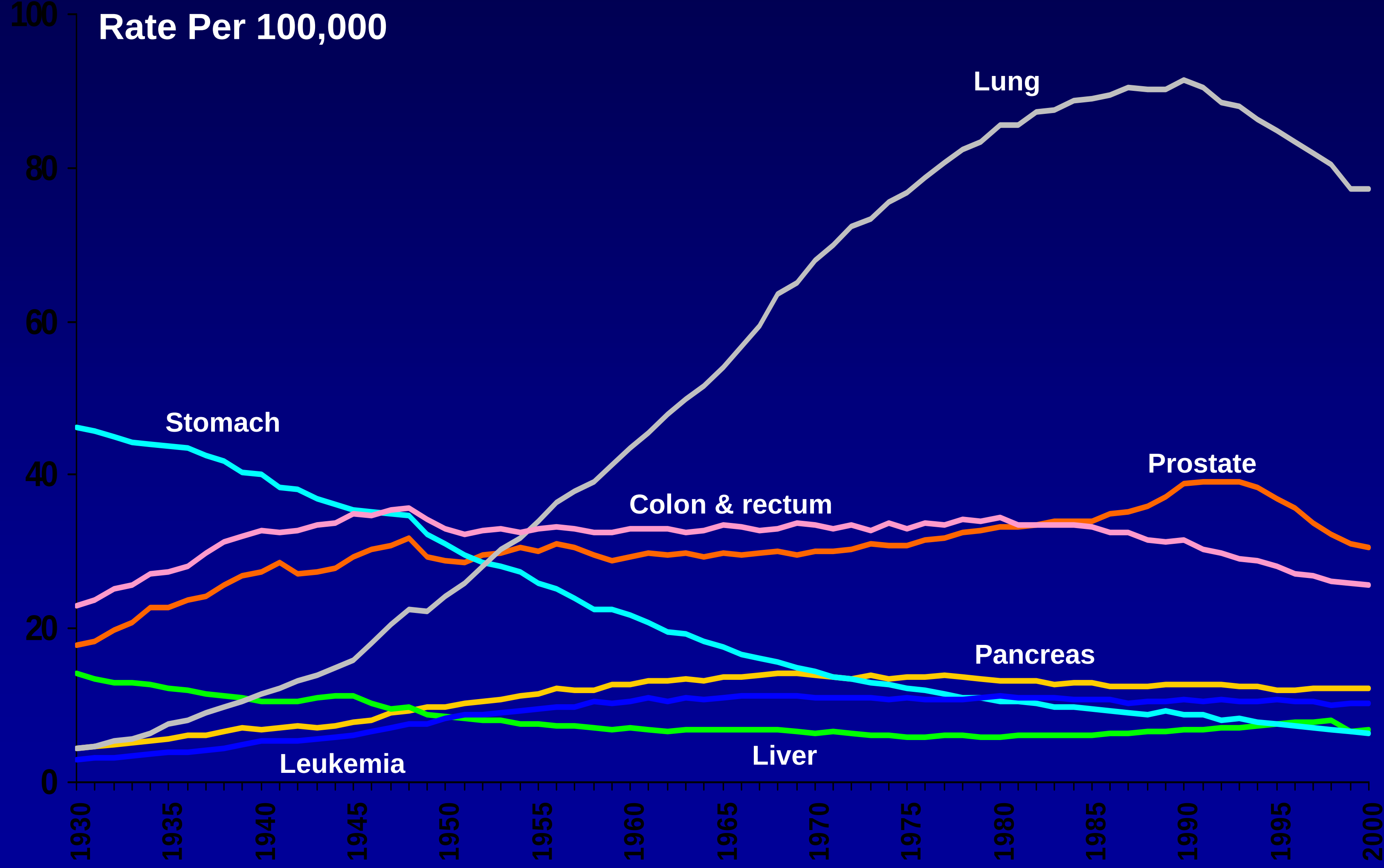

*Age-adjusted to the 2000 US standard population.
Source: US Mortality Public Use Data Tapes 1960-2000, US Mortality Volumes 1930-1959, National Center for Health Statistics, Centers for Disease Control and Prevention, 2003.

Cancer Death Rates*, for Women, US, 1930-2000

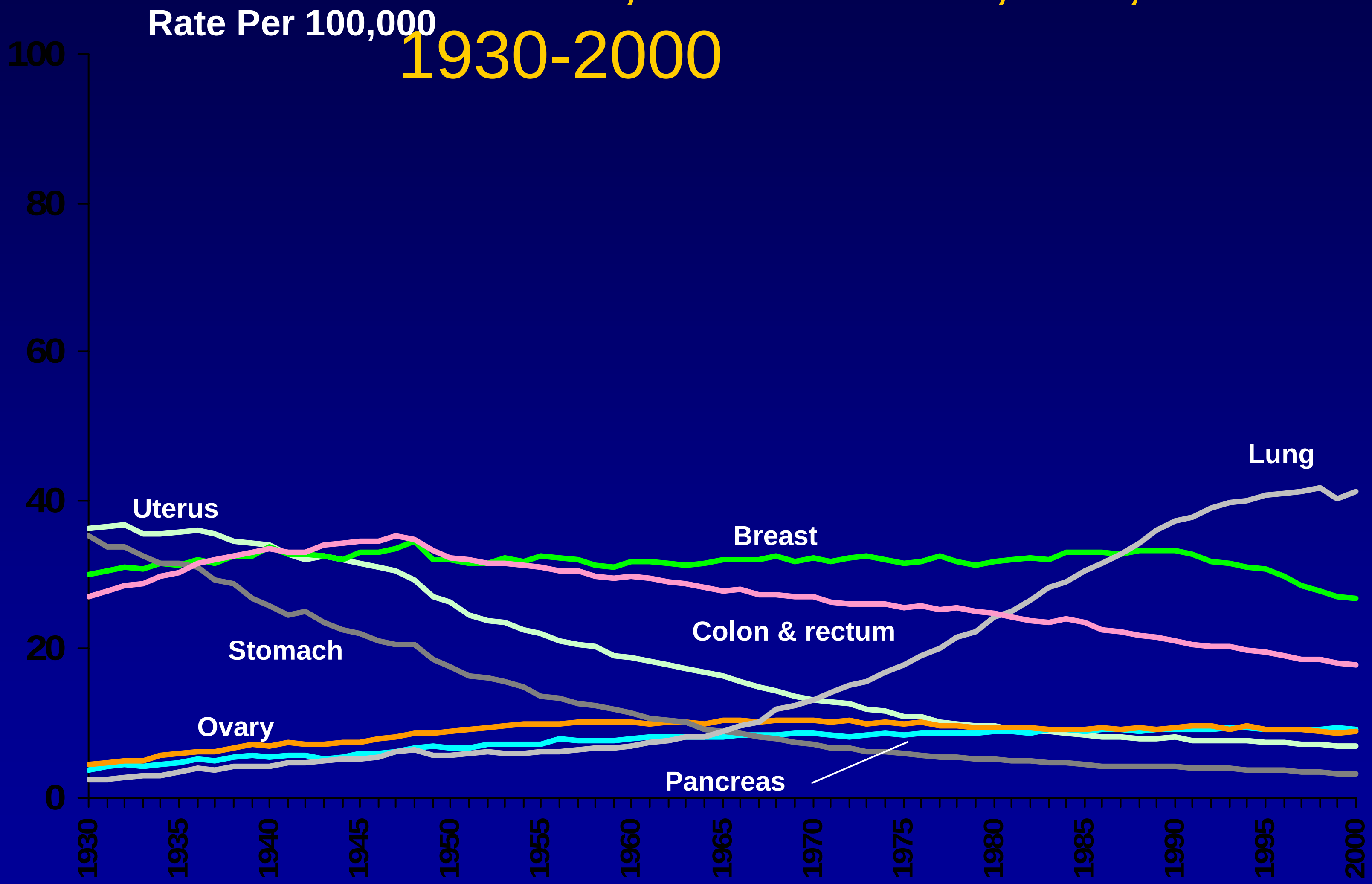

*Age-adjusted to the 2000 US standard population.
Source: US Mortality Public Use Data Tapes 1960-2000, US Mortality Volumes 1930-1959, National Center for Health Statistics, Centers for Disease Control and Prevention, 2003.

2004 Estimated US Cancer Deaths*

Men
290,890

Lung & bronchus	32%
Prostate	10%
Colon & rectum	10%
Pancreas	5%
Leukemia	5%
Non-Hodgkin lymphoma	4%
Esophagus	4%
Liver & intrahepatic bile duct	3%
Urinary bladder	3%
Kidney	3%
All other sites	21%

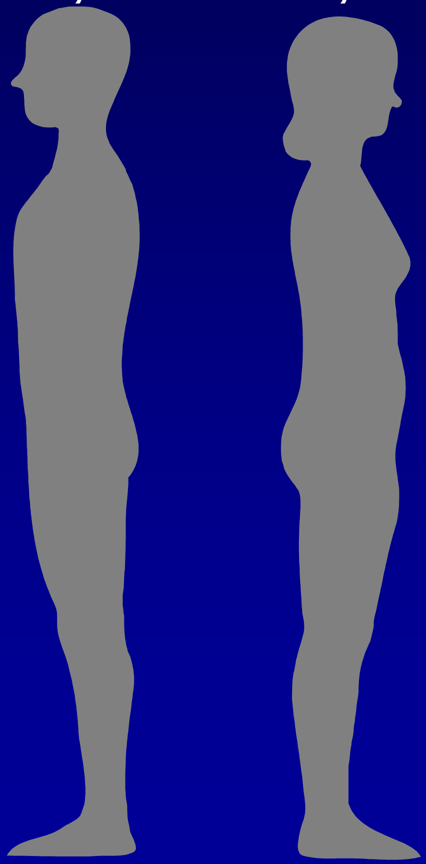

Women
272,810

25%	Lung & bronchus
15%	Breast
10%	Colon & rectum
6%	Ovary
6%	Pancreas
4%	Leukemia
3%	Non-Hodgkin lymphoma
3%	Uterine corpus
2%	Multiple myeloma
2%	Brain/ONS
24%	All other sites

ONS=Other nervous system.
Source: American Cancer Society, 2004.

2004 Estimated US Cancer Cases*

Men
699,560

Prostate	33%
Lung & bronchus	13%
Colon & rectum	11%
Urinary bladder	6%
Melanoma of skin	4%
Non-Hodgkin lymphoma	4%
Kidney	3%
Oral Cavity	3%
Leukemia	3%
Pancreas	2%
All Other Sites	18%

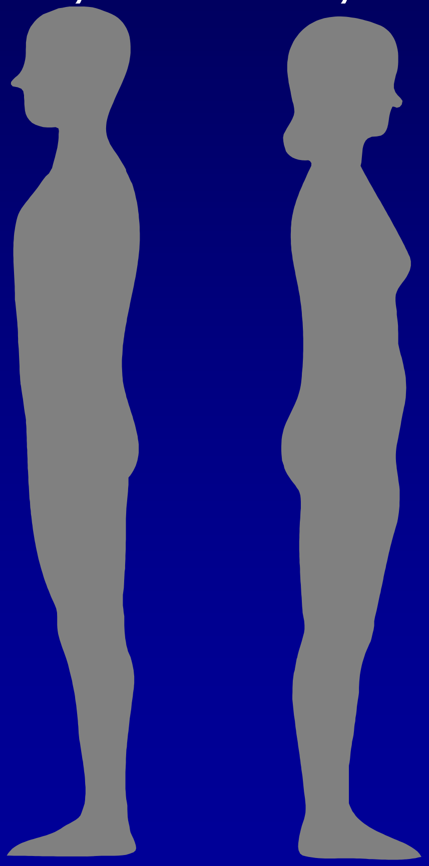

Women
668,470

•32%	Breast
•12%	Lung & bronchus
•11%	Colon & rectum
•6%	Uterine corpus
• 4%	Ovary
• 4%	Non-Hodgkin lymphoma
•4%	Melanoma of skin
•3%	Thyroid
•2%	Pancreas
•2%	Urinary bladder
•20%	All Other Sites

***Excludes basal and squamous cell skin cancers and in situ carcinomas except urinary bladder.**
Source: American Cancer Society, 2004.

Cancer Death Rates*, All Sites Combined, All Races, US, 1975-2000

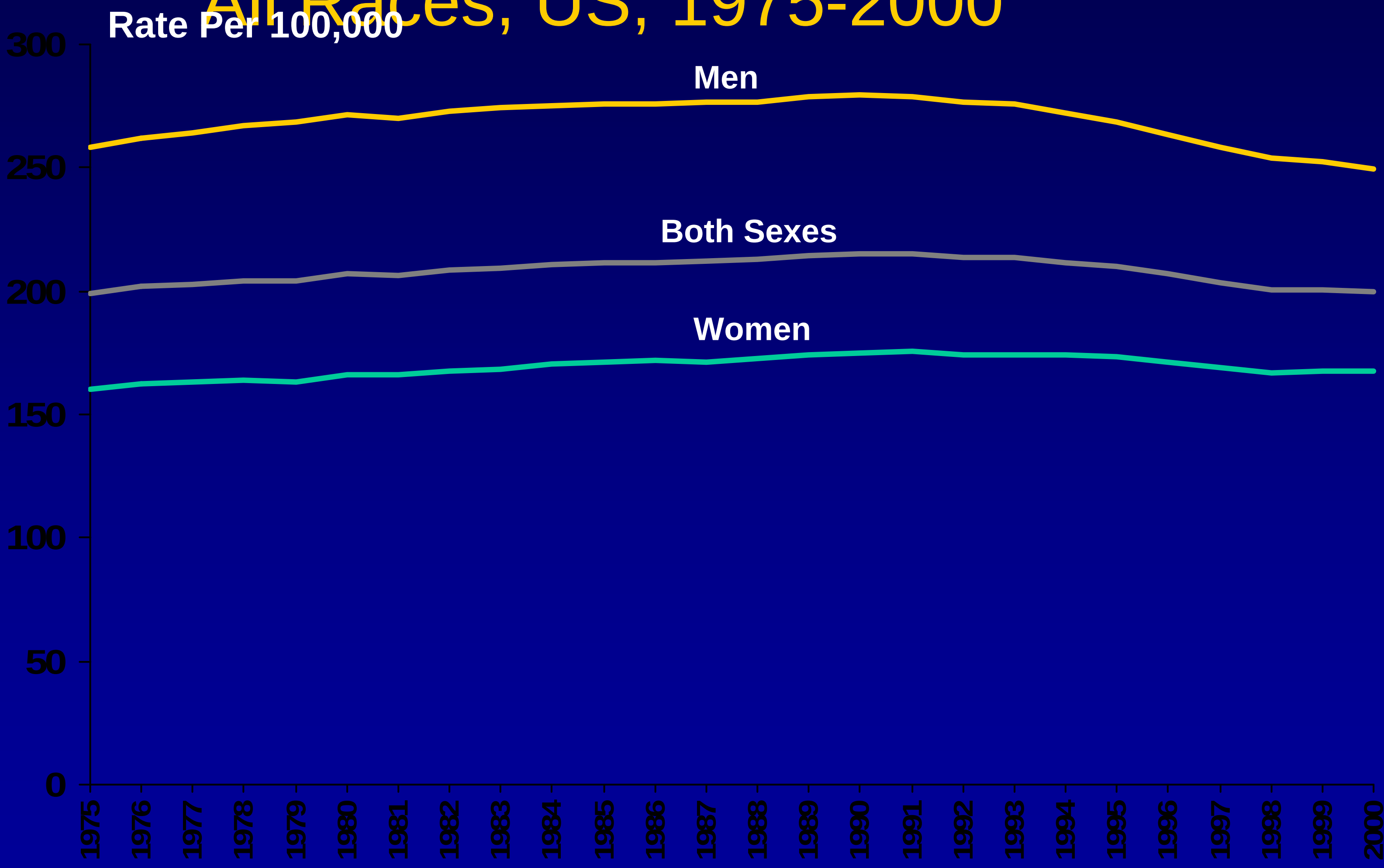

*Age-adjusted to the 2000 US standard population.
Source: Surveillance, Epidemiology, and End Results Program, 1975-2000, Division of Cancer Control and Population Sciences, National Cancer Institute, 2003.

Cytotoxic Drugs

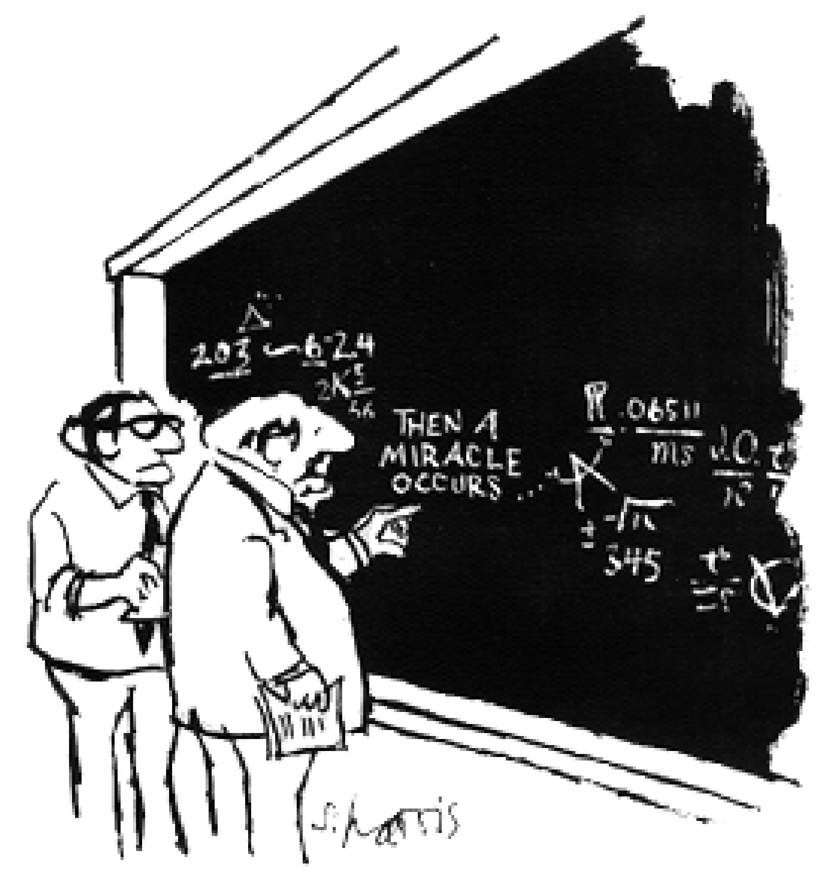

"I think you should be more explicit here in step two."

"They do more good than harm so use them"

most of the time

New Targeted Therapies

- EGFr Targeted Treatments
  - Iressa
  - Erbitux
- Antiangiogenic Therapies
  - Avastin
- Gene Therapies
  - P53 Adenovirus (Invitrogen)

Oligonucleotide therapeutics

Antigene Strategy

•Targets genomic DNA, *e.g. c-myc, c-ras, HIV genes*
•Sequence-specific tripe helix formation

Decoy Strategy

•Targets DNA binding protein, *e.g. E2F, NFκB*
•Sequestration of protein by oligonucleotide containing consensus sequence

Aptamer Approach

•Targets small molecules, proteins, *e.g. thrombin*
•Shape-specific recognition by combinatorially selected oligonucleotides

Immunomodulatory Oligonucleotides

•Vaccine adjuvants, allergy therapy
•Oligonucleotides containing 5’-CG motifs

Therapeutic Strategies

ANTIGENE

Target: DNA.
Effector: Single stranded oligo.
Mechanism: Triple helix formation.
Small Molecules

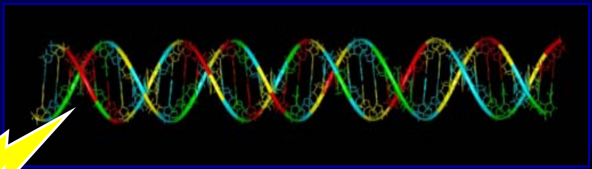

Genomic DNA

TRANSCRIPTION

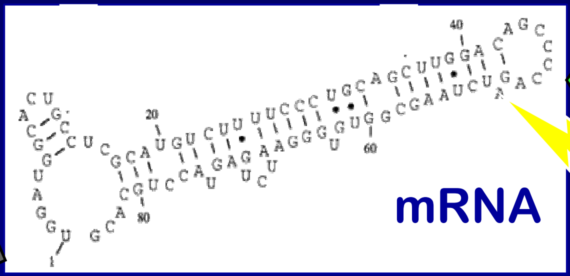

ANTISENSE

Target: mRNA.
Effector: Single stranded oligo.
Mechanism: Duplex formation, RNase H activation.
Small Molecules

TRANSLATION

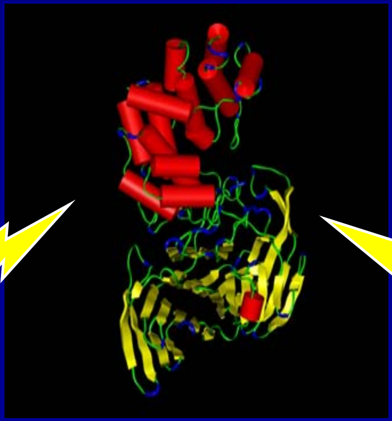

DECOY

Target: DNA-binding protein.
Effector: Double stranded oligo.
Mechanism: Decoy.
Small Molecules

APTAMER

Target: Protein.
Effector: Single stranded oligo.
Mechanism: Shape recognition.
Small Molecules

Antiproliferative properties of G-rich oligonucleotides

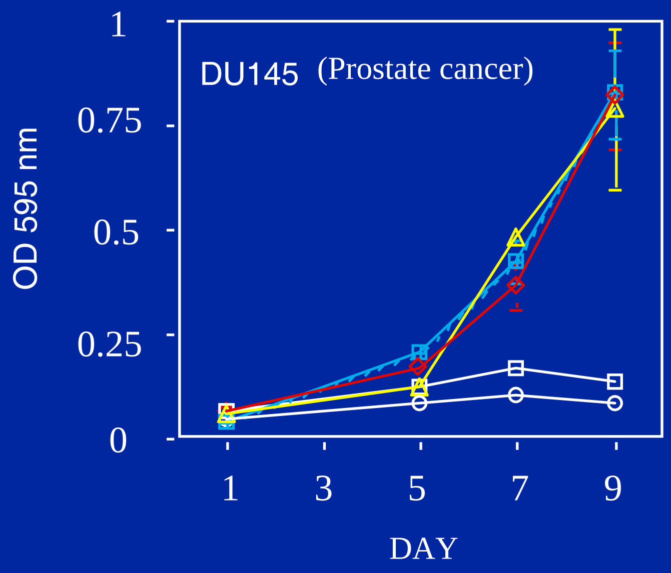

Symbol	Name	Sequence
□	GRO15A	5'-GTTGTTTGGGGTGGT-3'
◇	GRO15B	5'-TTGGGGGGGGTGGGT-3'
○	GRO29A	5'-TTTGGTGGTGGTGGTTGTGGTGGTGGTGG-3'
△	GRO26A	5'-GGTTGGGGTGGGTGGGGTGGGTGGGG-3'
⊞	WATER	

MTT Assay:

Showing growth of cells treated with four doses (days 1-4) of GRO.

Photographs of growth inhibited (GRO29A) and untreated MDA-MB-231 cells on day 7 after initial treatment (10x mag)

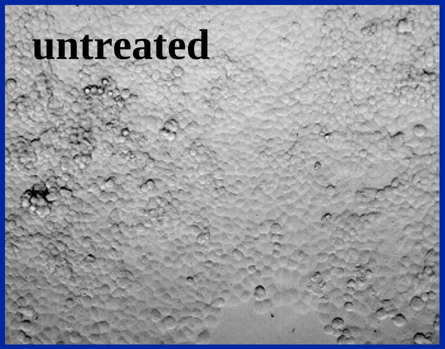

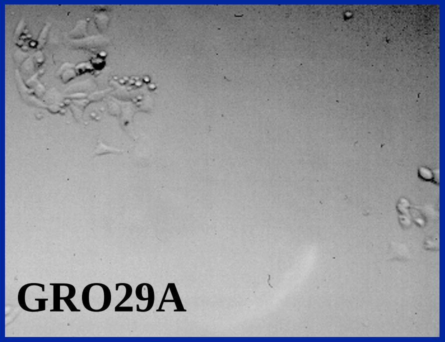

Inhibition is dose dependent

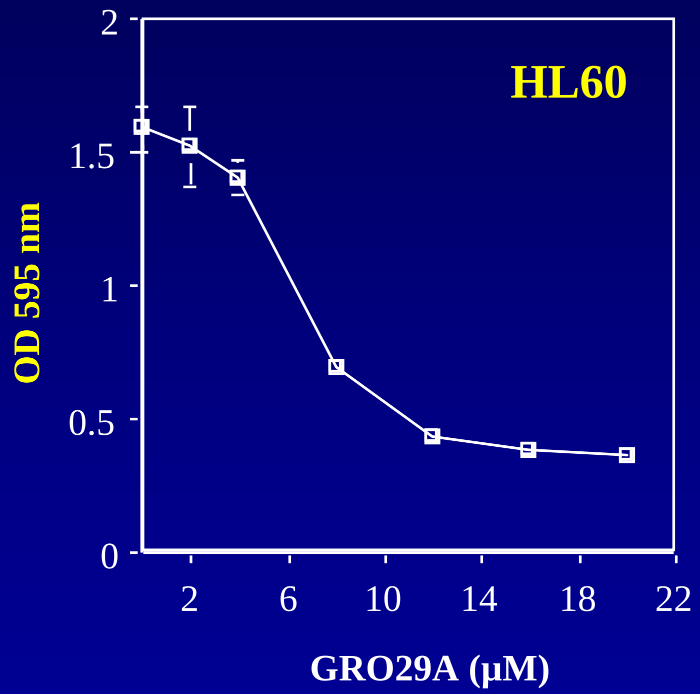

HL60 cells treated with a single dose of GRO29A on day 1 and assessed by MTT assay on day 7

Therapeutic potential of nucleolin-binding oligonucleotides

- **Synergy of GROs with conventional treatments?**

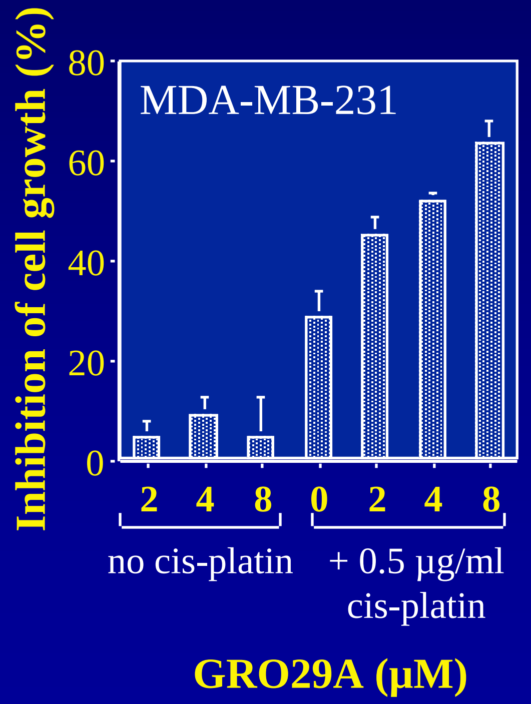

Many Types of Cancer Cells are Responsive to GRO Treatment

Cell Line [a]	Origin & Description	GRO response?
MDA-MB-231	Breast adenocarcinoma, tumorigenic[b], hormone ind.	Yes
MCF7	Breast adenocarcinoma, non-tumorigenic, hormone dep.	Weak[d]
DU145	Prostate adenocarcinoma, tumorigenic, hormone ind.	Yes
PC3	Prostate adenocarcinoma, tumorigenic, hormone ind.	Yes
CaLu1	Epidermoid carcinoma, lung, tumorigenic	Yes
HeLa	Adenocarnima of cervix, non-tumorigenic	Yes
HS27	Foreskin fibroblasts, derived from normal tissue	Weak[d]
'MTX'	Methotrexate-resistant MCF7 derivative[c]	No [e]
B16-F0	Mouse melanoma, tumorigenic	No
U937	Histiocytic lymphoma	Yes
K562	Chronic myelogenous leukemia	Yes
MEG01	Chronic myelogenous leukemia	Yes
RS4[11]	Acute lymphoblastic leukemia	Yes
10P2	Mast cell (represents normal hematopoietic precursor)	Weak[f]

Footnotes: (a) all human cells except 10P2 and B16-F0; (b) in nude mice; (c) K. H. Cowan et al., J. Biol. Chem. 257:15079 (1982); (d) compared to solid tumor cell lines; (e) may be due to unusual resistance of this cell line to cell cycle arrest; (f) compared to leukemia cell lines.

Antiproliferative oligonucleotides compete for binding to a protein that binds a G-quartet forming telomere sequence

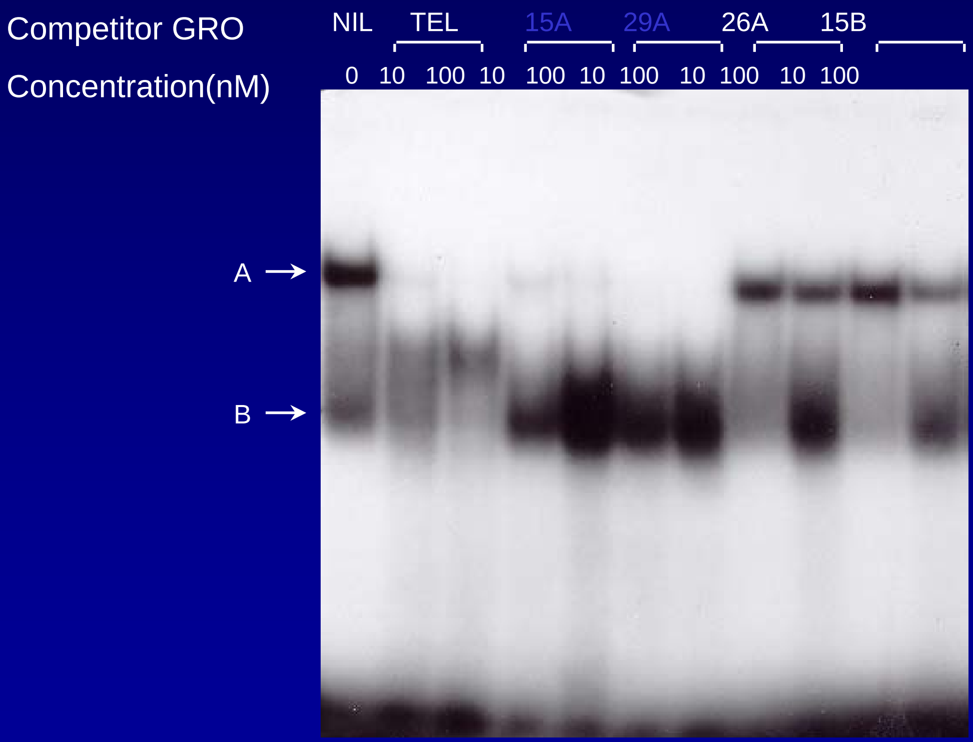

Electrophoretic Mobility Shift Assay:

^{32}P-TEL (5’-(TTAGGG)$_4$)
+ HeLa nuclear extracts
+ cold competitor GRO

Activity of G-rich oligonucleotides correlates with protein binding

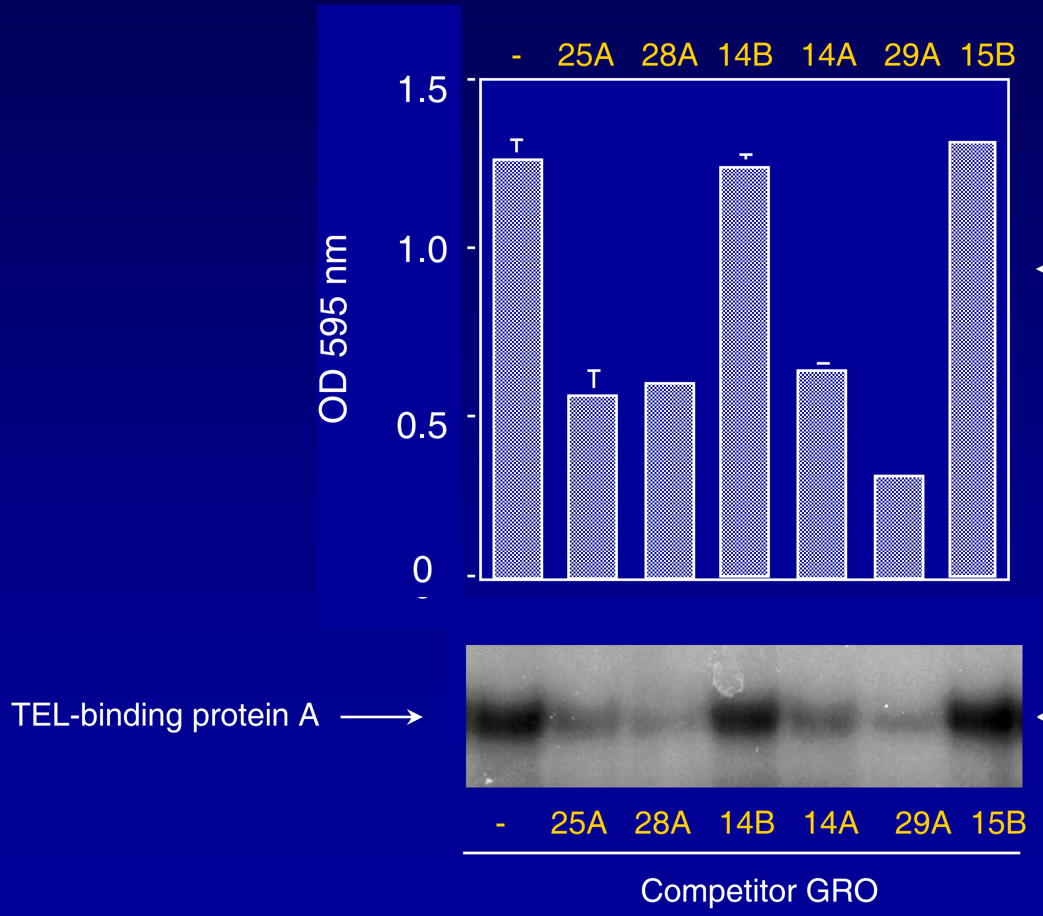

MTT Assay:

MDA-MB-231 treated with single dose (10µM) GRO. Day 7 after treatment.

Competition Mobility Shift Assay:

MDA-MB-231 nuclear ext. + ^{32}P-TEL + cold GRO

Nucleolin - structure

- Abundant, multifunctional phosphoprotein -707 aa
- Major non-ribosomal protein located in nucleoli

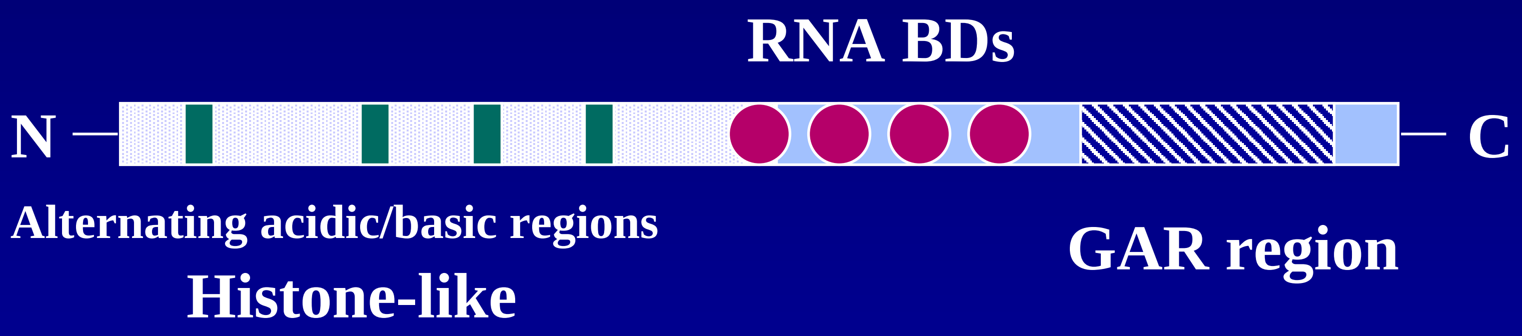

G-quartet Formation

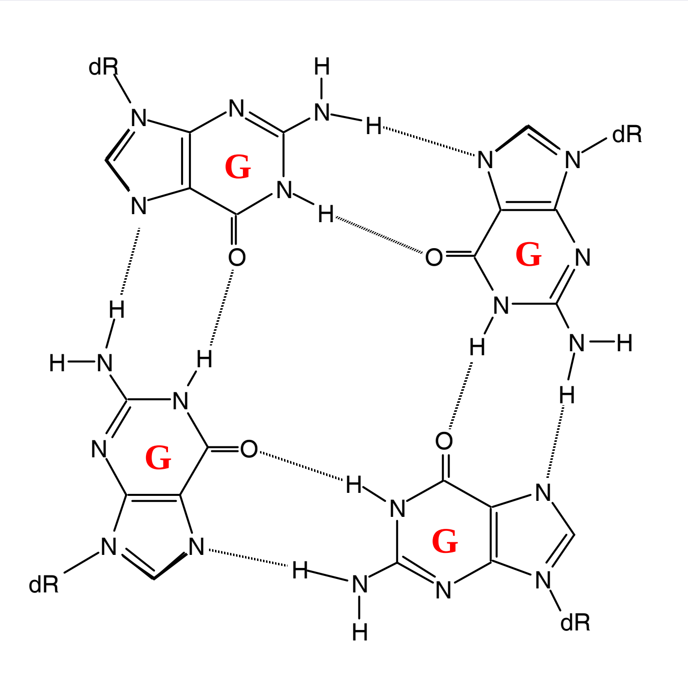

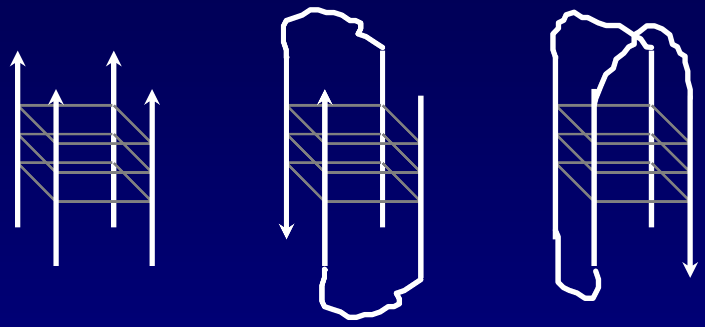

Tetramer Dimer Monomer

G-quartets in vivo?
•Telomeres
•Immunoglobulin switch regions
•HIV1 RNA
•Fragile X repeat
•Ribosomal DNA

"The Aptamera Story"

GROs: A new cancer treatment

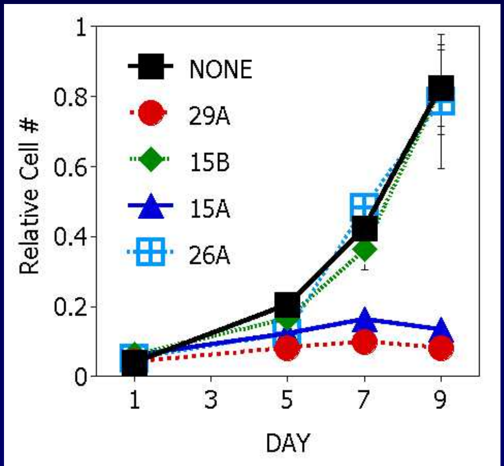

DU145 Cells

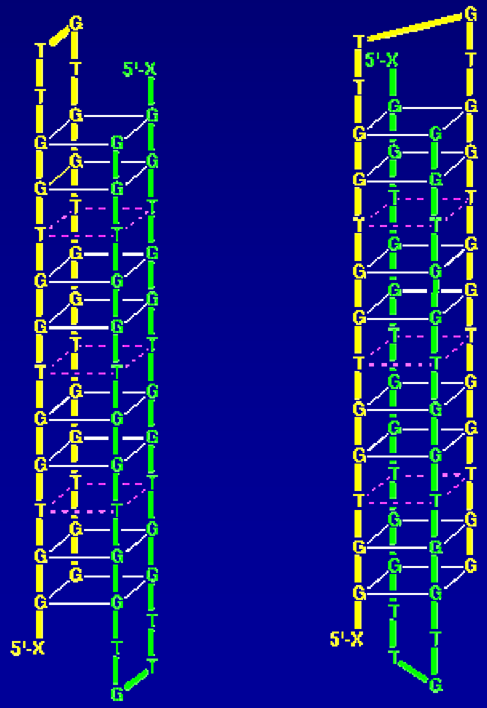

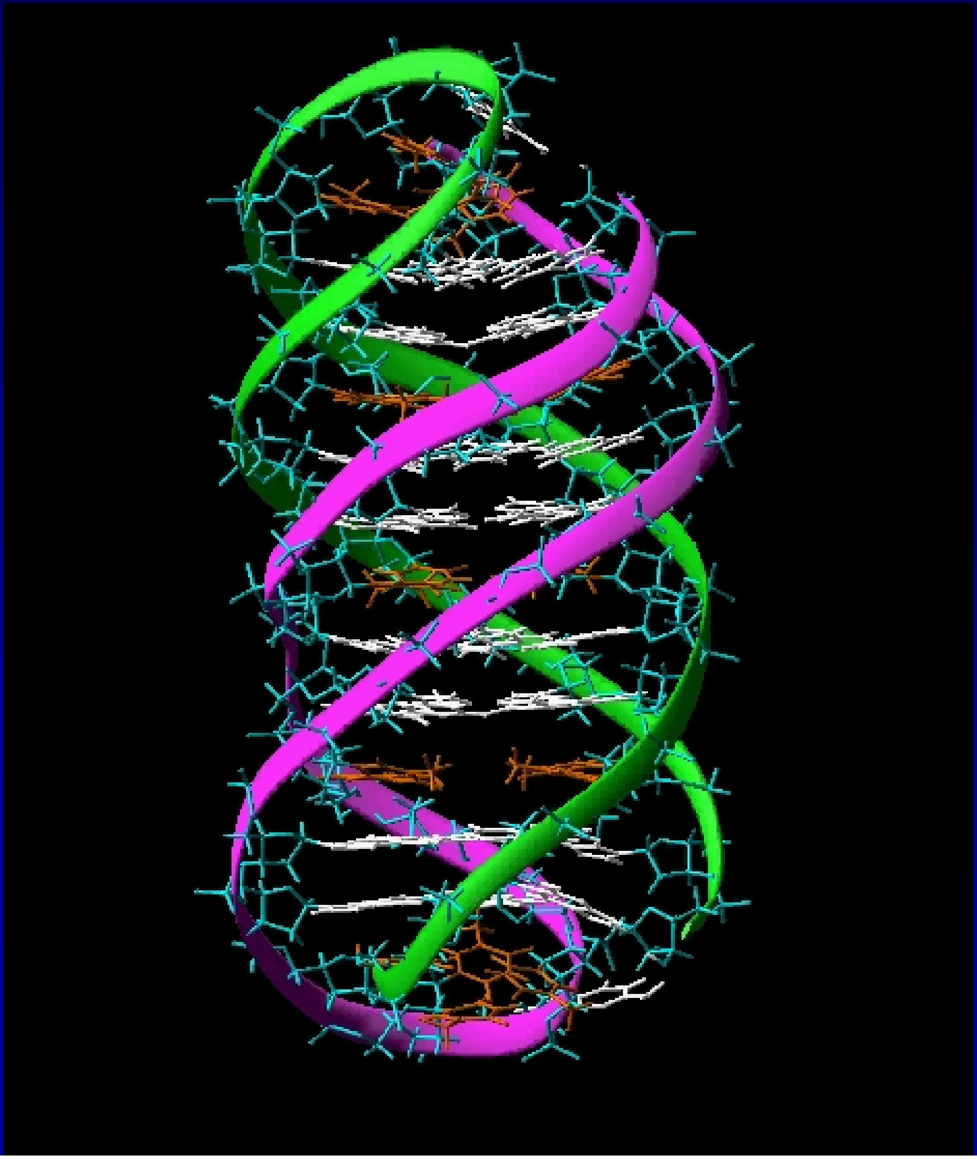

New Cancer Specific Target

PJ Bates, JO Trent, DM Miller

Nucleolin is on the surface of tumor cells but not “normal” cells

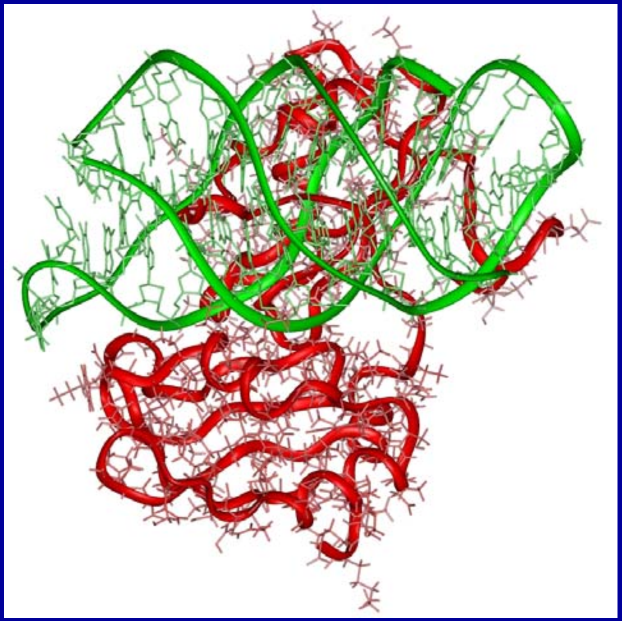

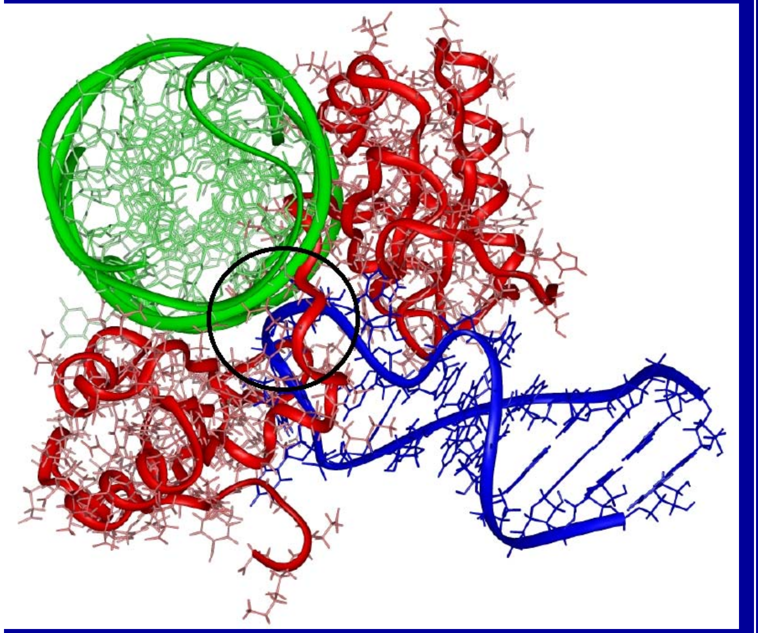

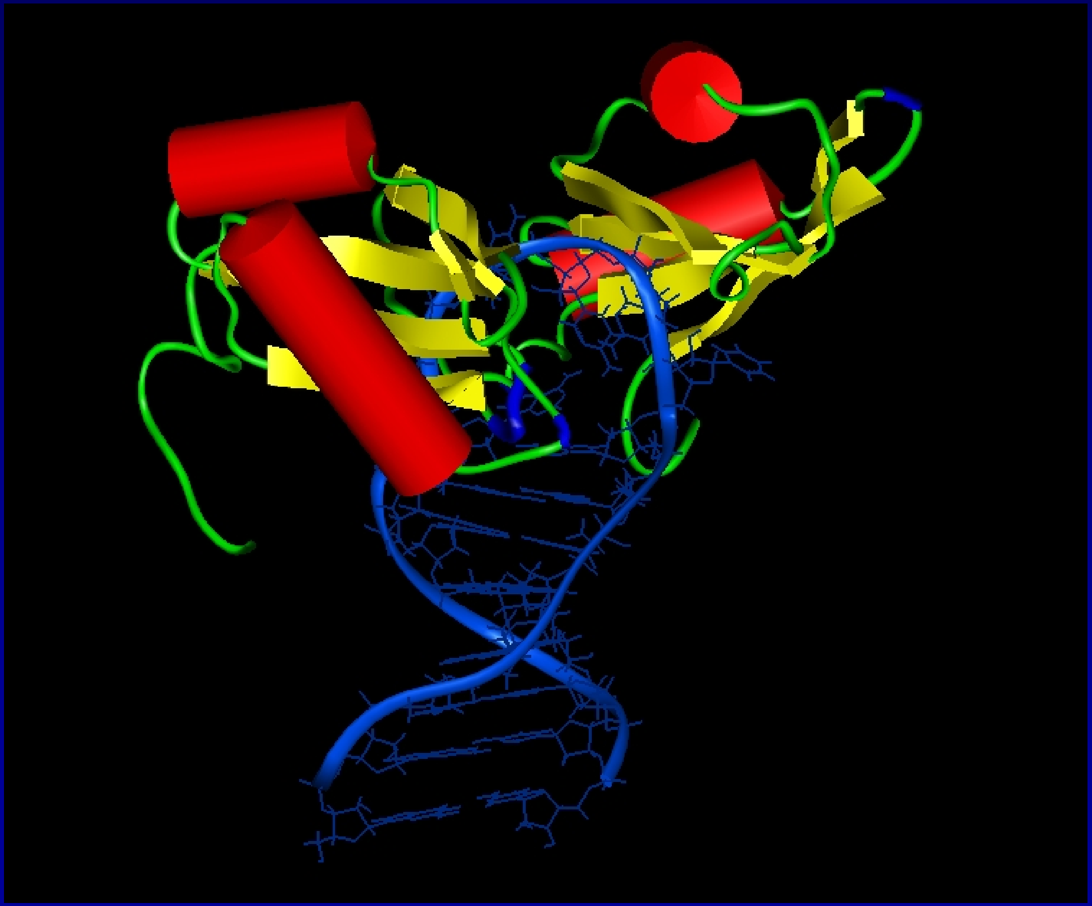

Nucleolin is Present on the SURFACE of Cancer Cells

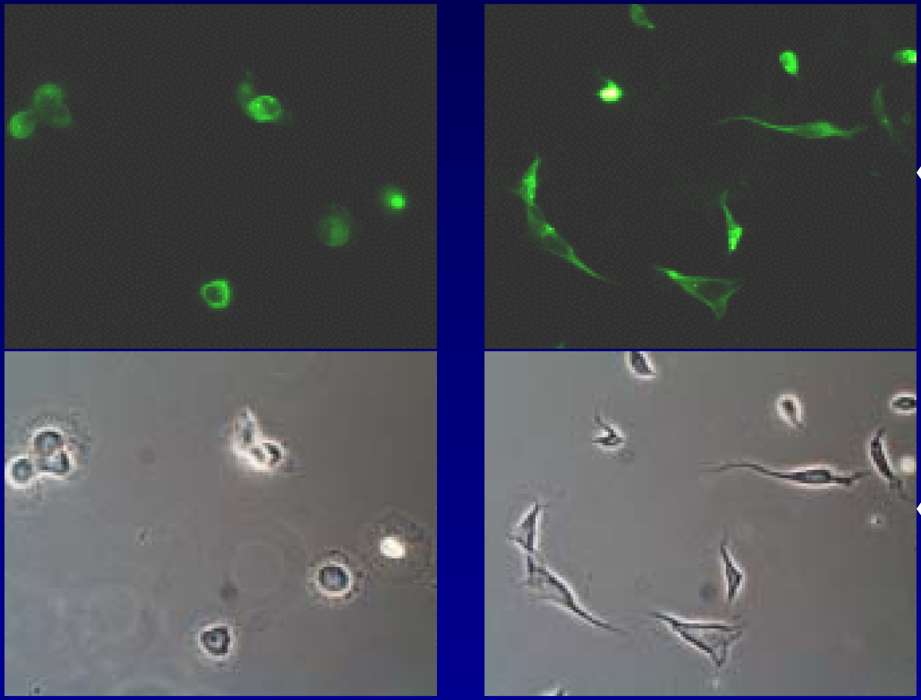

Immunofluorescent staining of non-permeablized cells with nucleolin antibody.

Phase contrast micrograph of same cells.

DU145 cells (prostate cancer)

MDA-MB-231 cells (breast cancer)

Speculative model of G-rich oligonucleotide action

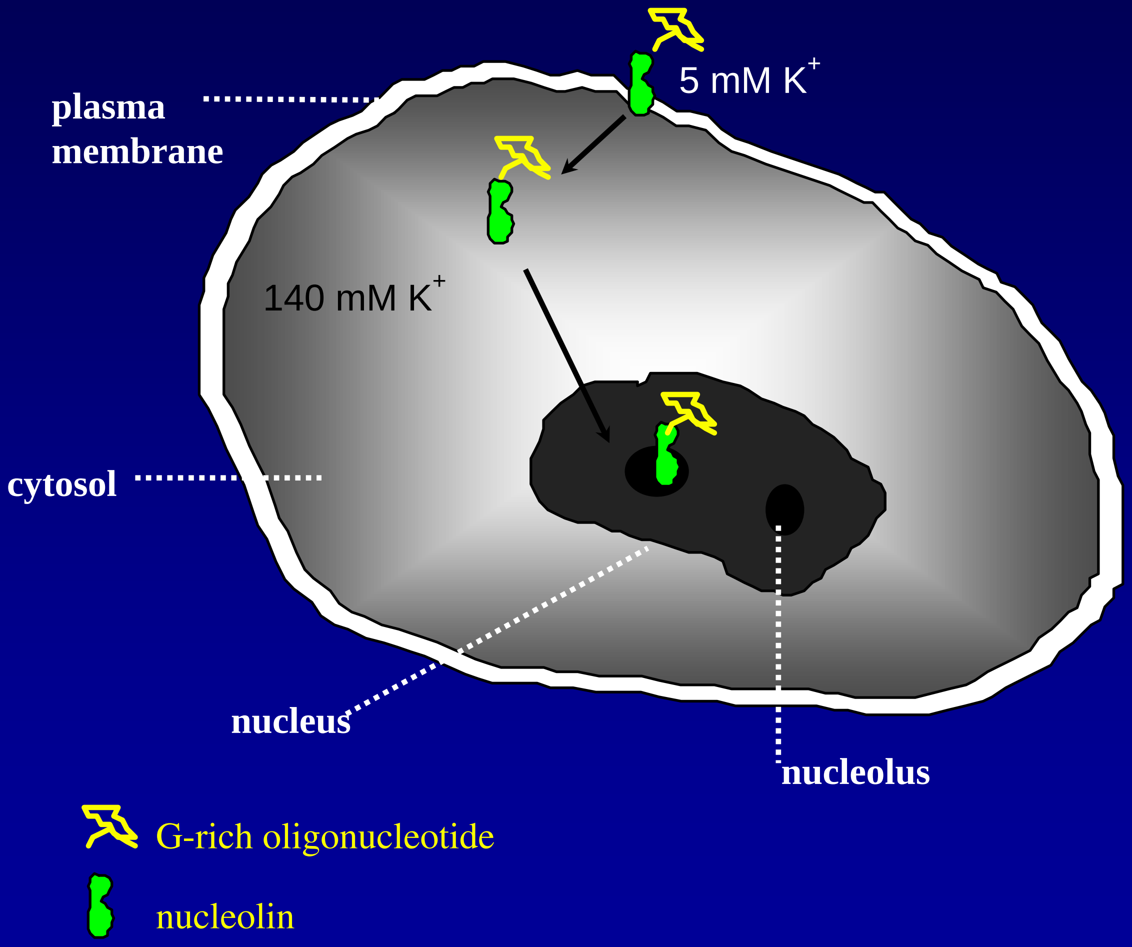

Functions of Nucleolin

- **Cell entry?**
- **Nuclear-cytosolic shuttle**
- **Ribosome biogenesis**
- **Chromosome comdensation**
- **DNA helicase?**

Regulation of Nucleolin

- **Phosphorylation**
- **Self-proteolysis**

Nucleolin - a G-quartet binding protein?

- **GROs**
- **Telomeres**
- **Ig switch regions (Maizels et al.)**
- **rDNA**

Phase I Clinical Trial

- 17 patients with end stage cancer were given a single four day infusion
- If there was evidence of response the patients were retreated
- There was no toxicity
- More than half the patients had stable disease for up to six months

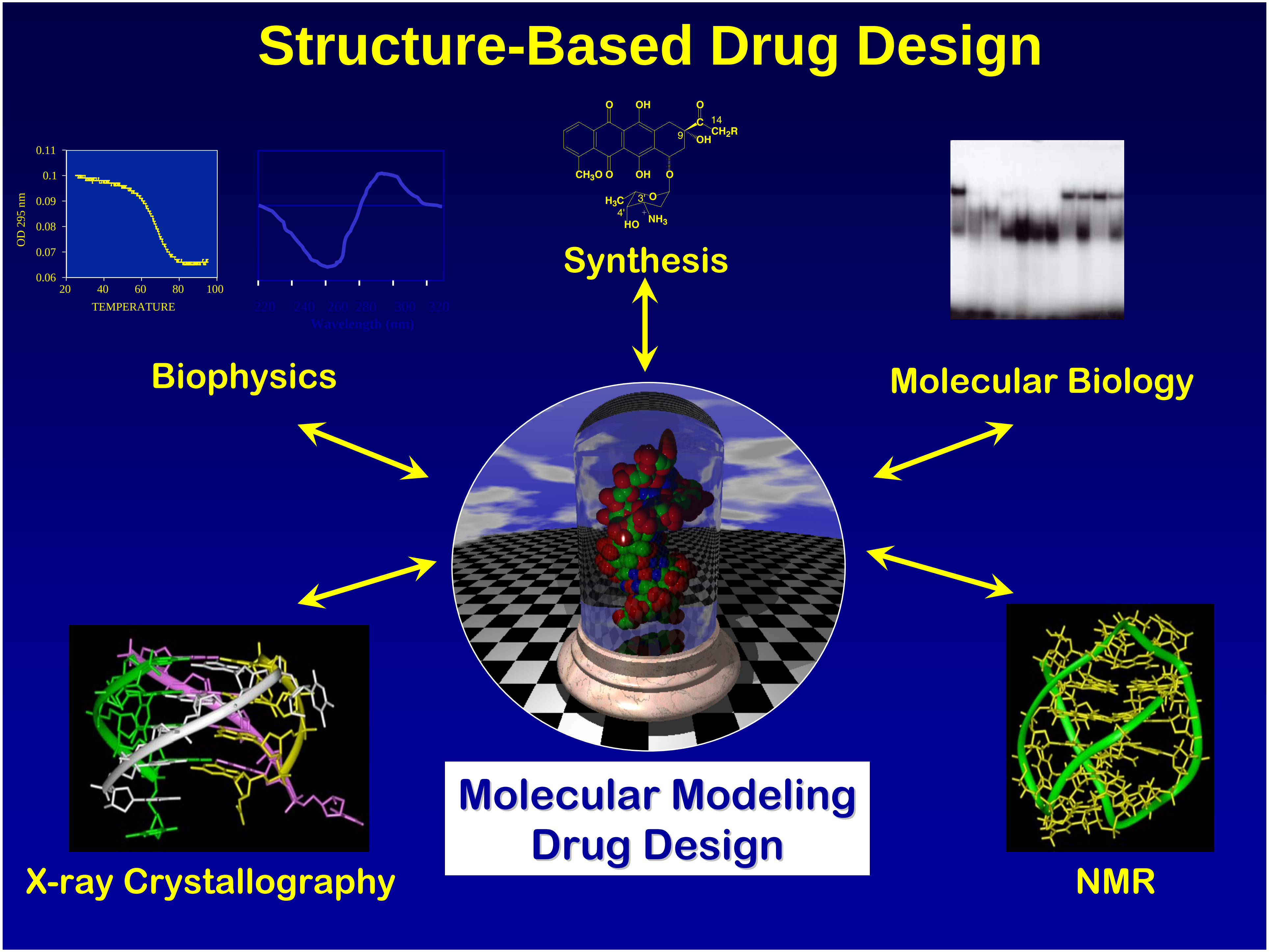
Structure-Based Drug Design
OD 295 nm
TEMPERATURE
Synthesis
Biophysics
Molecular Biology
Molecular Modeling
Drug Design
X-ray Crystallography
NMR

Investigator: JASON CHESNEY

Target: Tumor cell metabolism/ Inducible phosphofructokinase-2 which acts as a supercharger of anaerobic glycolysis

Progress: In collaboration with John Trent and Andrew Lane, has designed inhibitors, some of which have significant activity at low micromolar concentrations against neoplastic cell lines.

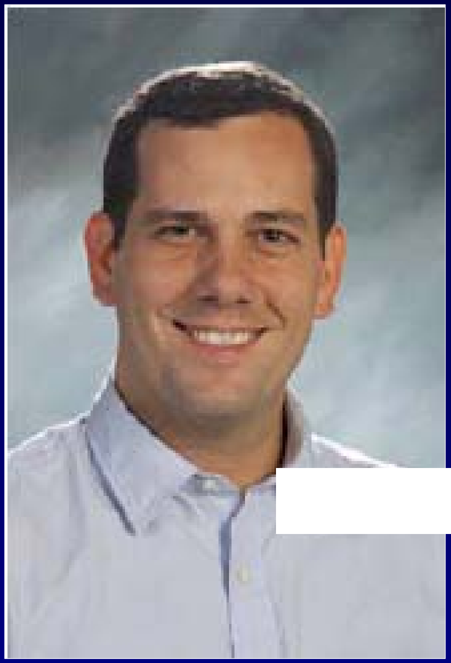

Investigator: NICK DELAMERE

Target: Disruption of tumor cell cation balance.

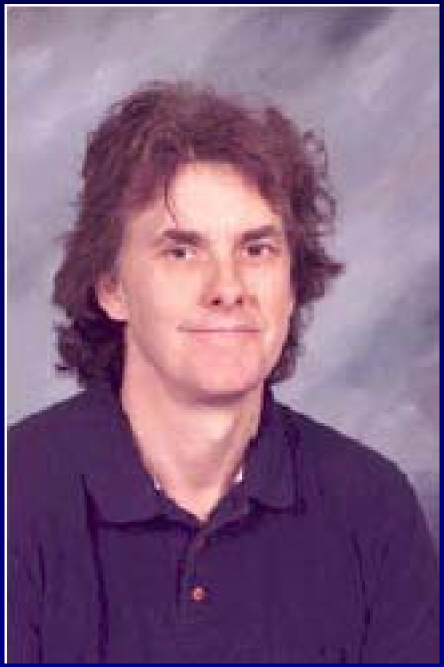

Progress: Inhibitors of Na/K ATPase (ouabain and derivatives) slow growth of transformed cells in sub-micromolar concentrations (i.e., lower than required for typical Na/K ATPase inhibition).

Investigator: GLENN McGREGOR

Target: Cancer prevention through targeted inhibition of error-prone DNA repair

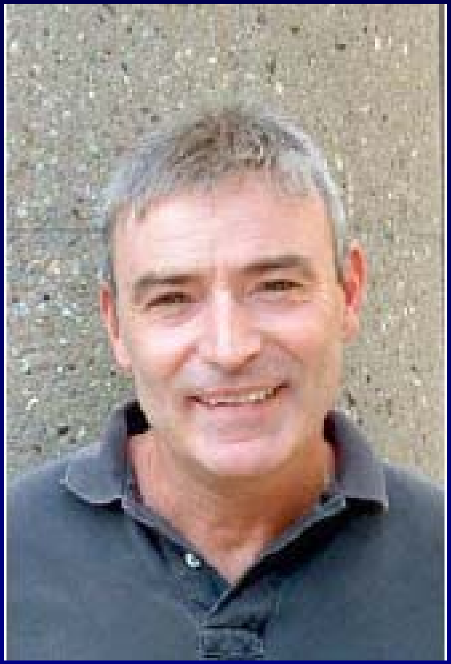

Progress: Has developed anti-sense strategies for in vitro (and possibly in vivo) inhibition of error-prone DNA repair enzyme which, in vitro, suppresses UV-mediated mutagenesis. Possible application in sunscreens.

Investigator: Bob Mitchell

Target: Macrophage/monocyte migration inhibitor factor (MIF) a cytokine with enzymatic activity which is necessary for cell tranformation.

Progress: With John Trent, he has designed and tested small-molecule inhibitors of MIF which selectively arrest the migration, invasion and survival of transformed cells.

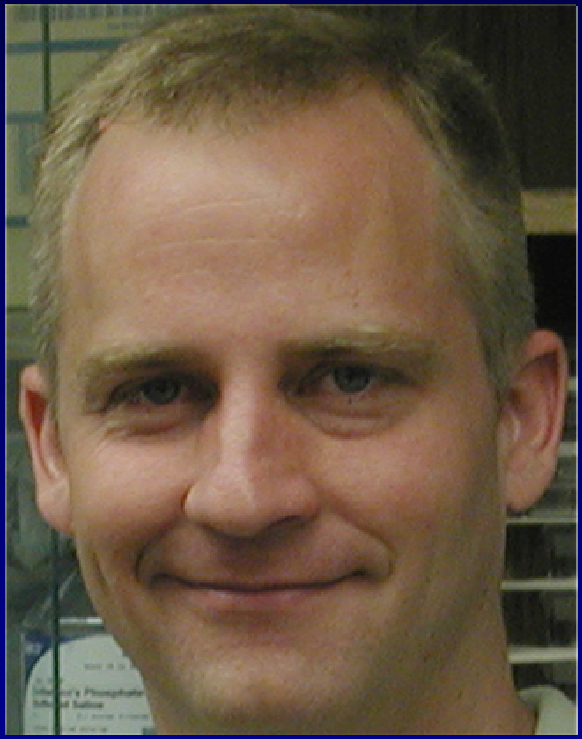

DNA contains all of the secrets of nature. If we could only sequence the human genome we could understand every human disease. Unfortunately, that will not happen in any of our lifetimes.

- James Watson, Durham, NC, 1967

Owensboro Cancer Research Center

- Large Scale Biology
  - Company based in Vacaville, CA
  - Production facility in Owensboro, KY
    - Produces GMP grade peptide pharmaceuticals from tobacco
    - Use TMV vectors to infect adult plants
    - Have produced antibodies for clinical trial with Stanford
  - Ben Jenson is developing an HPV vaccine to be produced in tobacco with LSBC

Owensboro Cancer Research Center

- Collaboration between JGBCC and Owensboro community
- To be funded by federal grants to Owensboro/UofL
- $30 M/ten year project to develop research facility to develop cancer peptide therapeutics from tobacco

Owensboro Cancer Research Center

- Ten UofL faculty based in Owensboro
- Will include plant molecular biologists, virologists, translational scientists
- One goal will be to translate small molecule therapeutics to peptide therapeutics
- Construction has begun

Drug Design at JBCC

- Molecular Modeling Core
- NMR Facility
- Xray Crystallography
- Biophysics core lab
- Protein Expression and purification
- Micropet Imaging Facility

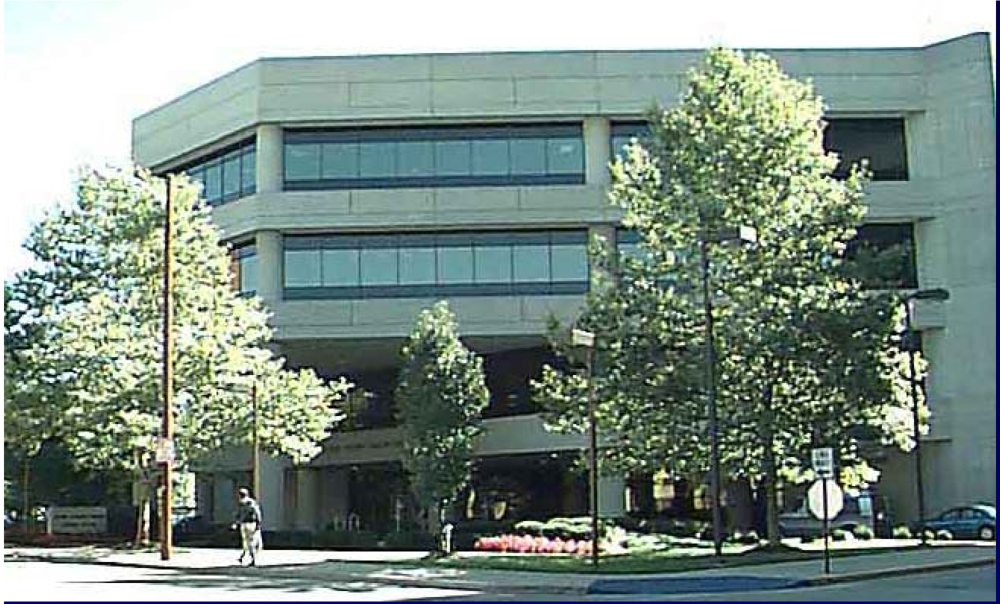

James Graham Brown Cancer Center

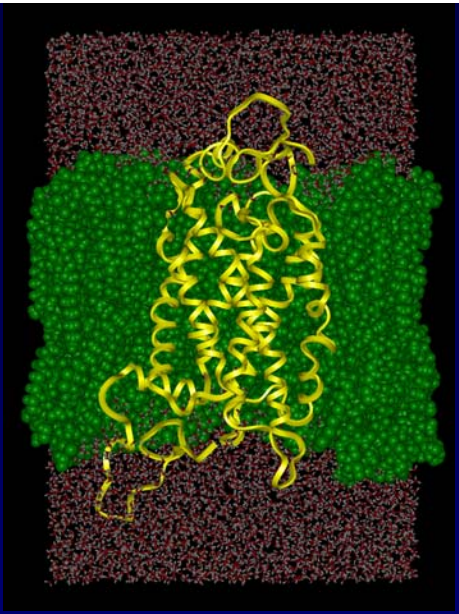

BCC Computational Laboratory

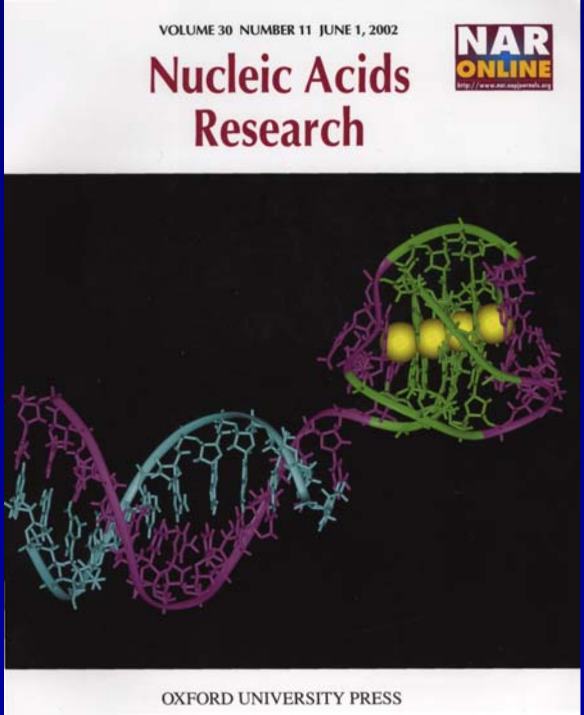

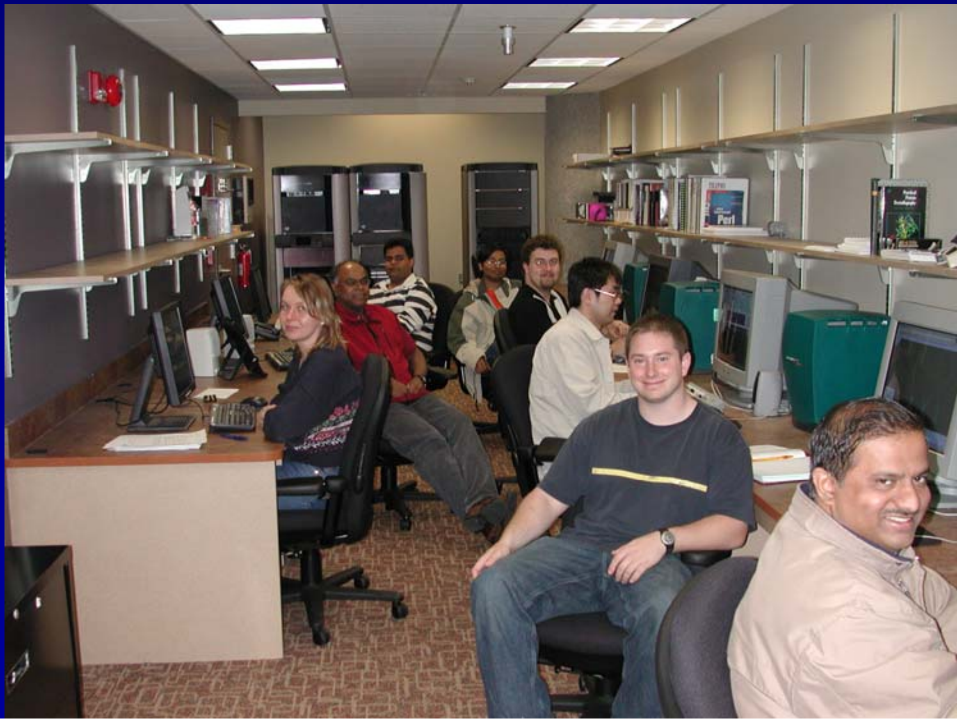

New Targets

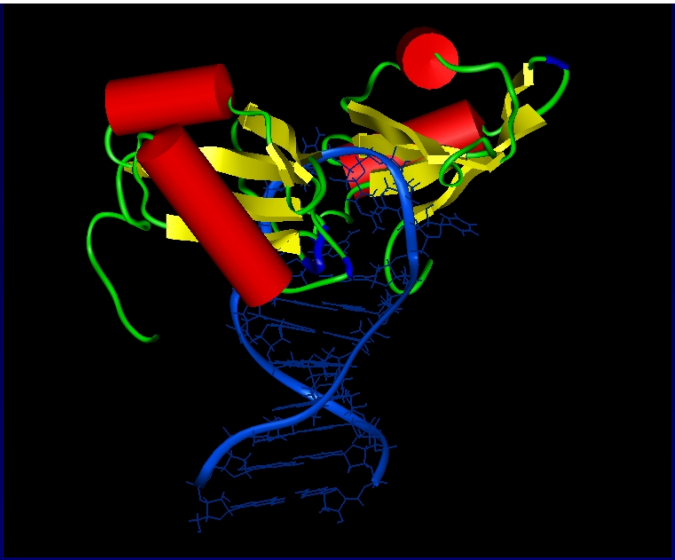

Nucleolin

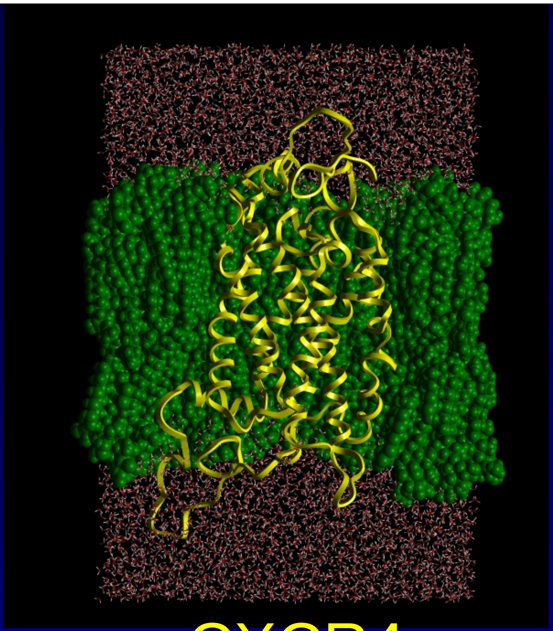

CXCR4

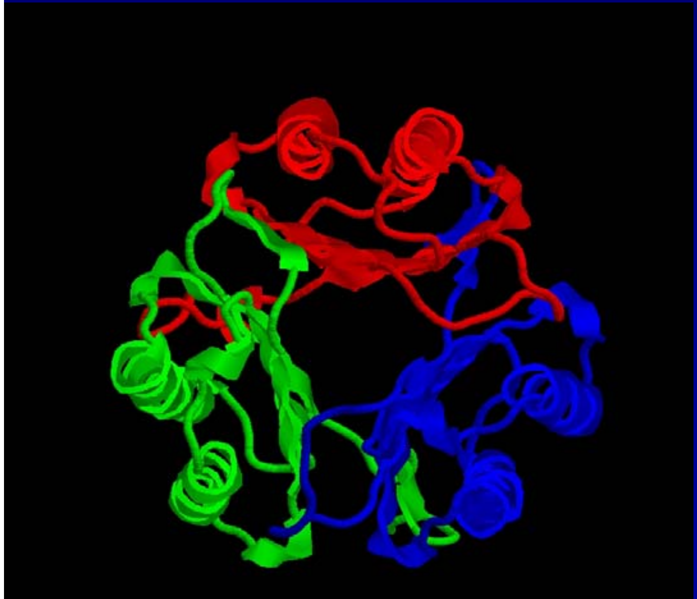

MIF

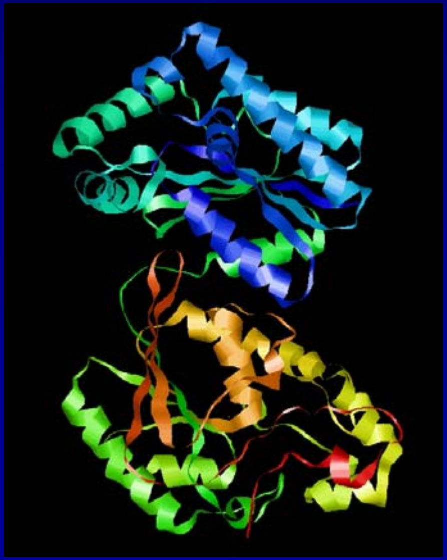

iPFK-2

NMR Center Facilties

- 600 and 800 MHz 4-channel NMR spectrometers
- Proteins and nucleic acids
- Target-drug interactions
- Automation for metabolomics and screening

14.1 T magnet with SMS robot

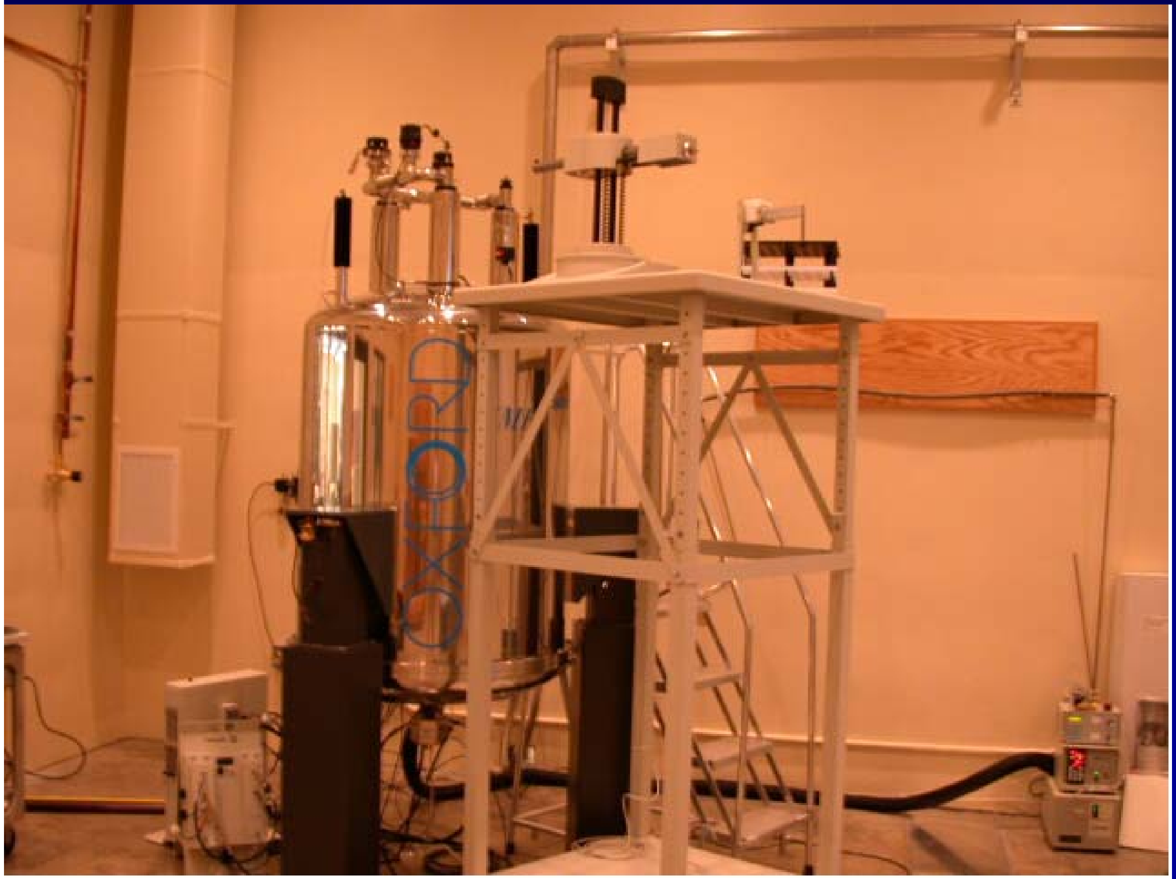

14.1 T spectrometer

14.1 T Oxford shielded magnet-51 mm bore (4.2 K)
Varian Inova 4-channel console
+ Robot

Drug Discovery
Virtual Screening of Small Molecules

Computationally screen databases of small molecules (6,000,000)

To physically test these at 1000 molecules per day = 16.4 years

To *in silico* screen these = 20 days

Nucleolin
In silico screen of 300,000 compounds = 48 hours
50 out of 200 "hits" were selected
25 biologically tested

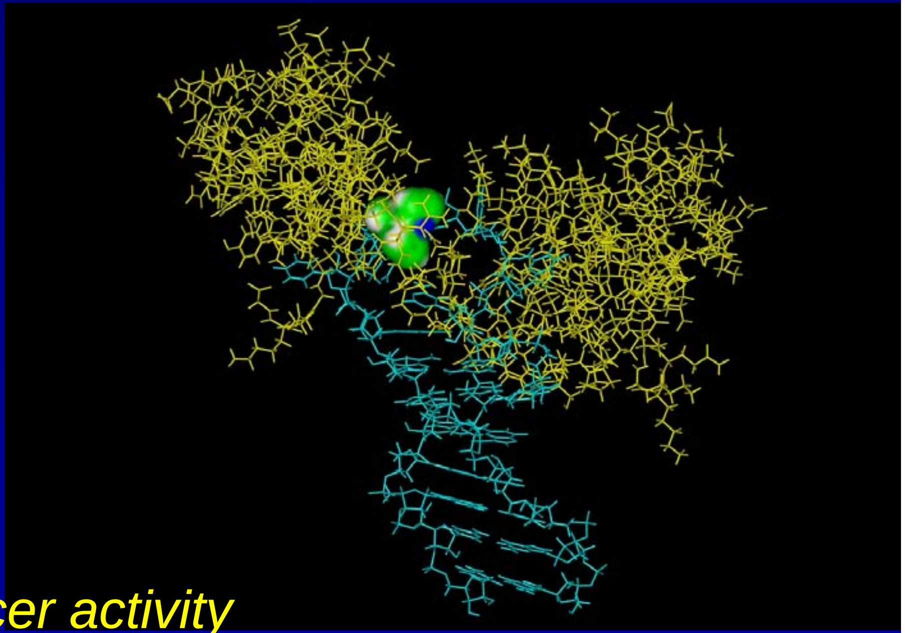

Compounds "C", "N"

Cancer selective
Competes with GROs
Active in lead compound range
Never been tested in cancer before
Can buy in 100kg drums
National Cancer Institute confirms cancer activity

Center for Macromolecular Interactions

A Biophysical Core Facility for the BCC

(Supported by a Kentucky New Economy Grant)

Dr. J. Brad Chaires, Director

Dr. Nichola Garbett, Assistant Director

State-of-the-art instrumentation for:

Calorimetry

- Differential Scanning Calorimeter (DSC)
- Isothermal Titration Calorimeter (ITC)
- Automated High-throughput DSC (1 of only 4 in academia)

Spectroscopy

- UV/Vis Spectrophotometer
- Fluorescence Spectrometer
- Circular Dichroism (simultaneous flourescence; auto titrator)
- Surface Plasmon Resonance (SPR – Biacore)
- Rapid Scan Stopped-Flow Spectrometer
