# Supplementary material for: Meeting materials from the 3rd Annual Meeting of the International Society for the Prevention of Tobacco Induced Diseases
Source: Tob Induc Dis. 2004 Dec 15;2(4):168. doi: 10.1186/1617-9625-2-4-168 (PMC2671527; doi:10.1186/1617-9625-2-4-168)
Supplement: Additional file 1 [file 1617-9625-2-4-168-S1.zip › Abstract 9-Novel therapies and treatments for lung cancer.pdf]

Abstract 9

Saturday 16.30

Novel therapies and treatments for lung cancer

Donald M. Miller, James Graham Brown Cancer Center
Louisville, KY, USA

The Brown Cancer Center is actively engaged in the efficient translation of state of the art discoveries and developments for the treatment of a range of cancers. This presentation will outline some novel approaches in the treatment of lung cancer and will demonstrate how the speedy translation of basic science findings in cancer can be developed into novel therapies and treatments for the harrowing smoking related condition, lung cancer.
