# Supplementary material for: Meeting materials from the 3rd Annual Meeting of the International Society for the Prevention of Tobacco Induced Diseases
Source: Tob Induc Dis. 2004 Dec 15;2(4):168. doi: 10.1186/1617-9625-2-4-168 (PMC2671527; doi:10.1186/1617-9625-2-4-168)
Supplement: Additional file 1 [file 1617-9625-2-4-168-S1.zip › Abstract 10-Chronic obstructive pulmonary disease and diabetes.pdf]

Abstract 10

Saturday 17.00

Chronic obstructive pulmonary disease and diabetes

Christine S. Ritchie, MD, MSPH
University of Alabama at Birmingham, USA

Chronic obstructive pulmonary disease is expected to be the third commonest cause of death by 2020. Furthermore, COPD has been recognized as being the twelfth greatest cause of chronic morbidity, with a predicted increase to become the fourth most important disability-producing illness by 2020. Smoking is the primary risk factor for the development of COPD. How tobacco leads to COPD is not completely clear but a number of mechanisms are being elucidated, including the impact of tobacco on oxidative biology, immune function and alveolar destruction, which will be discussed.
