# Supplementary material for: Meeting materials from the 3rd Annual Meeting of the International Society for the Prevention of Tobacco Induced Diseases
Source: Tob Induc Dis. 2004 Dec 15;2(4):168. doi: 10.1186/1617-9625-2-4-168 (PMC2671527; doi:10.1186/1617-9625-2-4-168)
Supplement: Additional file 1 [file 1617-9625-2-4-168-S1.zip › Abstract 11-Periodontitis and its association to other diseases and smoking.pdf]

Abstract 11

Sunday 08.30

Periodontitis and its association to other diseases and smoking

Robert J. Genco, DDS, PhD
Oral Biology and Microbiology, State University of New York at Buffalo, USA.

Risk factors for periodontal disease include cigarette smoking, diabetes mellitus, osteoporosis, low levels of dietary calcium, and genetic factors. Smoking is by far the most important risk factor, increasing the risk of periodontal disease 4 to 5-fold, even at moderate levels of smoking.

Periodontal disease is also known to affect systemic diseases. For example, periodontal disease increases the severity of and decreases glycemic control in diabetes mellitus. Treatment of periodontal disease results in reduction of glycated hemoglobin levels. Periodontal disease is also a strong, independent risk factor for myocardial infarction (MI) in young individuals (less than 55 years of age). Furthermore, cigarette smoking and periodontal disease are independent risk factors in incident MI in this population. Periodontal disease also increases the risk for low birth weight infants, and in compromised patients, periodontal disease increases the risk for respiratory infection.

Since heart disease is the leading cause of death in the United States, and periodontal disease is very prevalent, affecting 10 to 30% of the population dependent upon age, it is reasonable that control of periodontal disease is likely to reduce the risk for myocardial infarction. Furthermore, control of both oral disease and smoking may reduce the risk for cardiovascular disease in a significant manner. Intervention trials are needed to determine the extent to which the risk for MI and other atherosclerotic diseases will be reduced by combined reduction of oral disease and smoking.
