# Supplementary material for: Meeting materials from the 3rd Annual Meeting of the International Society for the Prevention of Tobacco Induced Diseases
Source: Tob Induc Dis. 2004 Dec 15;2(4):168. doi: 10.1186/1617-9625-2-4-168 (PMC2671527; doi:10.1186/1617-9625-2-4-168)
Supplement: Additional file 1 [file 1617-9625-2-4-168-S1.zip › Abstract 12-Full PDF presentation.pdf]

Smoking and Pancreatic Cancer: Animal Models of Pancreatic Dysfunction

Parimal Chowdhury, Ph.D.

University of Arkansas for Medical Sciences

Previous Surgeon General Reports

- 1972, significant association between cigarette smoking and pancreatic cancer
- 1979, dose response relationship between cigarette smoking and pancreatic cancer
- 1982, cigarette smoking as contributing factor in the development of pancreatic cancer
- 1989, cigarette smoking contributed to pancreatic cancer deaths in 29% men and 34% women
- 1990, there is a weak but consistently observed association between smoking and pancreatic cancer

2004 Surgeon General's Report

- In 2003, 30,000 deaths attributed to pancreatic cancer
- Lagged relationship between the prevalence of cigarette smoking and mortality from pancreatic cancer
- Median time from diagnosis to death is about three months

Death Rates In Smoking Men And Women Adjusted For Family History Of First Degree Relative

**Men**	
**Current smokers**	**2.1 (1.9-2.4)**
**<10 cigarettes/day**	**1.8 (1.4-2.5)**
**10-19 cigarettes/day**	**1.7 (1.3-2.2)**
**20 cigarettes/day**	**2.1 (1.8-2.6)**
**>20 cigarettes/day**	**2.4 (2.0-2.8)**
**p value for trend = 0.03**	

**Women**	
**Current smokers**	**2.0 (1.8-2.3)**
**<10 cigarettes/day**	**1.2 (0.9-1.6)**
**10-19 cigarettes/day**	**1.9 (1.6-2.2)**
**20 cigarettes/day**	**2.3 (1.9-2.7)**
**>20 cigarettes/day**	**2.3 (1.9-2.8)**
**p value for trend = 0.001**	

Death rates were standardized to the CPS-II population; RRs were adjusted for age; race; years of education; family history of pancreatic cancer in first-degree relative; history of gallstones; history of diabetes; BMI; and consumption of alcohol, total red meat, citrus fruits and juices, and vegetables.

Risk Estimates For Urban/Rural Place Of Residence

Findings	Risk estimates (95% C1)		Comments
● Significant risk for women smoking 2.5 cigarettes/day		RR	Risk estimates were adjusted for urban/rural place of residence
	Male cigarette behavior		
	Never smoked	1.0 (referent)	
	Former smokers	0.9 (0.6-1.5)	
	1-4 cigarettes/day	0.9 (0.5-1.8)	
	5-9 cigarettes/day	1.0 (0.5-2.1)	
	10-14 cigarettes/day	1.3- (0.7-2.4)	
	25 cigarettes/day	1.6 (0.8-3.2)	
	Female cigarette behavior		
	Never smoked	1.0 (referent)	
	Former smokers	0.6 (0.2-1.5)	
	1-4 cigarettes/day	0.9 (0.4-1.8	
	25 cigarettes/day	1.8 (1.1-3.0)	

Risk Ratio Adjusted For Gender and Age

No significant associations

	**RR**
**Nonsmokers**	**1.00 (referent)**
**Former smokers**	**0.75 (0.42-1.43)**
**Current smokers**	**1.39 (0.96-1.99)**
**Light smokers**	**1.37 (0.94-2.00)**
**Regular smokers**	**1.25 (0.75-2.08)**
**Cigars or pipes**	**0.58 (0.28-1.19)**

RRs were adjusted for gender and age

Age Distributions and Mortality Risk Ratio

Mortality risk ratios

**Men**	
**1-19 cigarettes/day**	
**Aged 35-49 years**	**1.4**
**Aged 50-64 years**	**1.8**
**Aged 65-79 years**	**1.8**
**Aged >80 years**	**1.1**
**Women**	
**1-19 cigarettes/day**	
**Aged 35-49 years**	**2.4**
**Aged 50-64 years**	**1.5**
**Aged 65-79 years**	**1.4**
**Aged >80 years**	**1.3**

Comments

Age distributions were standardized using the 1980 distribution of the U.S. population p values and 95% Cis were not provided

Risk Ratio And History Of Diabetes Mellitus

Significant dose-response relationship for men and women with pack-years

RRs were adjusted for age, gender, BMIs, and history of diabetes mellitus

**Men**	**RR**
Never smoked	1.0 (referent)
Former smokers	1.3 (0.7-2.3)
Current smokers	3.0 (1.5-6.3
Pack-years	
Never smoked	1.0 (referent)
1-10 years	0.9 (0.3-2.6)
11-25 years	1.3 (0.7-2.7)
26-50 years	1.5 (0.7-3.1)
>50 years	2.8 (1.3-5.7)
p value for trend = 0.004	

**Women**	**RR**
Never smoked	1.0 (referent)
Former smokers	1.3 (0.7-2.3)
Current smokers	3.0 (1.5-6.3
Pack-years	
Never smoked	1.0 (referent)
1-10 years	0.9 (0.3-2.6)
11-25 years	1.3 (0.7-2.7)
26-50 years	1.5 (0.7-3.1)
>50 years	2.8 (1.3-5.7)
p value for trend = 0.004	

Biologic Basis for Pancreatic Cancer (Human Studies)

- Mostly occur in exocrine cells lining the pancreatic ductules
- Large body of evidence showing mutations in cellular proto-oncogenes and tumor suppressor genes
- Highest frequency of ras mutations found in adenocarcinoma of the pancreas
- K-ras mutations are an early and key event in the pathogenesis of pancreatic cancer and is higher in smokers than in non-smokers

Biologic Basis for Pancreatic Cancer (Animal Studies)

- Feeding tobacco specific N-nitrosamines, NNK, to rats induced invasive tumors. Nitrosamines, administered parenterally induced pancreatic cancer in rats
  (Rivenson, 1988)

Possible suggested mechanism:

1. Tobacco specific carcinogens may reach the pancreas through blood or refluxed bile that is in contact with pancreatic duct
2. High levels of nitrosamines and aromatic amines in cigarette smoke undergo metabolic activation probably in the liver and pancreas, bind to DNA causing mutations

Evidence Synthesis

- Case control and cohort designs show an increased risk of pancreatic cancer in smokers
- Dose response relationship of risk with the amount smoked
- Ras mutations in pancreatic cancer and induction of malignancy in animals in response to NNK
- Risk remains high after allowing the confounding factors such as alcohol consumption

Conclusion and Implications

- Evidence is sufficient to infer a causal relationship between smoking and pancreatic cancer
- Smoking prevention and cessation are the only potentially effective strategies for reducing the occurrence of pancreatic cancer

RISK FACTORS FOR PANCREATITIS AND PANCREATIC CANCER

- SMOKING
- ALCOHOL
- NICOTINE
- DIET
- OTHER

WHY STUDY NICOTINE?

- **PRESENT IN CIGARETTE SMOKE, SMOKELESS TOBACCO**
- **ADDICTIVE**
- **CNS STIMULANT**
  PROVIDES PLEASURABLE SENSATION
- **A COMMON DRUG OF ABUSE**
- **LONGTERM EFFECTS ON THE PANCREAS UNKNOWN**
- **COMMERCIALLY AVAILABLE IN ALMOST PURE FORM**
- **COULD BE SOLUBILZED AND ADMINISTERD VIA IV,INGESTION AND INHALATION ROUTES**
- **CHRONIC EFFETS CAN BE FOLLOWED**

Scheme I. Overall Metabolism of (*S*)-Nicotine to *cis*- and *trans*-Nicotine *N'*-Oxide, Cotinine, and Nornicotine

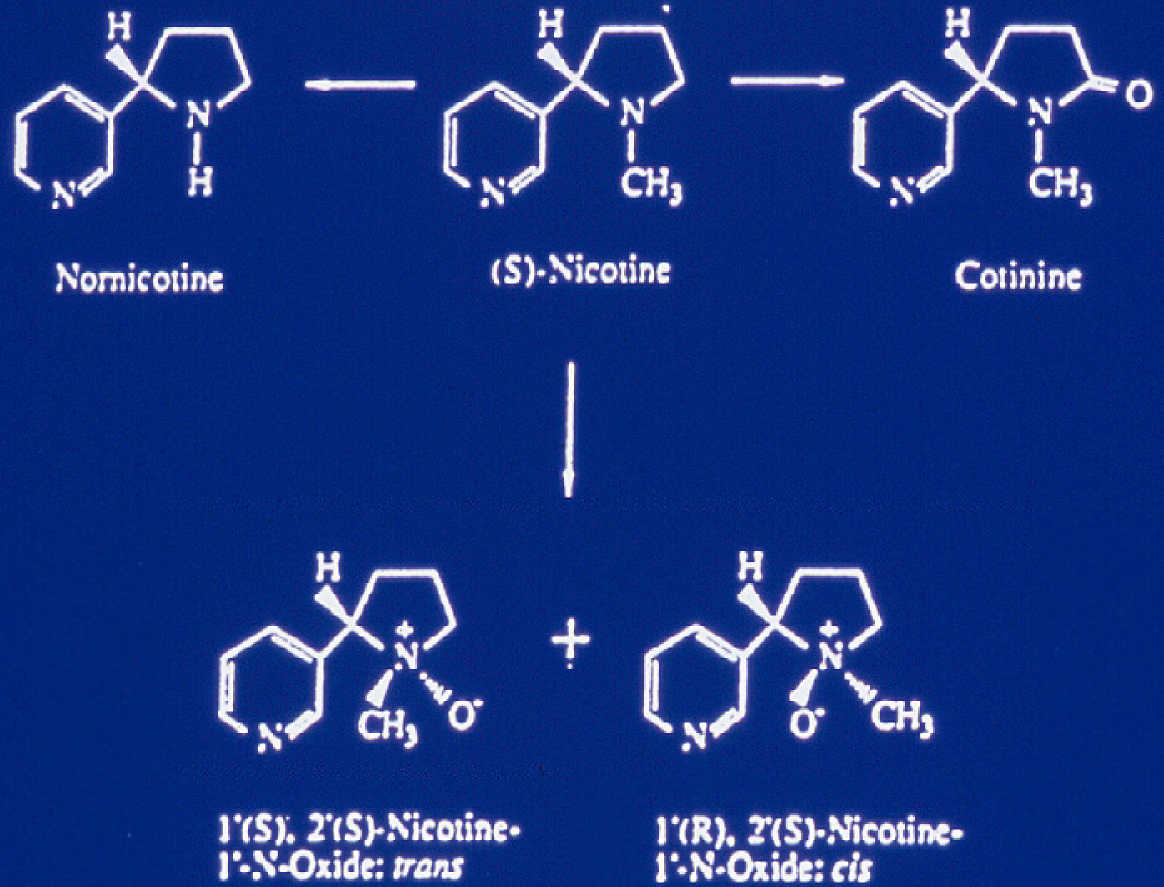

REPORT OF SURGEON GENERAL, 1986

1) NICOTINE IS SIMILAR ON ALL CRITICAL MEASURES TO PROTOTYPIC DRUGS OF ABUSE SUCH AS MORPHINE AND COCAINE

2) NICOTINE PRODUCES A VARIETY OF INDIVIDUAL SPECIFIC THERAPEUTIC ACTIONS SUCH AS MOOD AND PERFORMANCE ENHANCEMENT

3) NICOTINE IS DELIVERED TO CENTRAL NERVOUS SYSTEM IN ADDICTING QUANTITIES AND CIGARETTE SMOKE IS A MAJOR CORRELATE OF OTHER KINDS OF DRUG DEPENDENCE

Whole Animal Studies

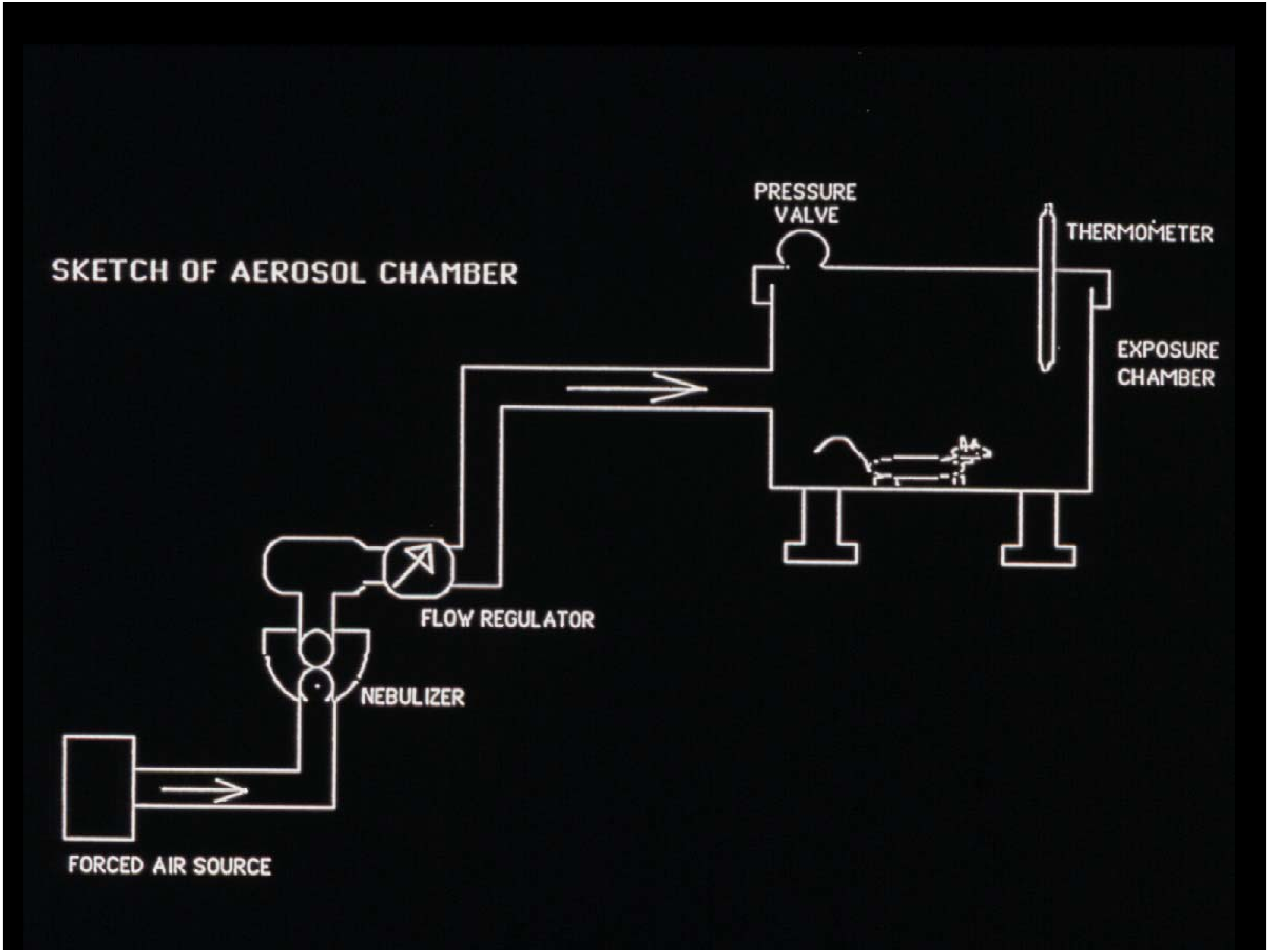
SKETCH OF AEROSOL CHAMBER
PRESSURE VALVE
THERMOMETER
EXPOSURE CHAMBER
FLOW REGULATOR
NEBULIZER
FORCED AIR SOURCE

Schematic of a Newly Designed Aerosol Chamber

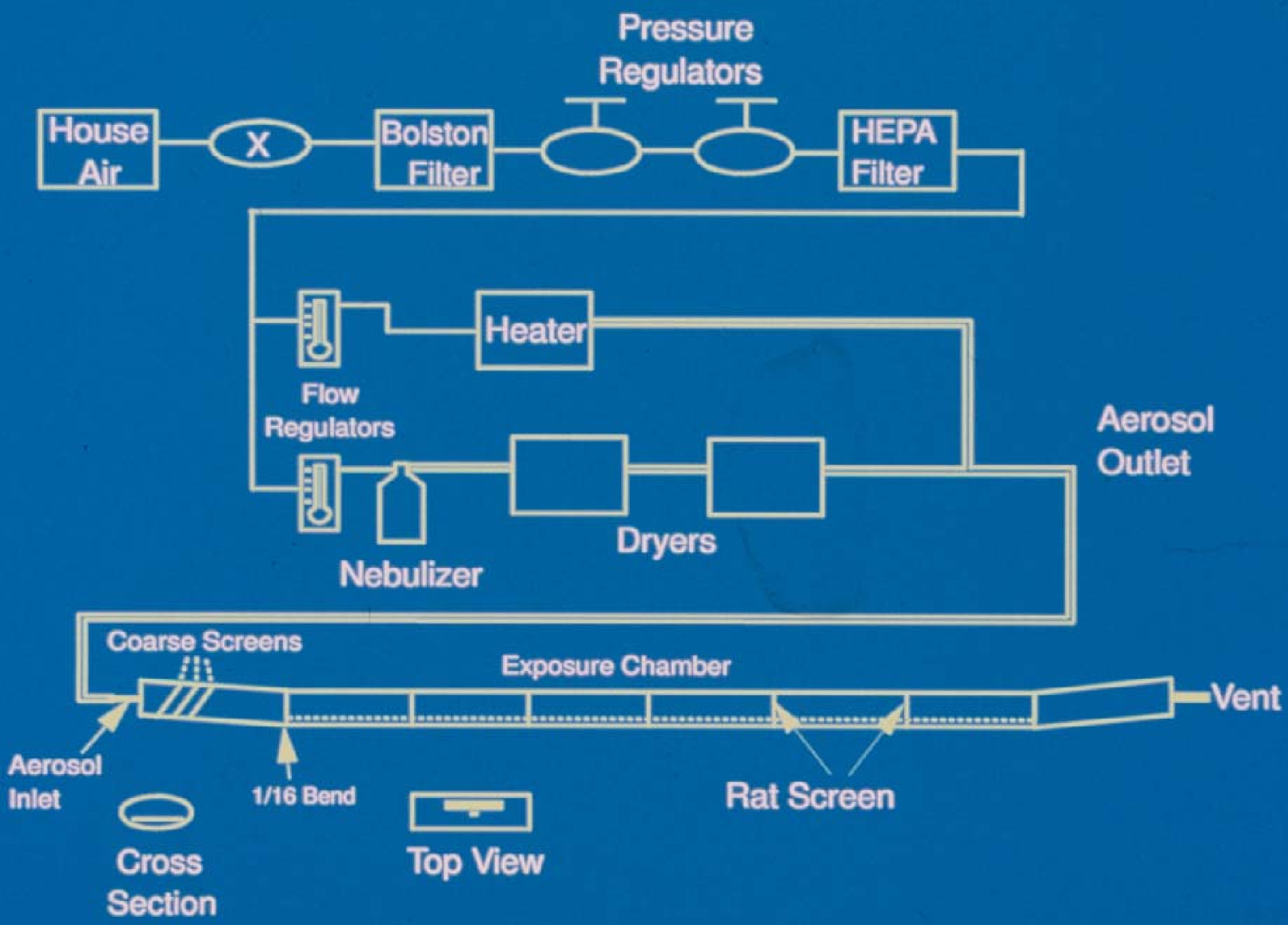

Table I. Plasma Nicotine Levels and Histopathology

Time of exposure (min)	Plasma nicotine (ng/ml)	Extent of pancreatic lesions[a]
15	21 ± 0.6	0
30	37 ± 2.2	+
45	35 ± 1.1	++
60	40 ± 2.2	+++

[a] +, Mild lesions; ++, moderate lesions; +++, extensive lesions.

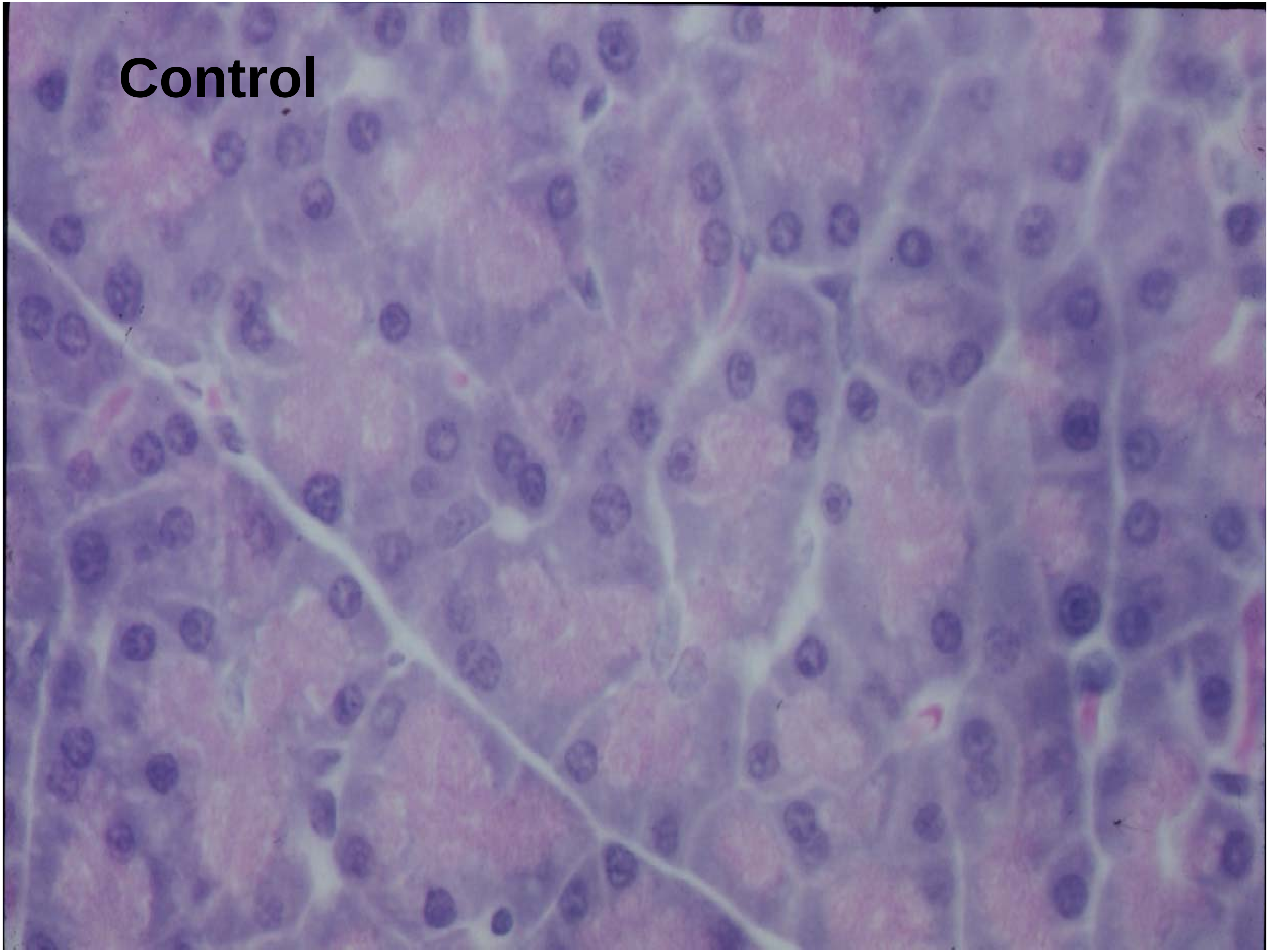
Control

Nicotine Exposed: Fusion of vacuoles and pyknosis

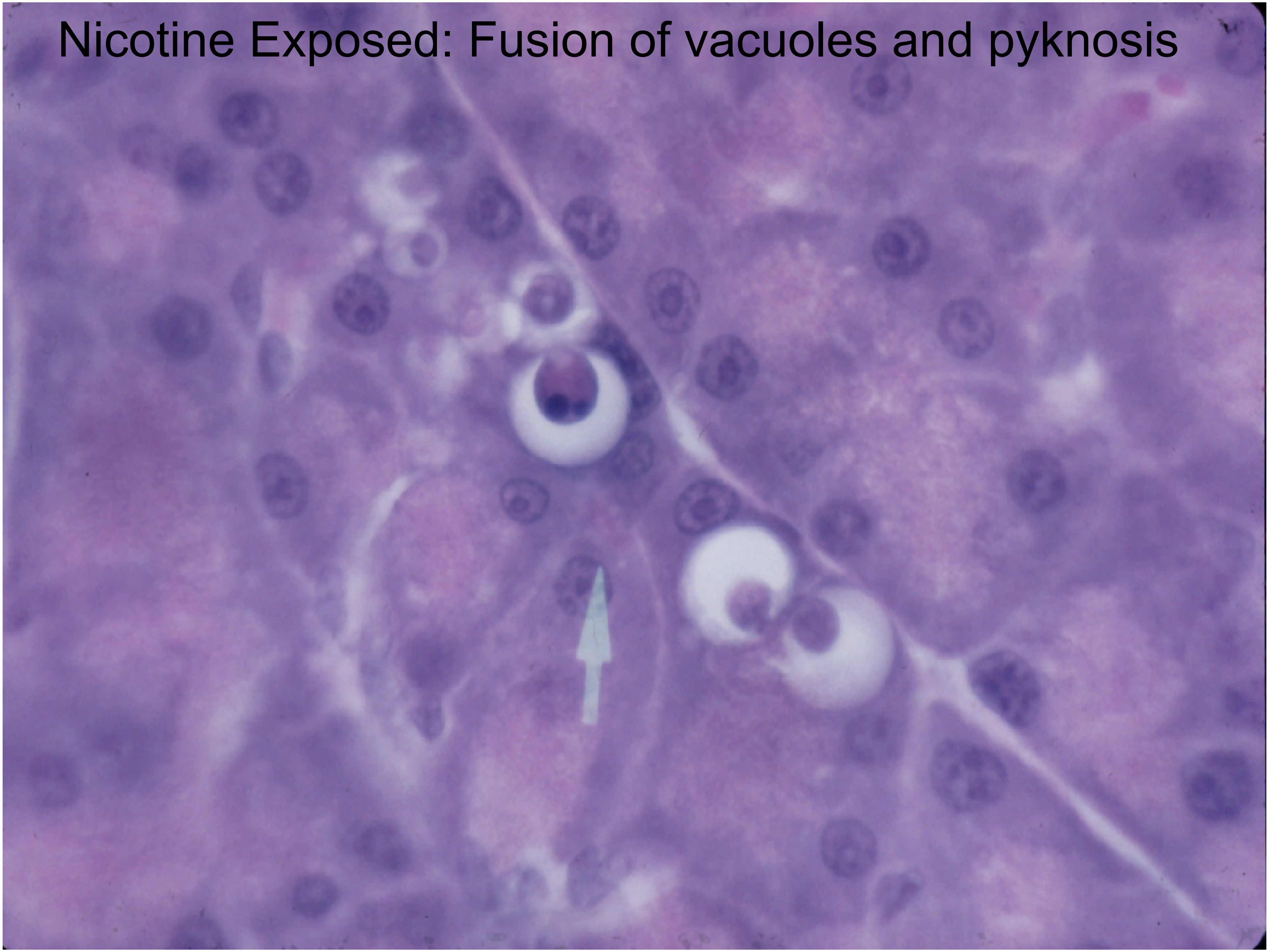

Nicotine Exposed: Cellular Swelling

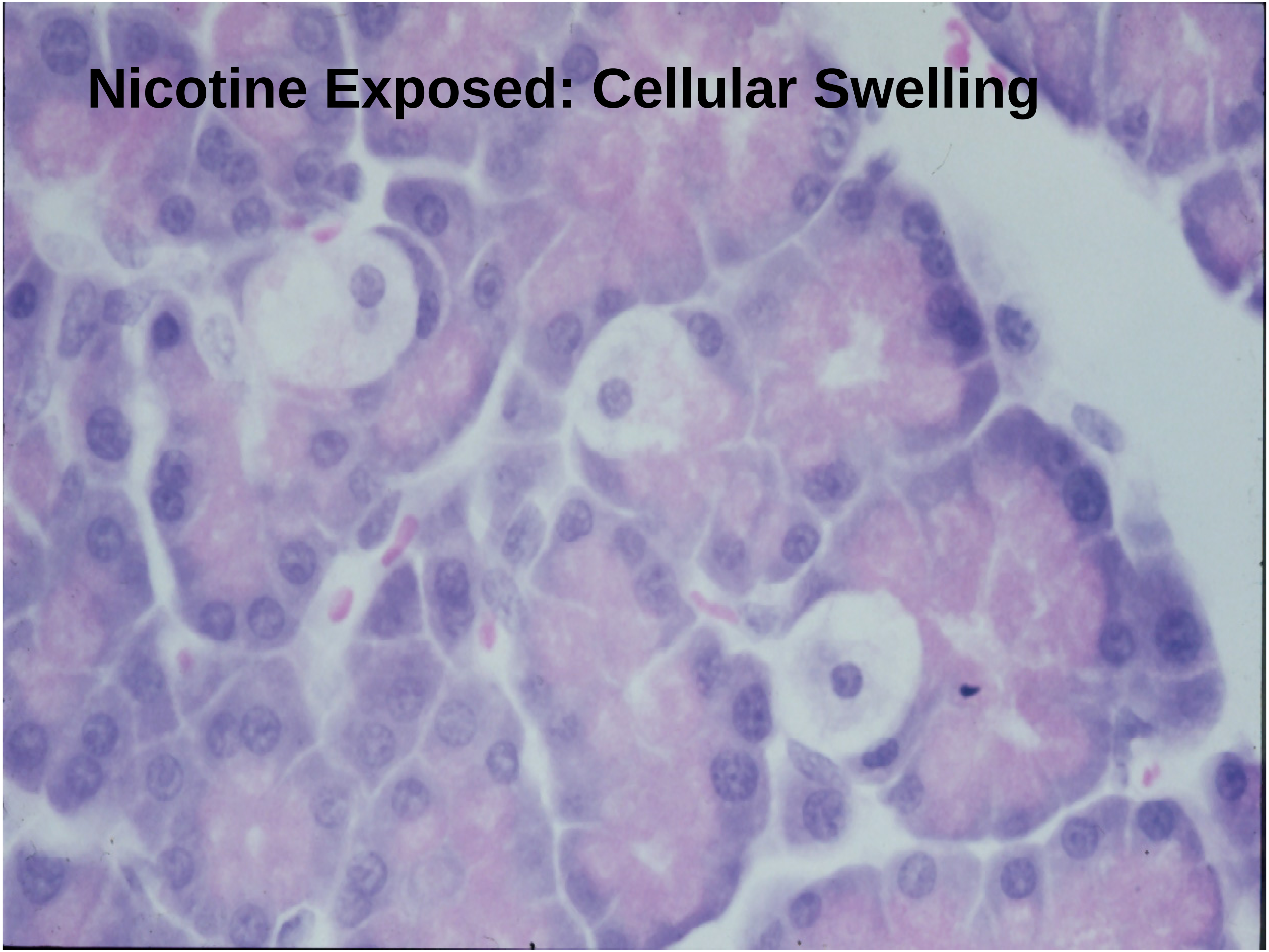

Nicotine Exposed, Collapsed Acini, Transformed Acinar Cells

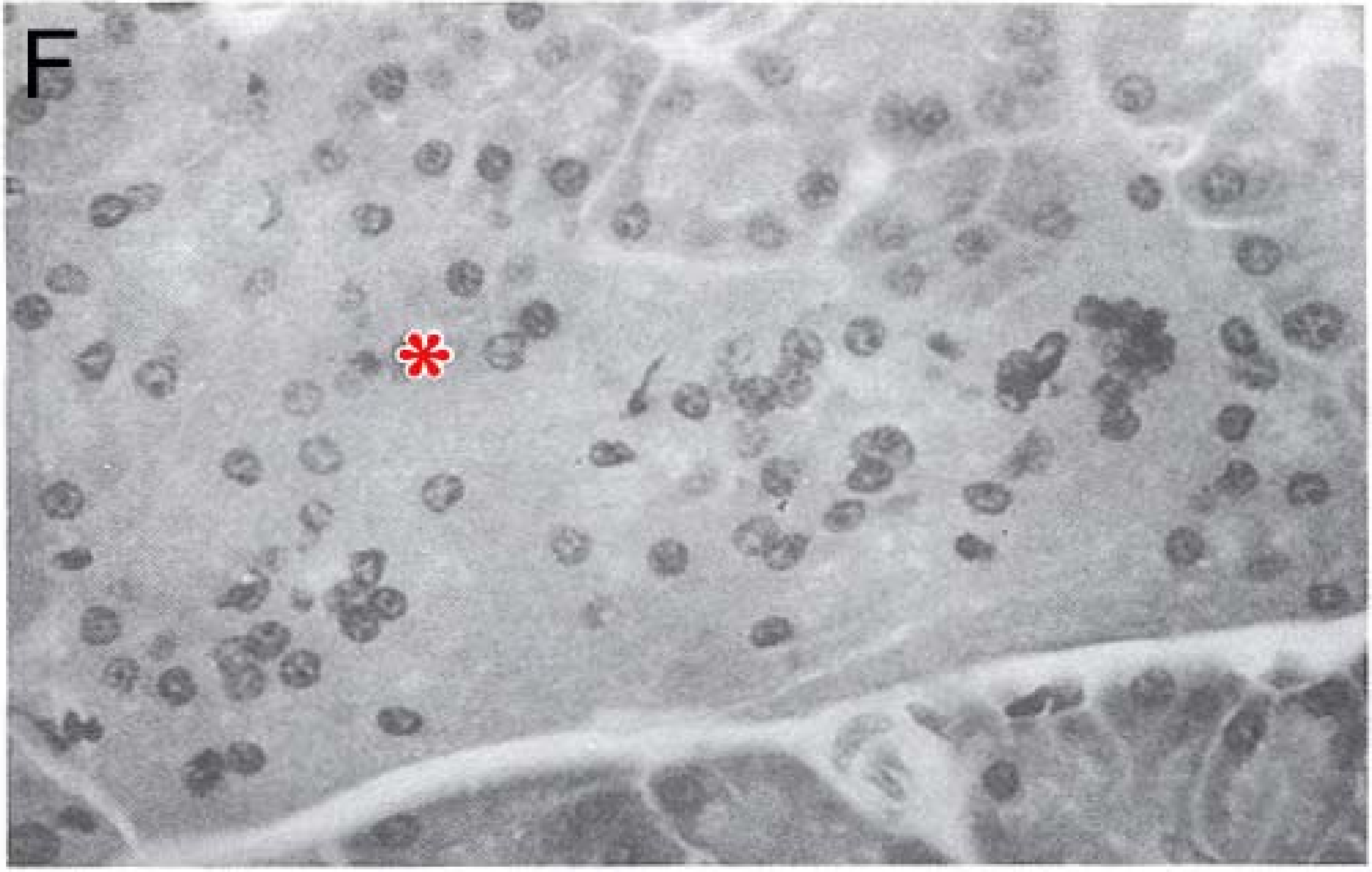

Nicotine Exposed: Large Area Devoid Of Any Acinar Glandular Structures

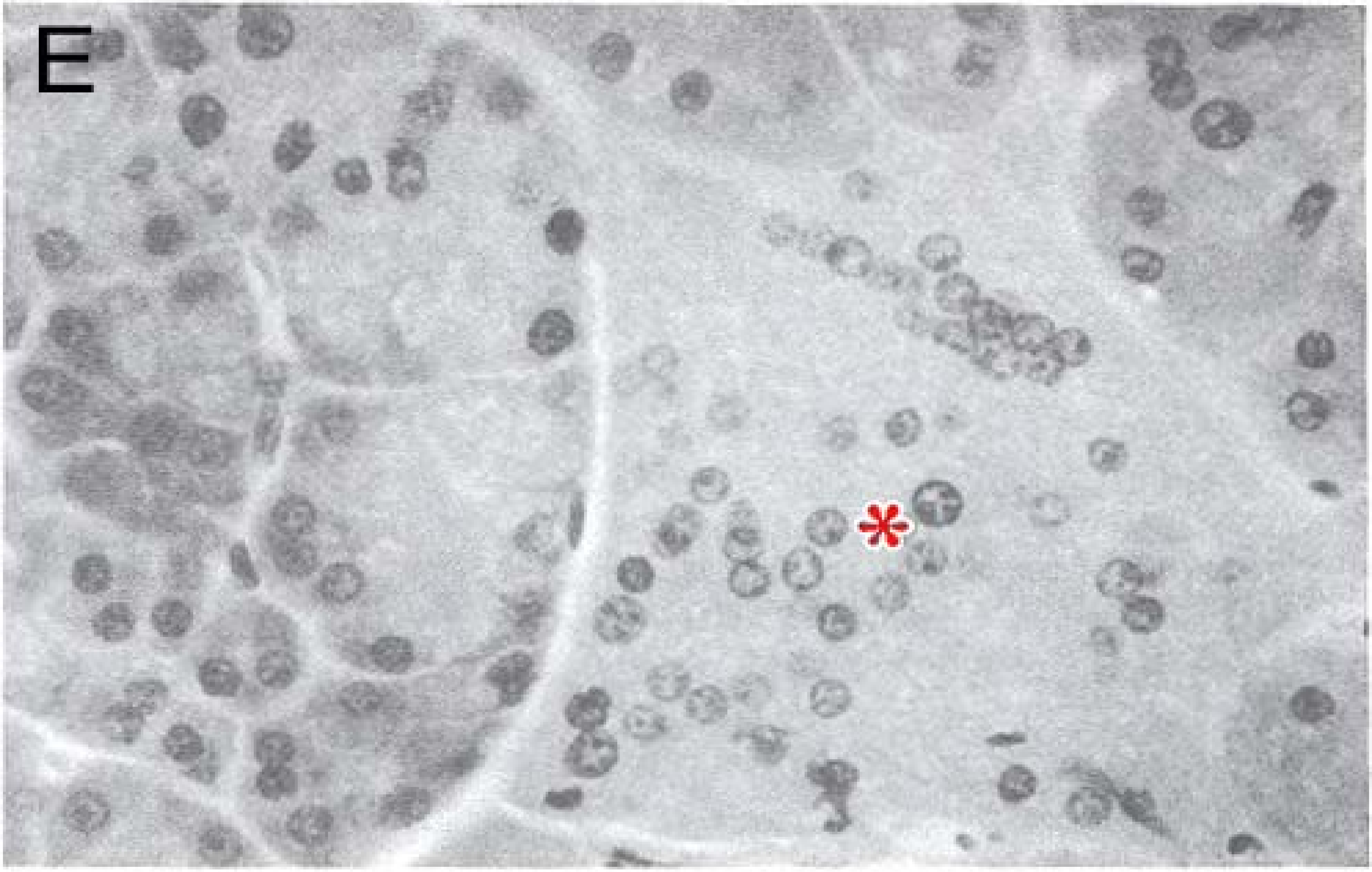

Functional Assay In Isolated Pancreatic Acini

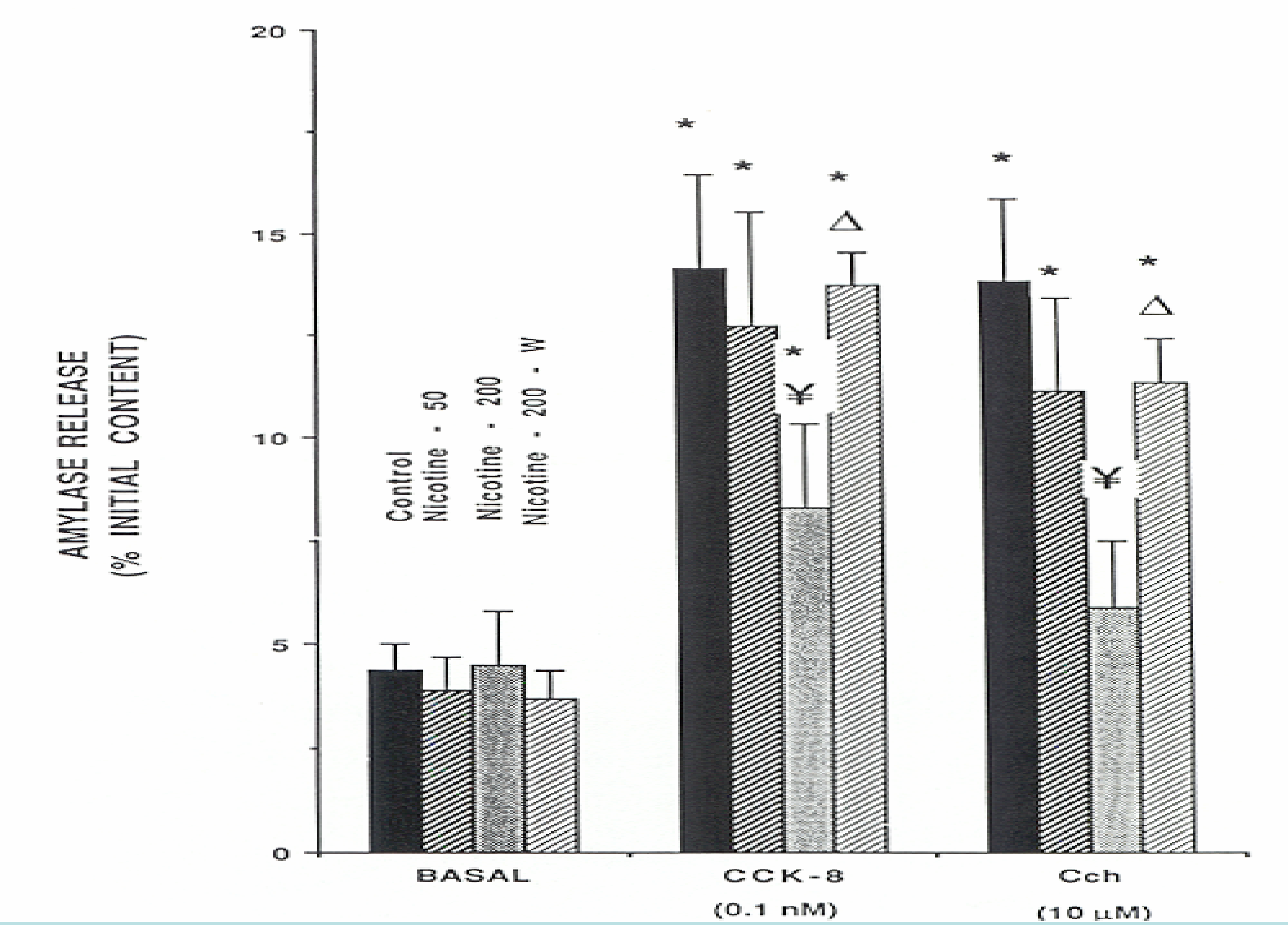

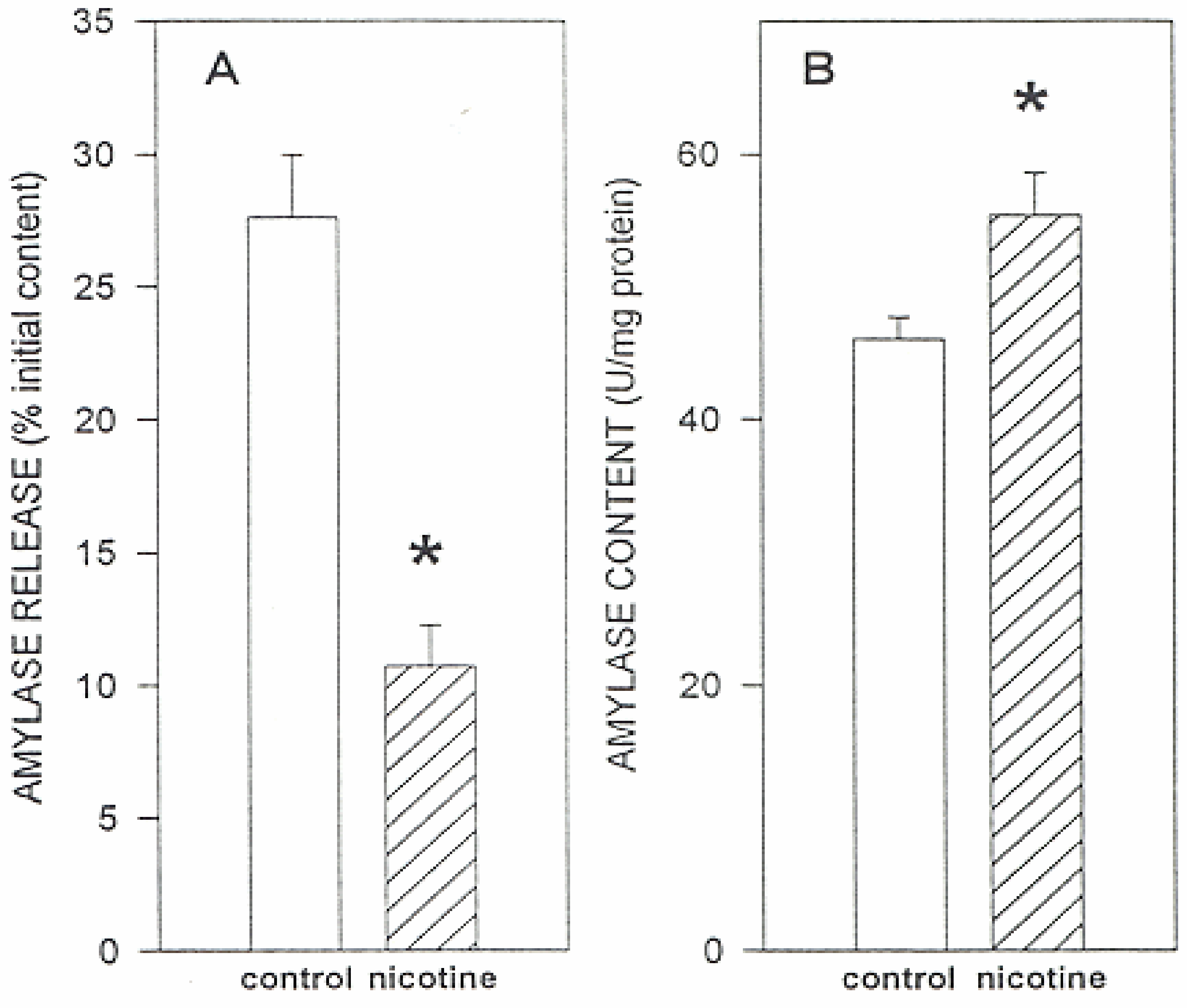

A
B
AMYLASE RELEASE (% initial content)
AMYLASE CONTENT (U/mg protein)
0
5
10
15
20
25
30
35
0
20
40
60
*
*
control
nicotine
control
nicotine

In-vitro studies in pancreatic acinar cells isolated from the untreated control

Amylase Assay

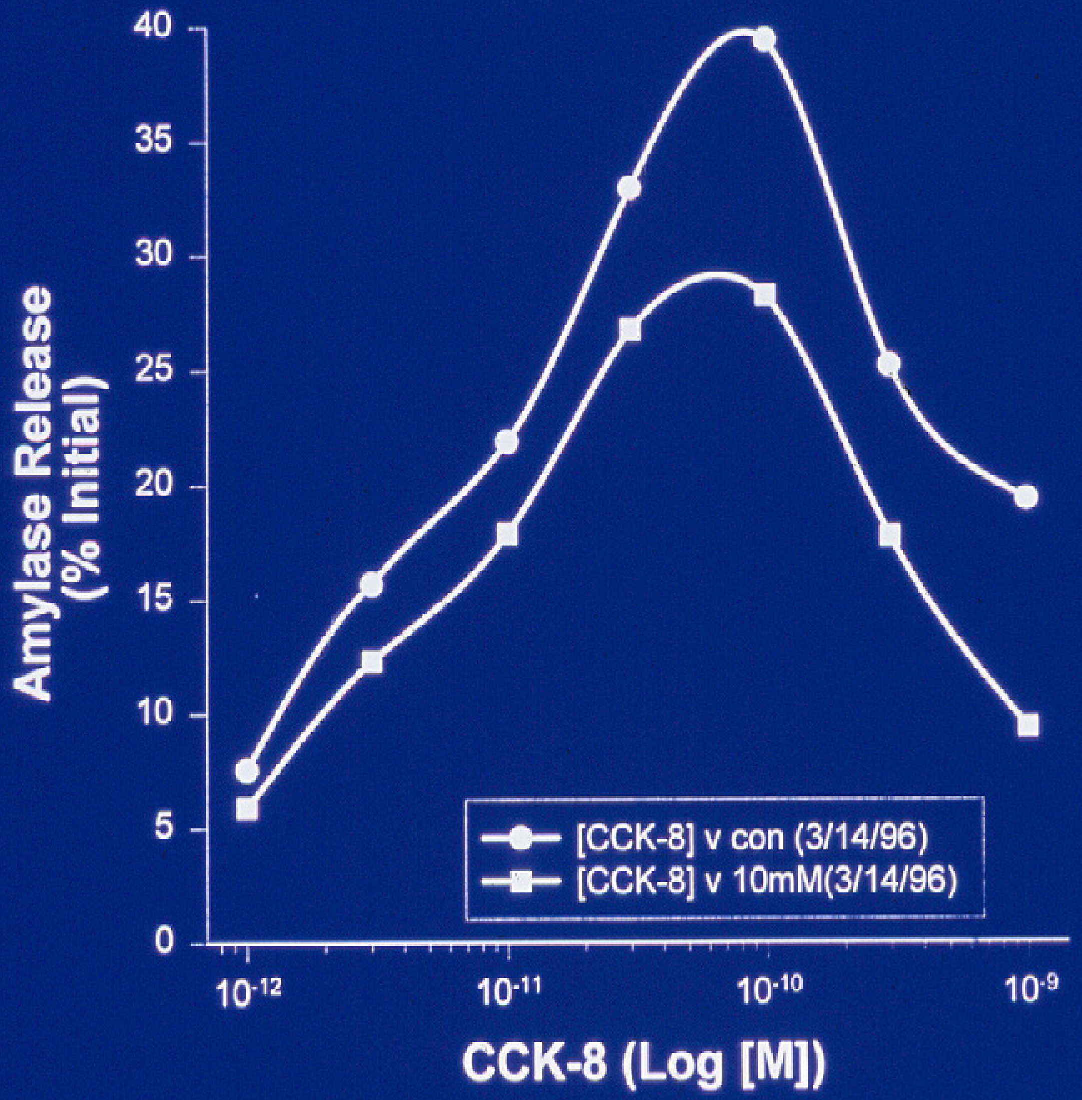

Functional Studies In Cells Isolated From Control And Nicotine Treated Rats

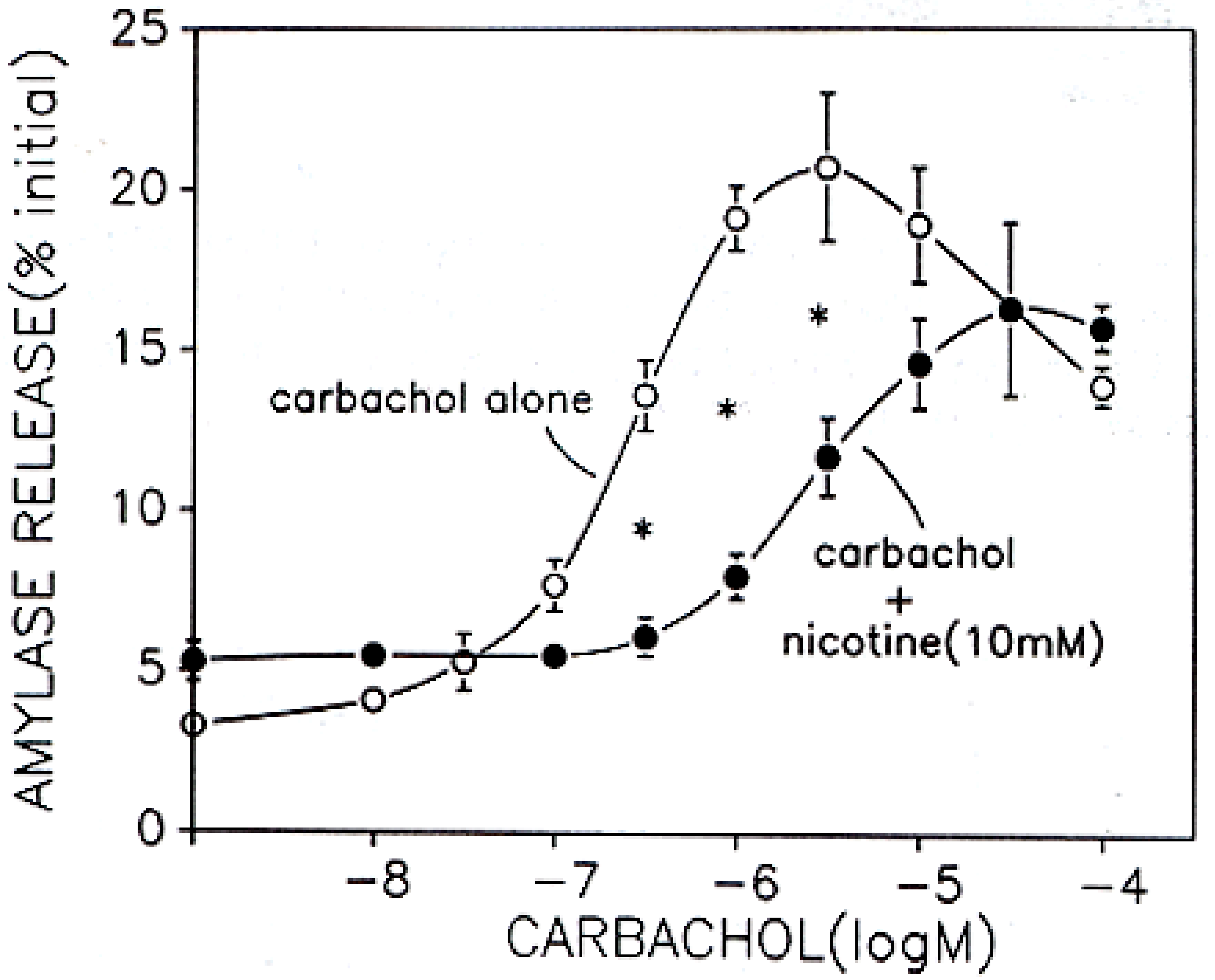

Dose Response Effect Of Nicotine In Isolated Acini

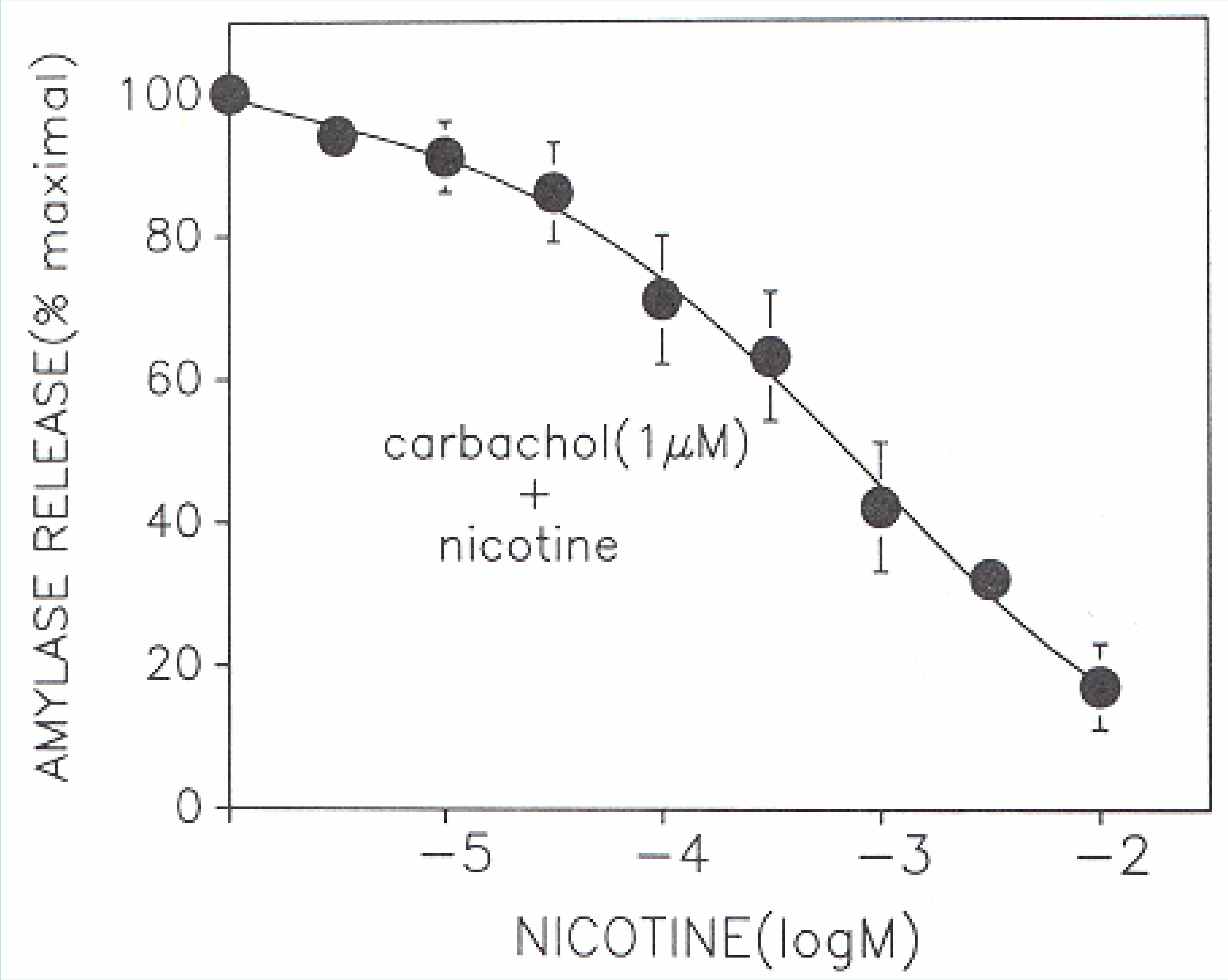

Association And Dissociation Of Nicotine

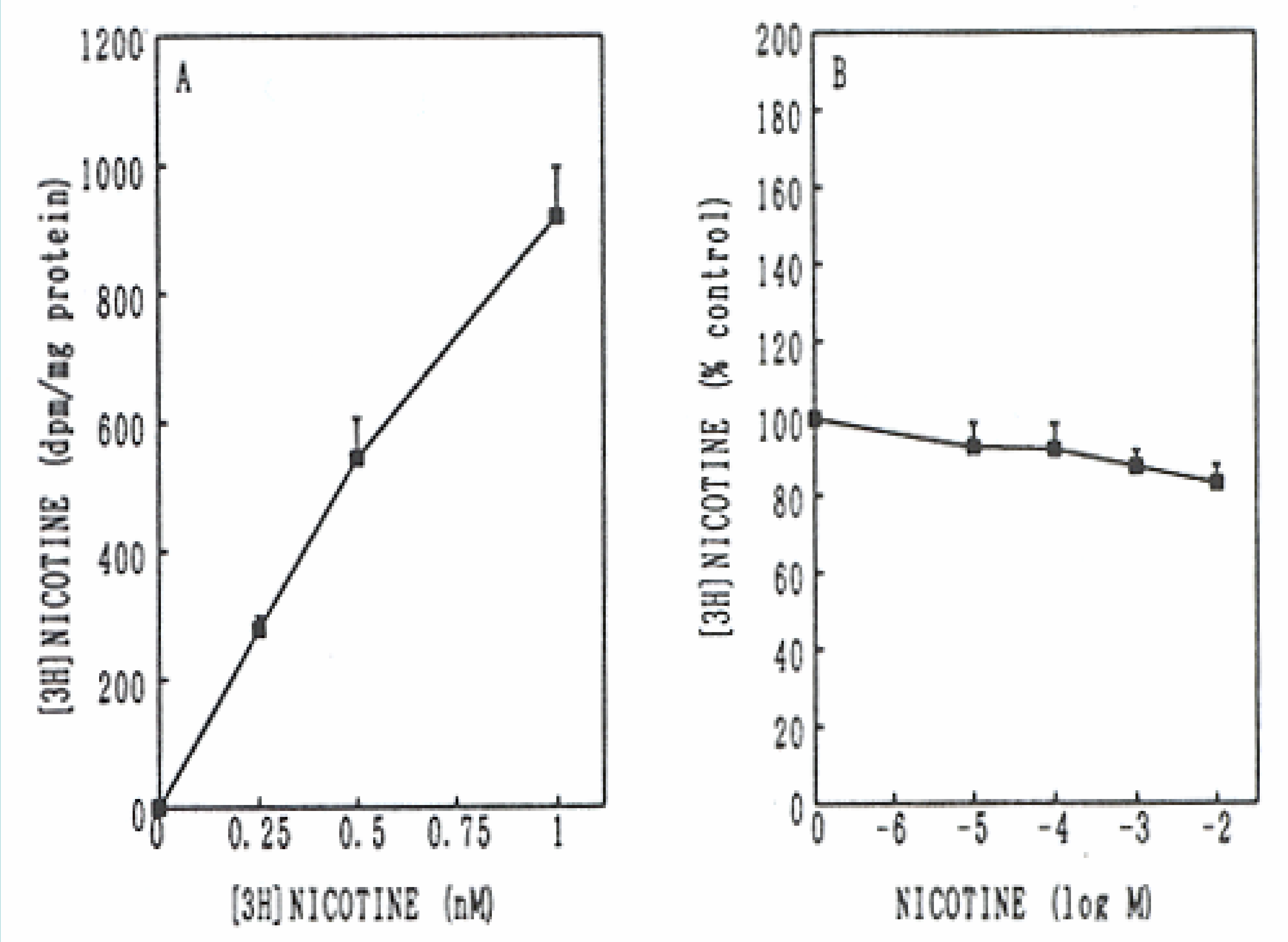

Effect Of Carbachol And CCK On Nicotine Association

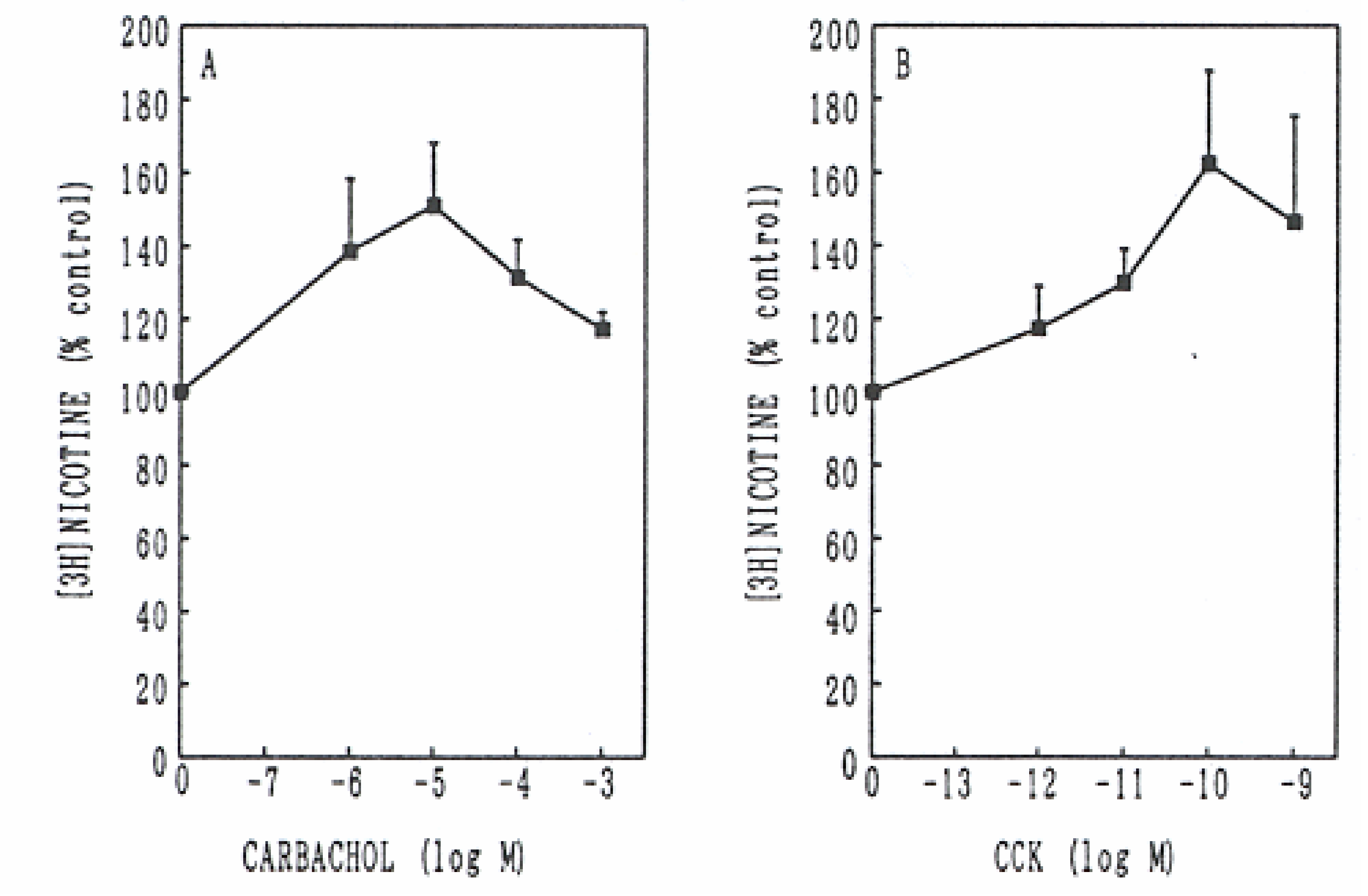

Pancreatic Acinar Cell Model:
Ca^{2+} Regulated Signal Transduction Pathways

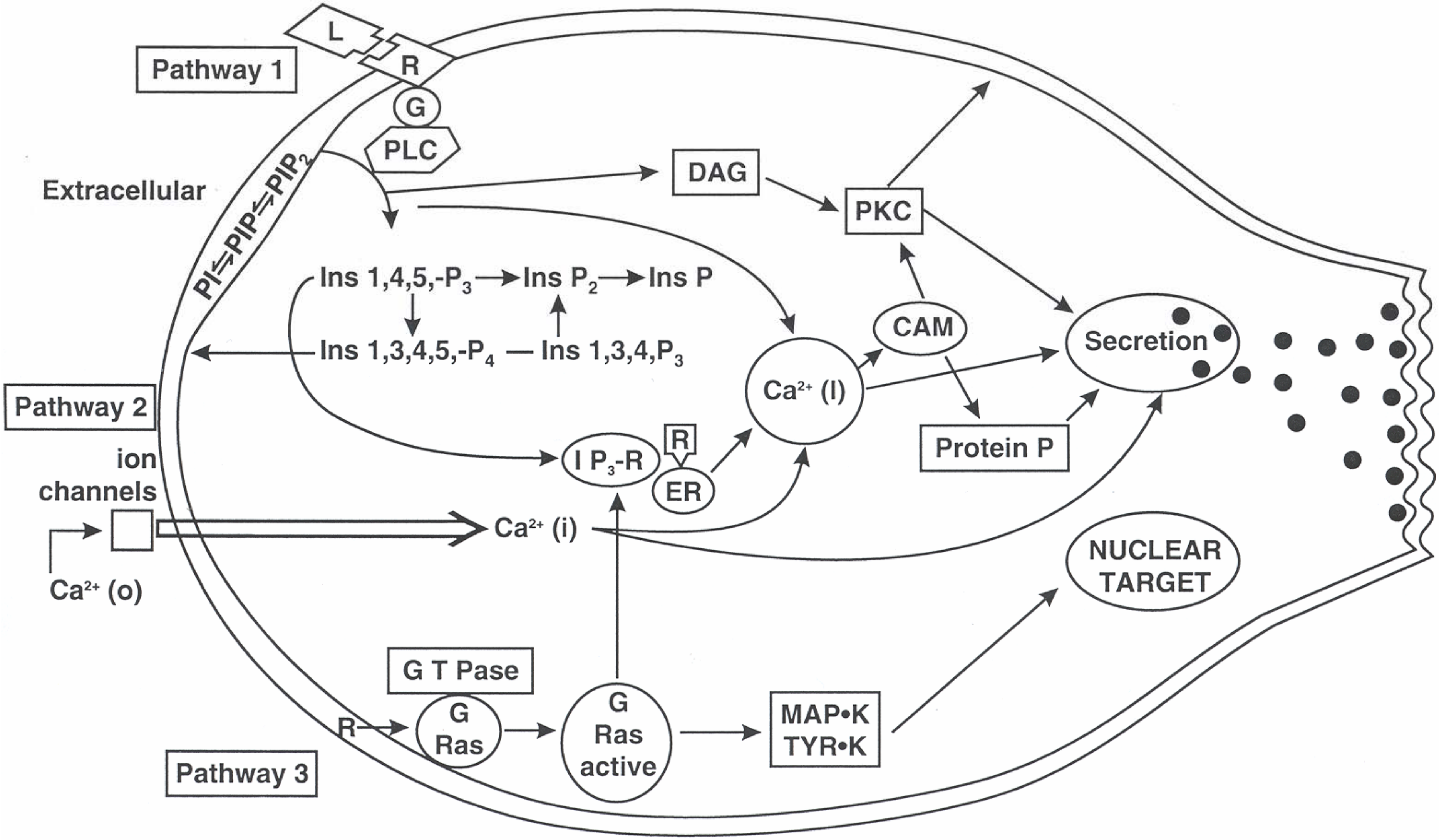

Competitive Binding Studies

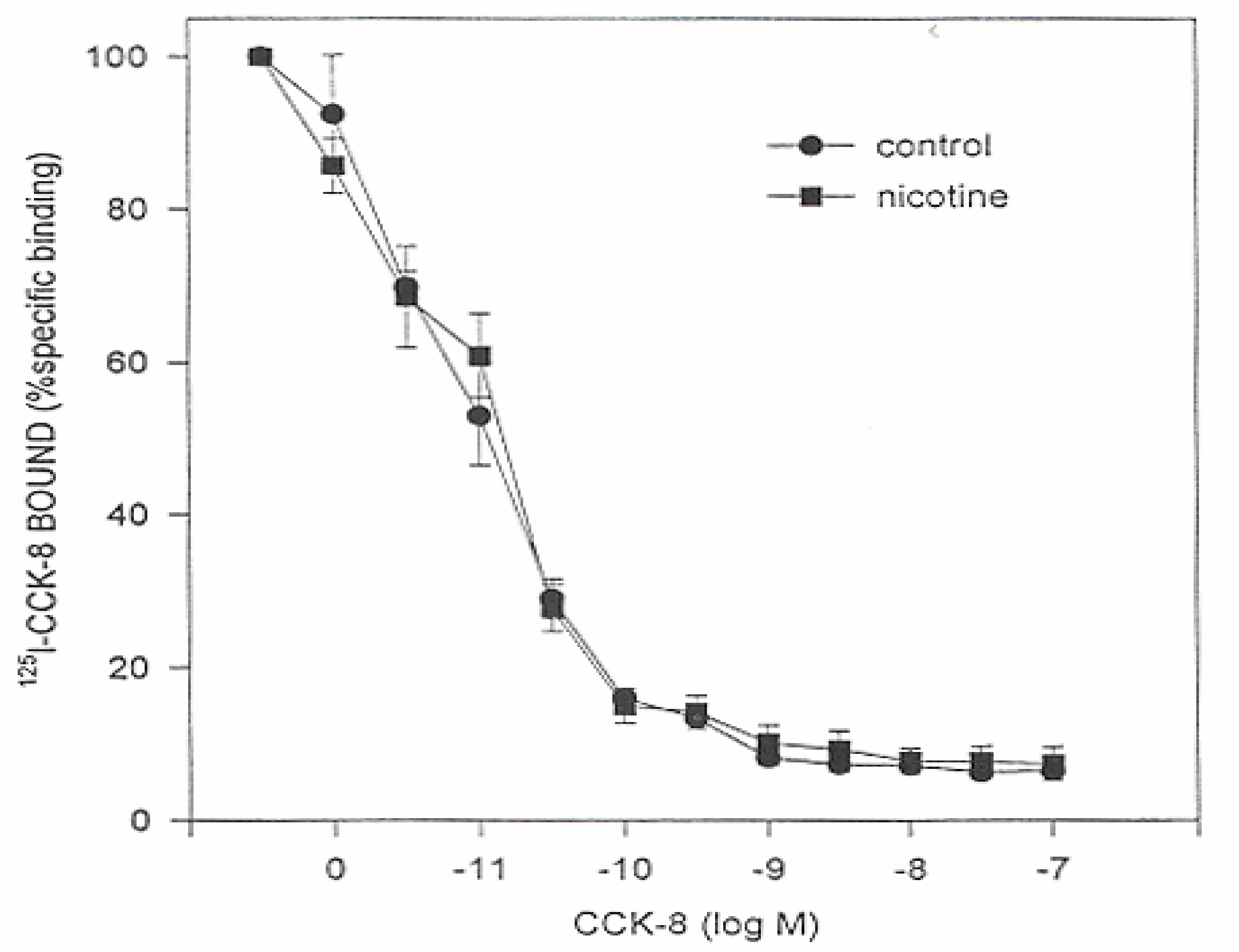

Effects Of Various Agents On Nicotine Association

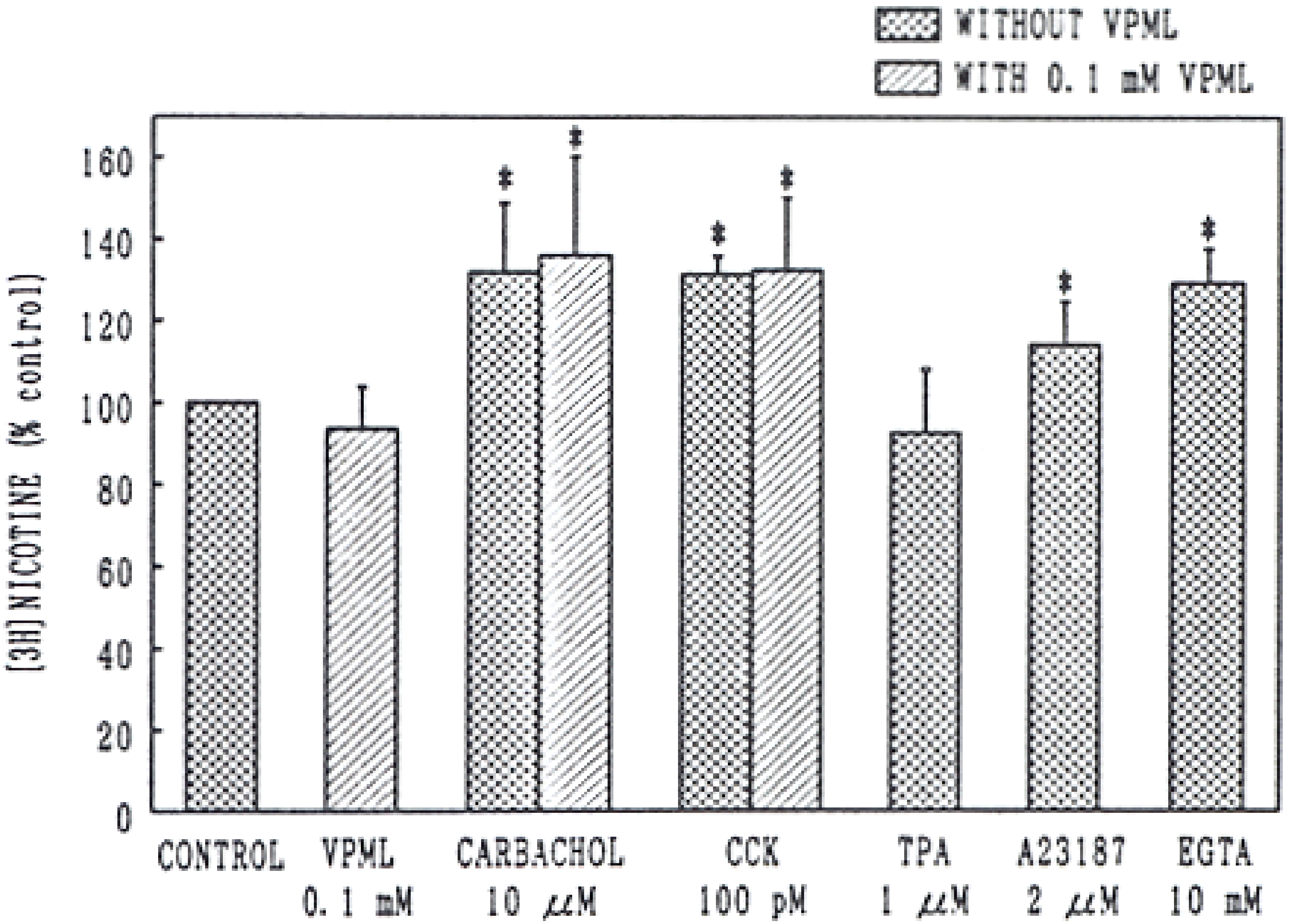

Enhanced Intracellular Ca2+ Fluorescence With Nicotine

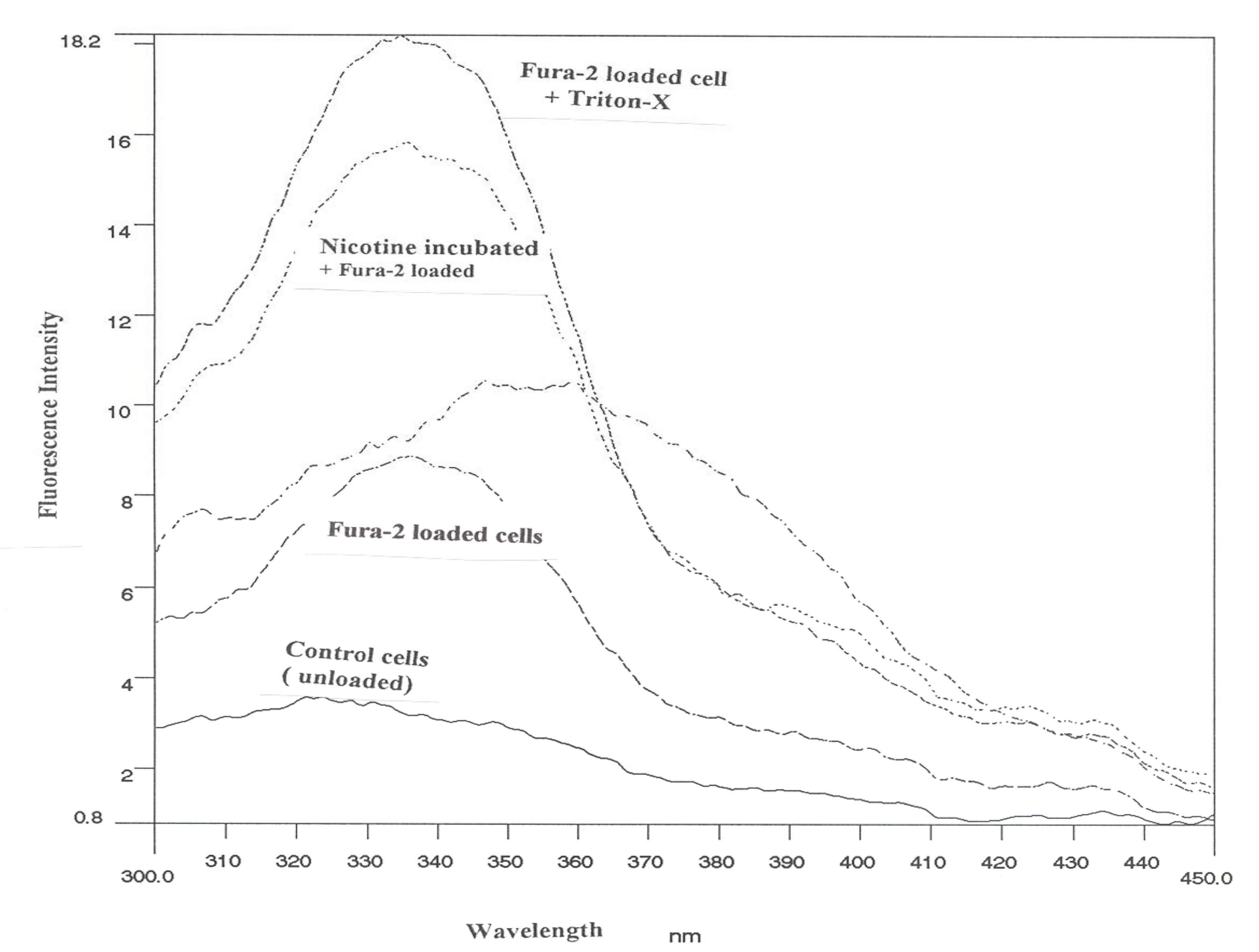

Questions?

How does nicotine enter the acinar cell

Does nicotine exposure induce or express a specific nicotine binding protein

Are there other pathways that may increase intracellular calcium levels that result the cytotoxic effect

Pancreatic Acinar Cell Model:
Ca^{2+} Regulated Signal Transduction Pathways

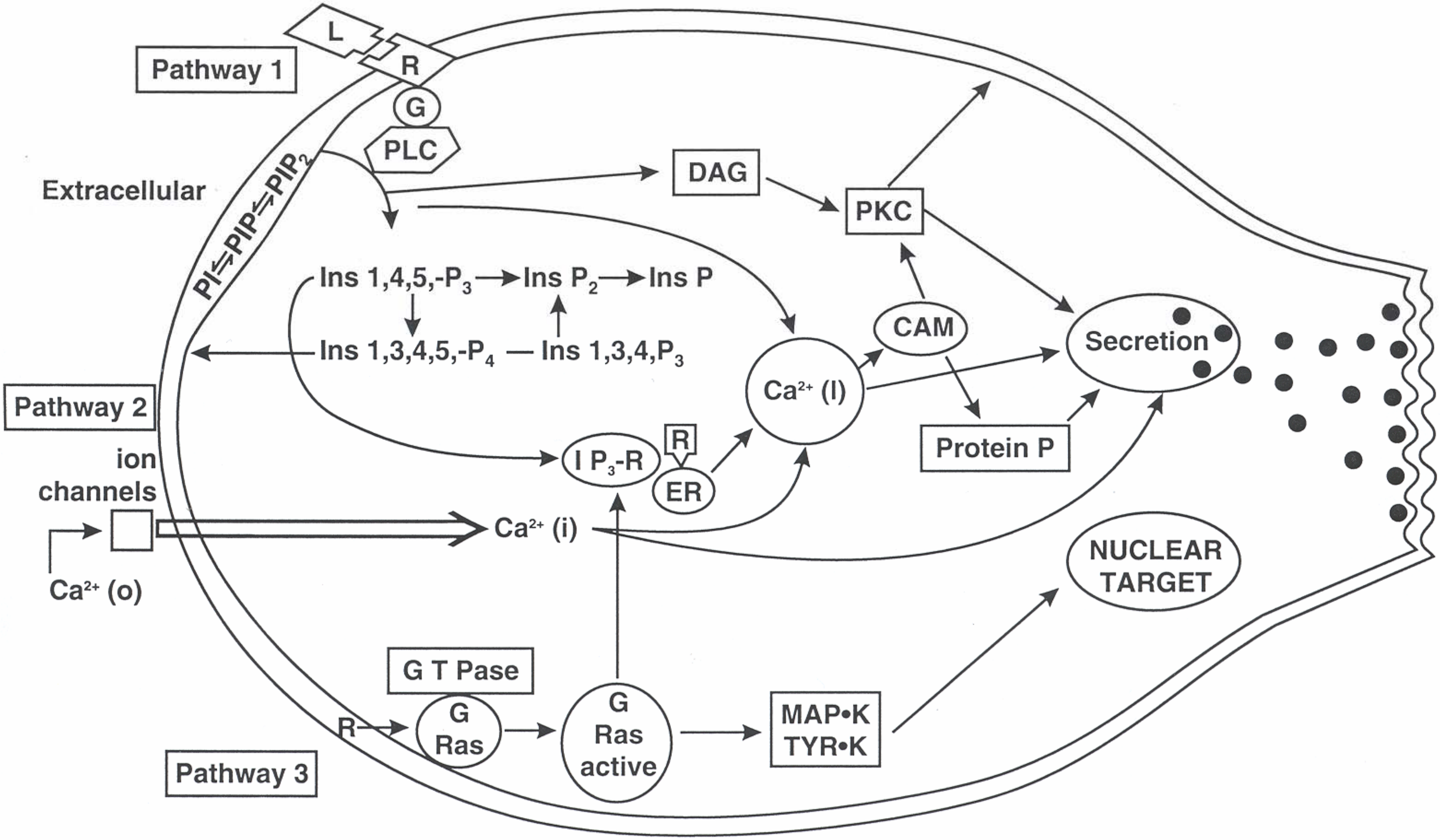

Chromatographic Profile Of Acinar Cytoplasmic Extracts

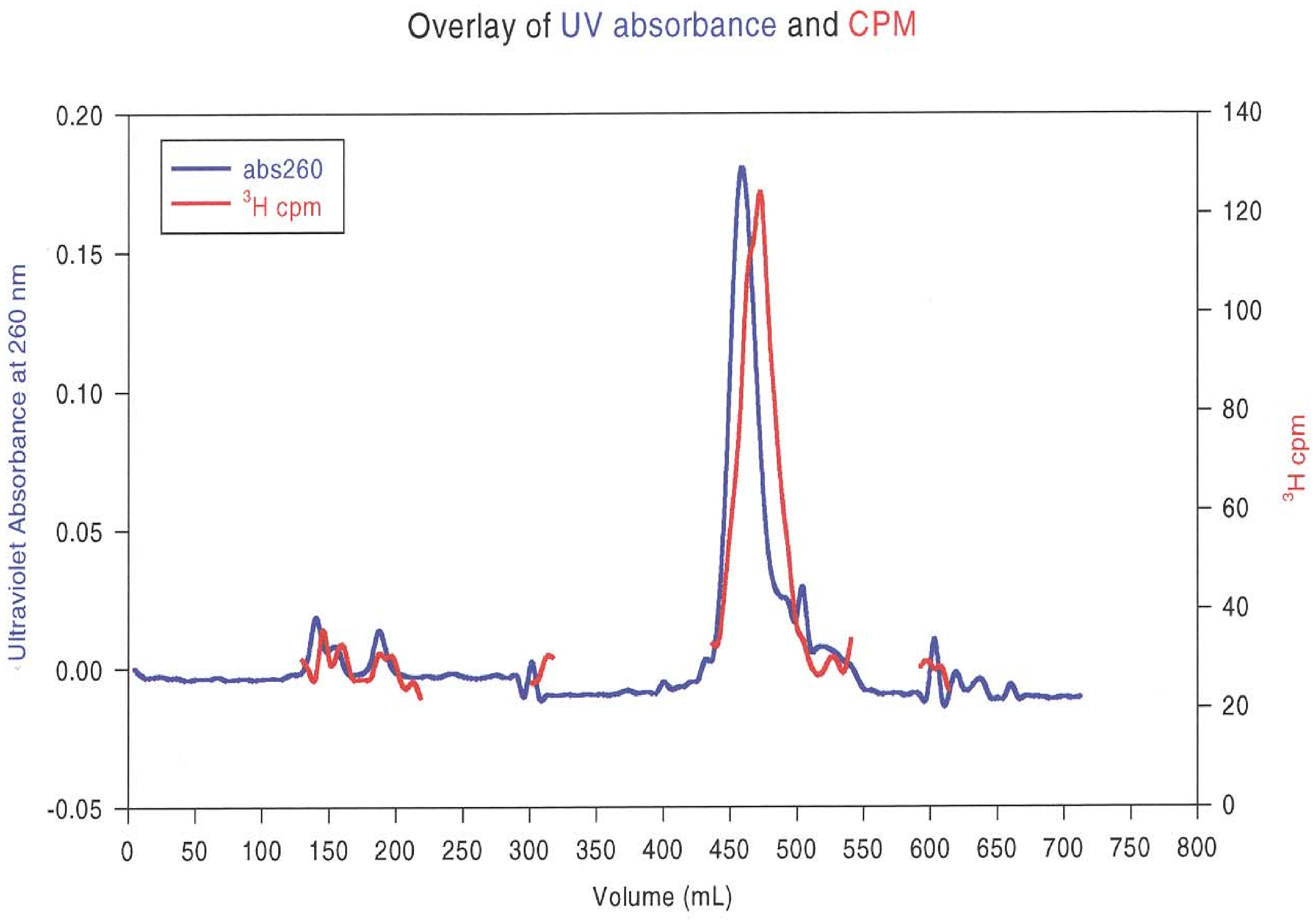

SDS PAGE Of Acinar Cytoplasmic Extracts

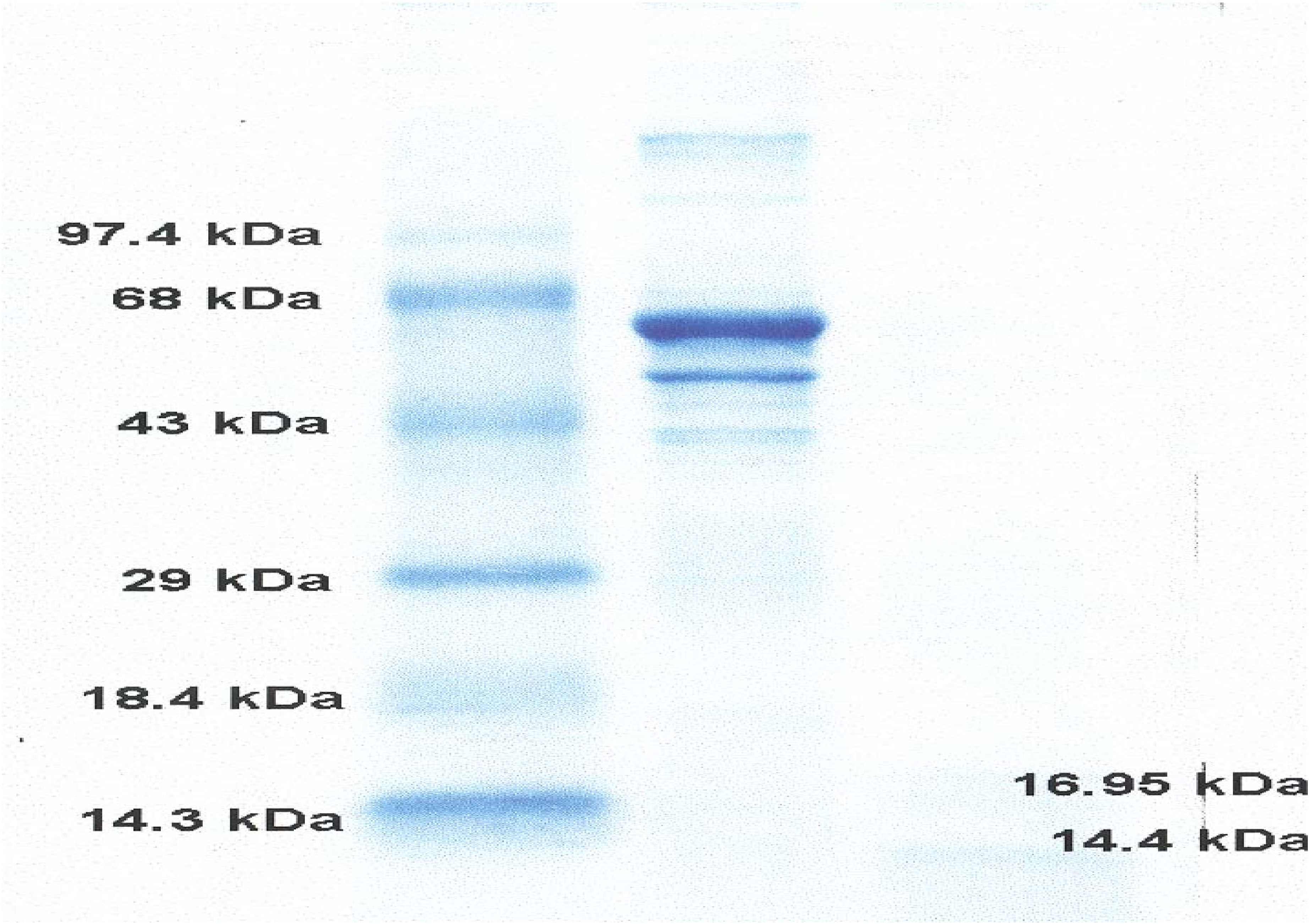

SDS PAGE Of Peak Extracts From G-200 Column

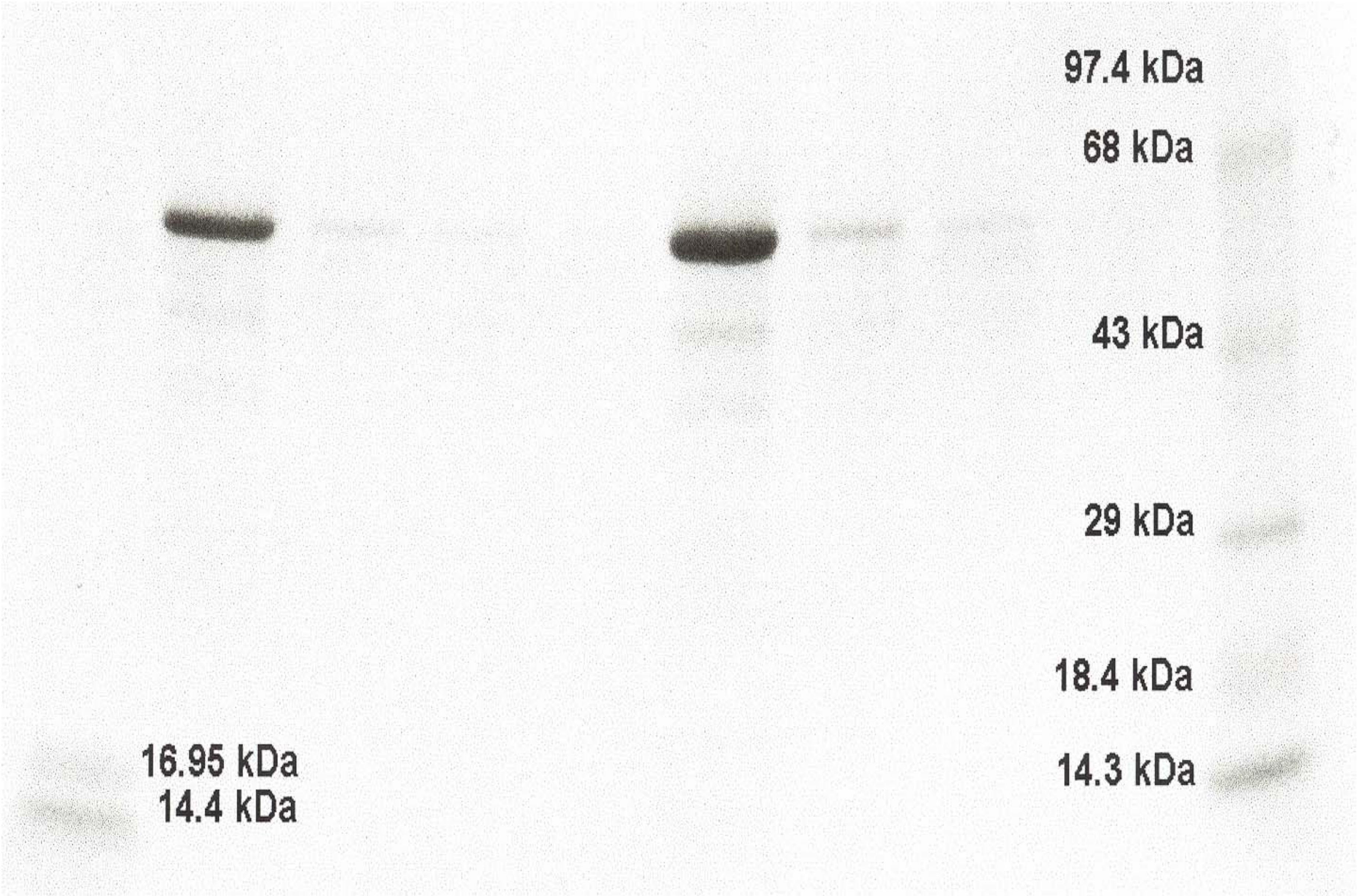

Activation Of Protooncogene, H-ras By Nicotine, Gender Differences

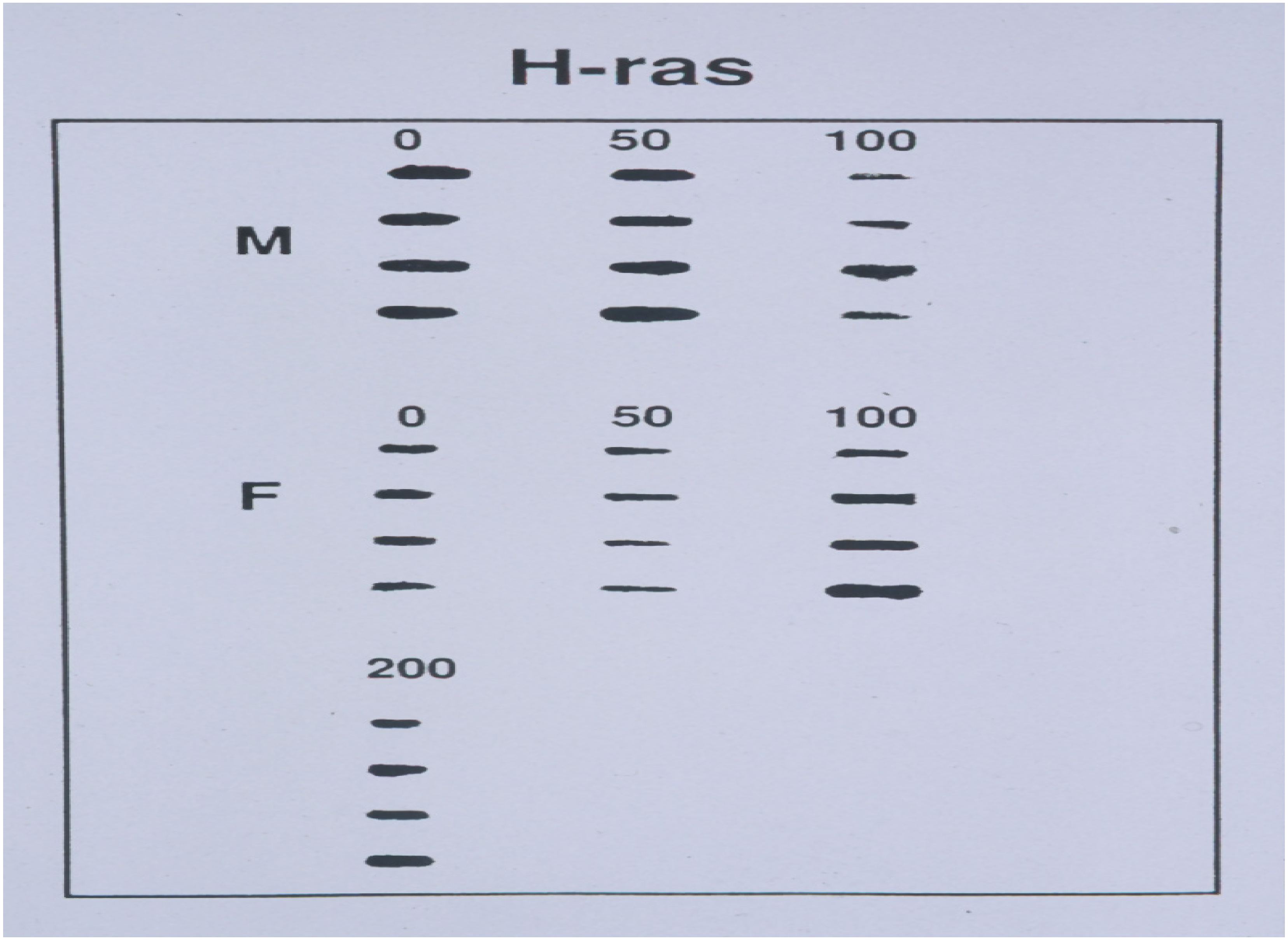

Point Mutation At Codon 61 Of H-ras Protooncogene

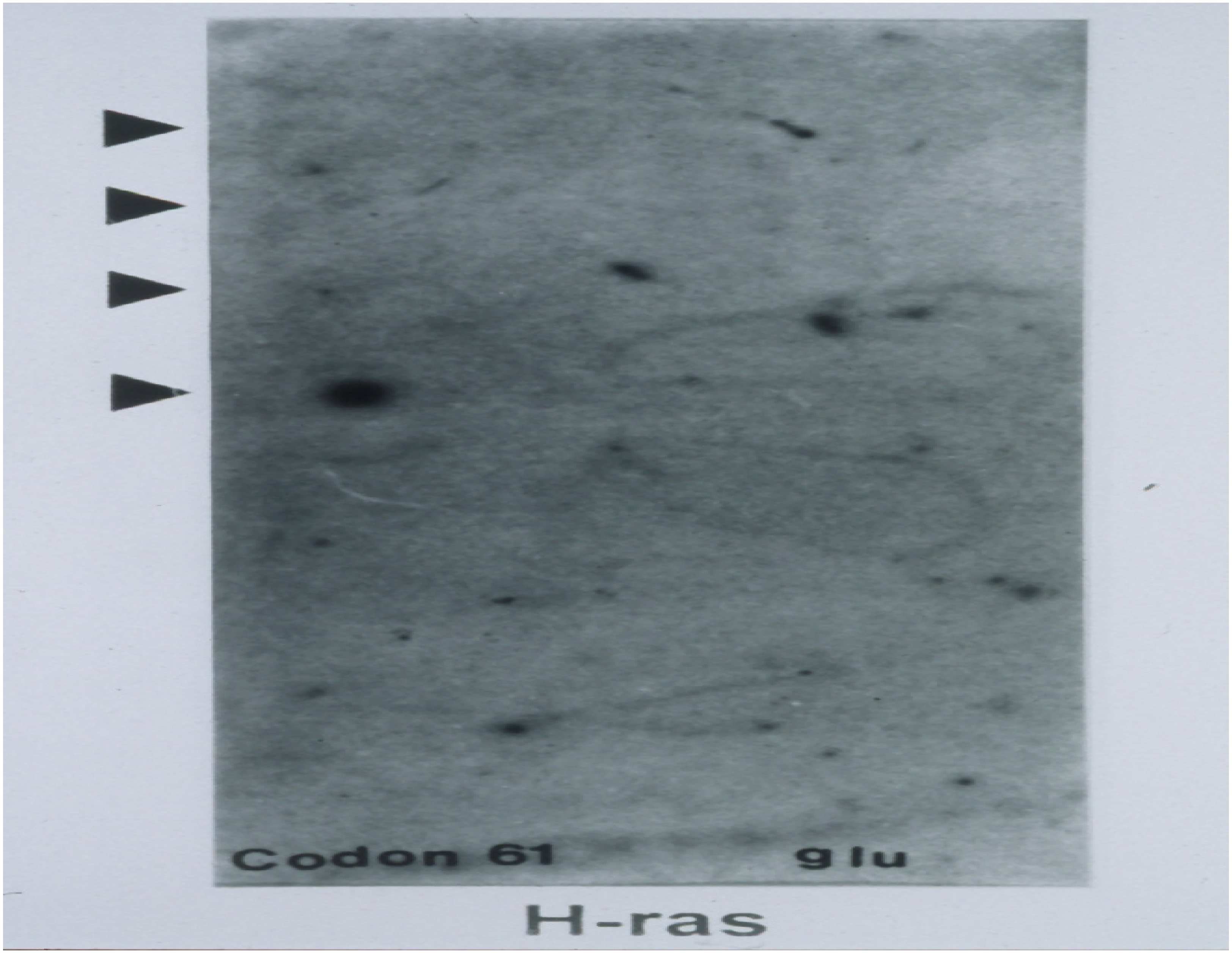

Summary

In vivo and in vitro studies conducted in rodent model showed both ultrastructural and functional correlation in terms of vacuolar change, pyknosis, swelling of exocrine pancreas and a significant loss of pancreatic function

Mechanisms relating to these changes appear to involve altered intracellular calcium regulation

Although the presence of a cytoplasmic nicotine binding protein is partially characterized, its role in this mechanism remain unknown

Summary Continued

The data from these studies appear consistent with the observations reported in patients with acute pancreatitis

The rodent may thus, be considered as a potential model for induction of acute and chronic pancreatitis

Once established the model can explore further the transition of acute pancreatitis to pancreatic carcinogenesis

Studies in AR42J cells, an immortalized rat pancreatic tumor cell line

Low Dose Nicotine Enhanced Tumor Cell Proliferation In Cell Culture

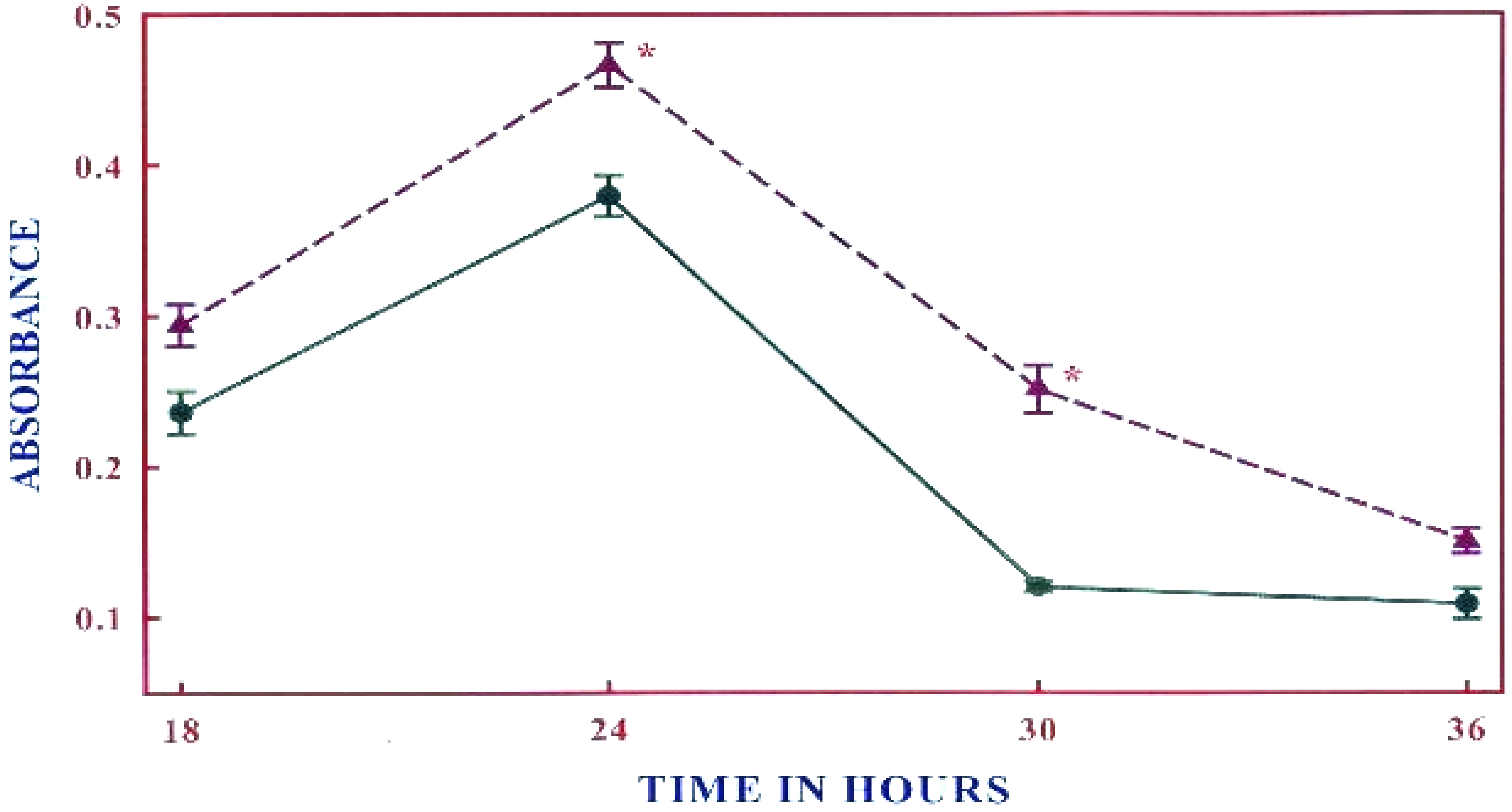

Tumor Cell Proliferative Status With Nicotine (100um) Exp. For 3 Min

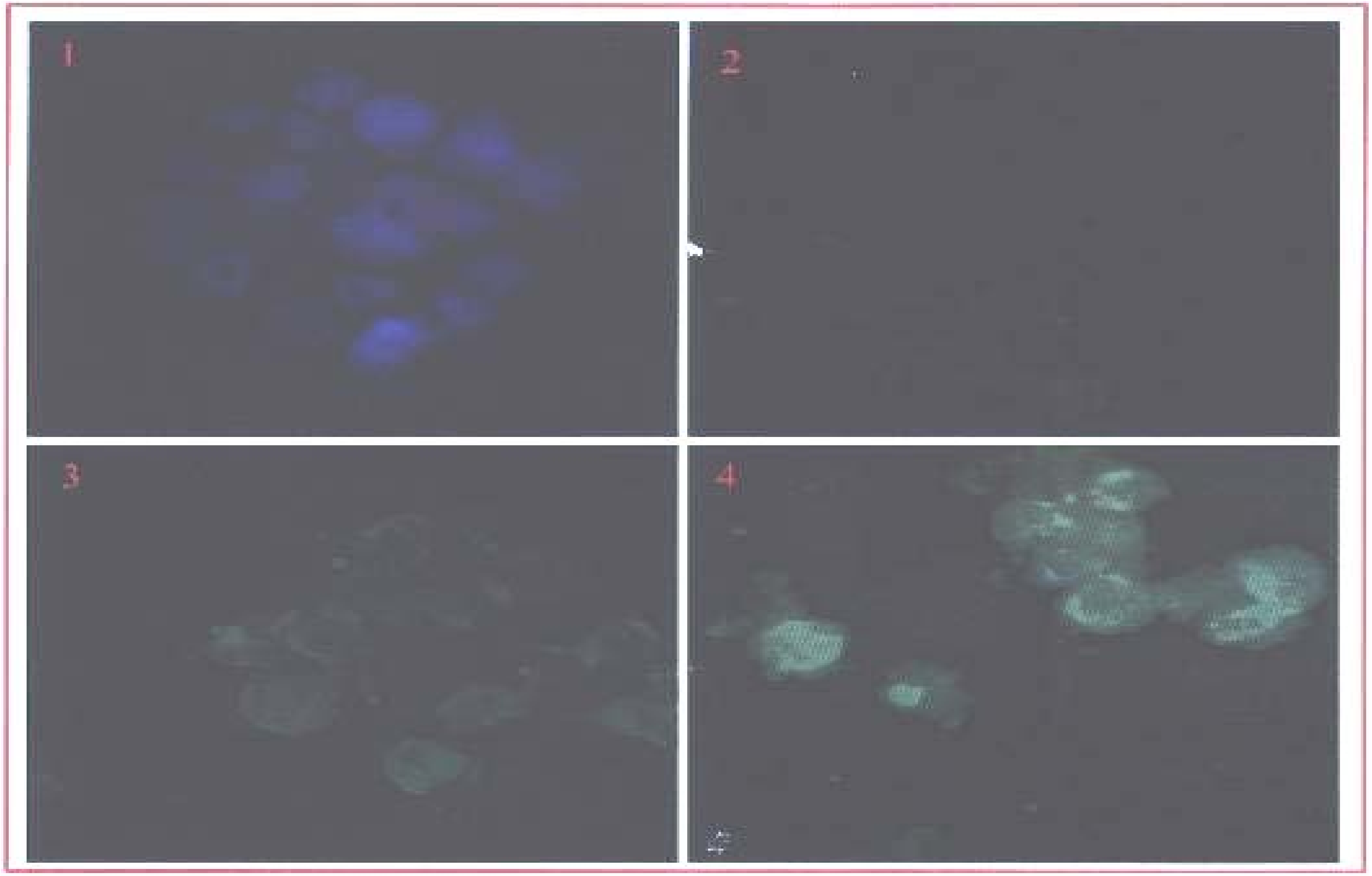

Dose Response Of Nicotine On ERK Activation In AR42J Cells

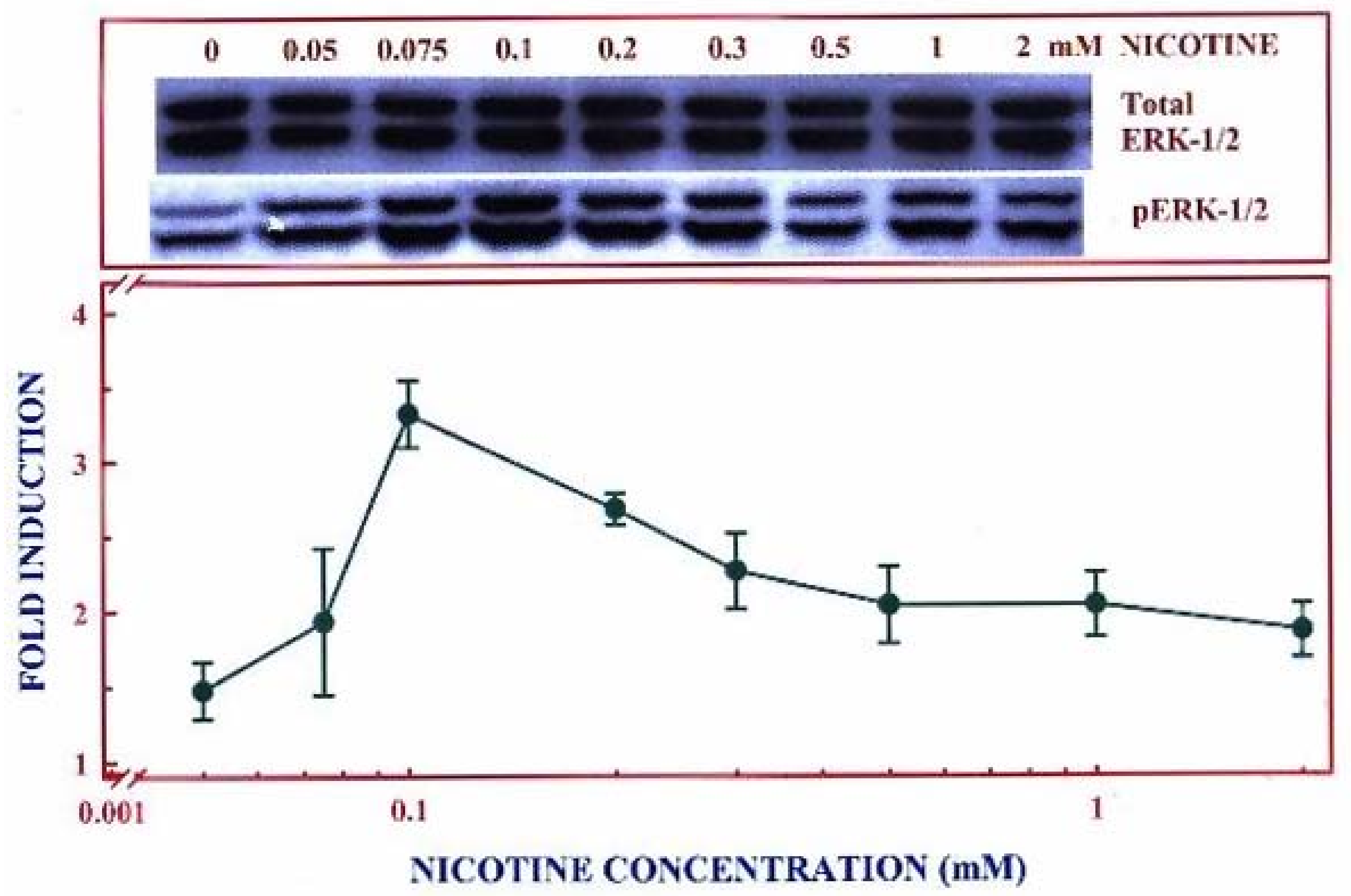

Maximal Activation Of P-erk Occurred In 3 Min Of Nicotine(100um) Exposure

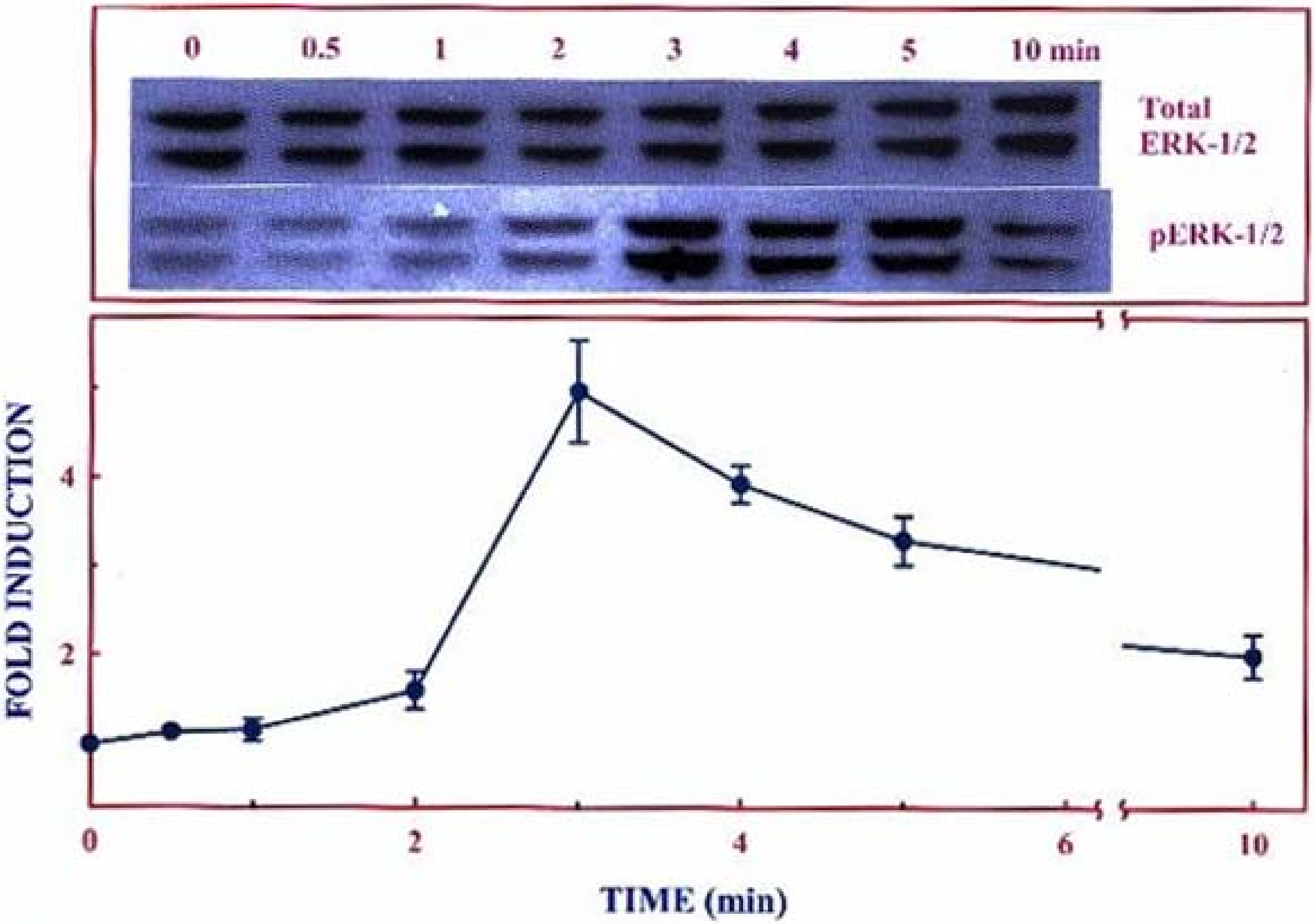

Effect Of ERK Inhibitors On Nicotine Induced ERK Activation

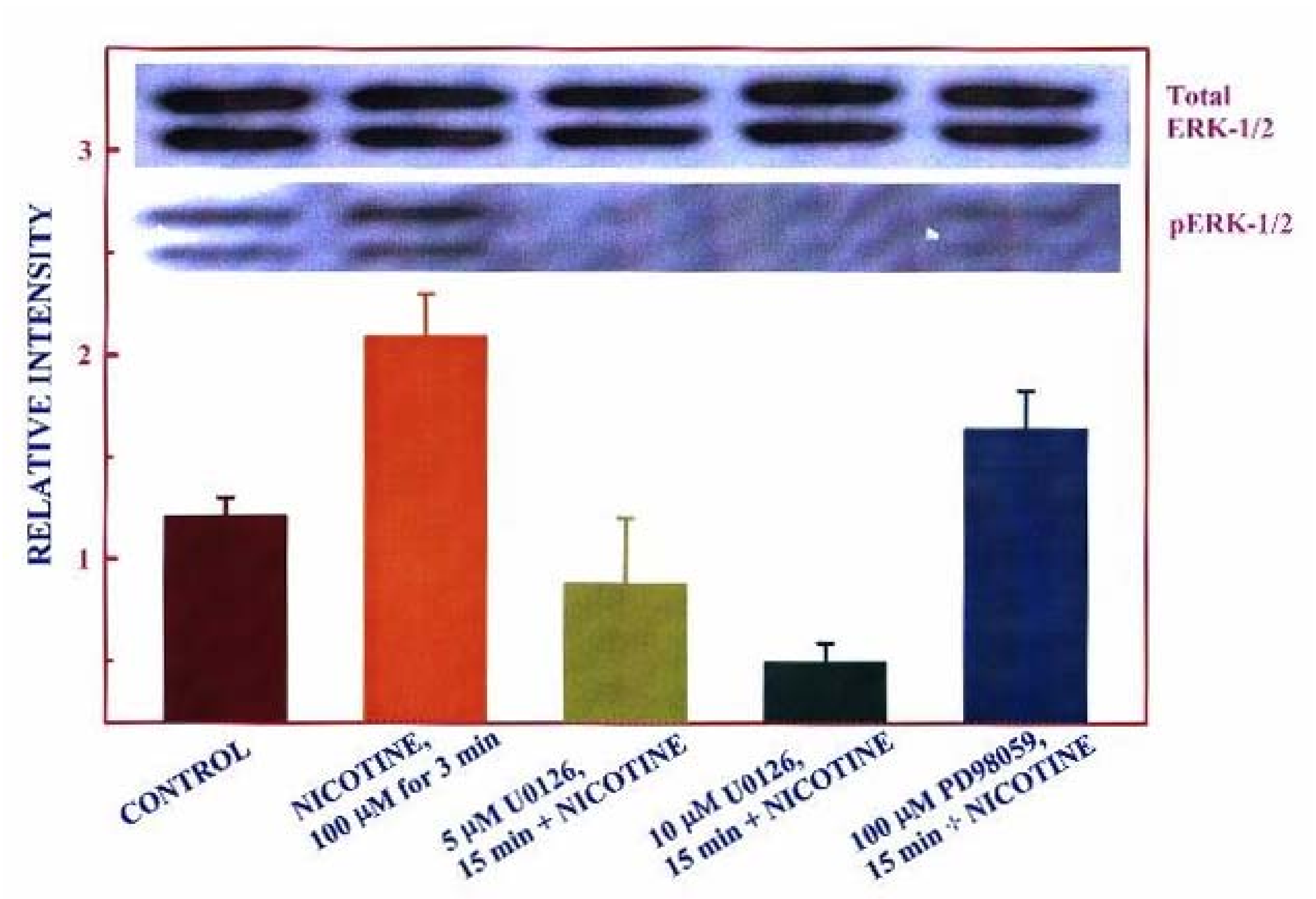

Nicotine Time And Dose Dependency Of Basal CCK- Stimulated Functional Response

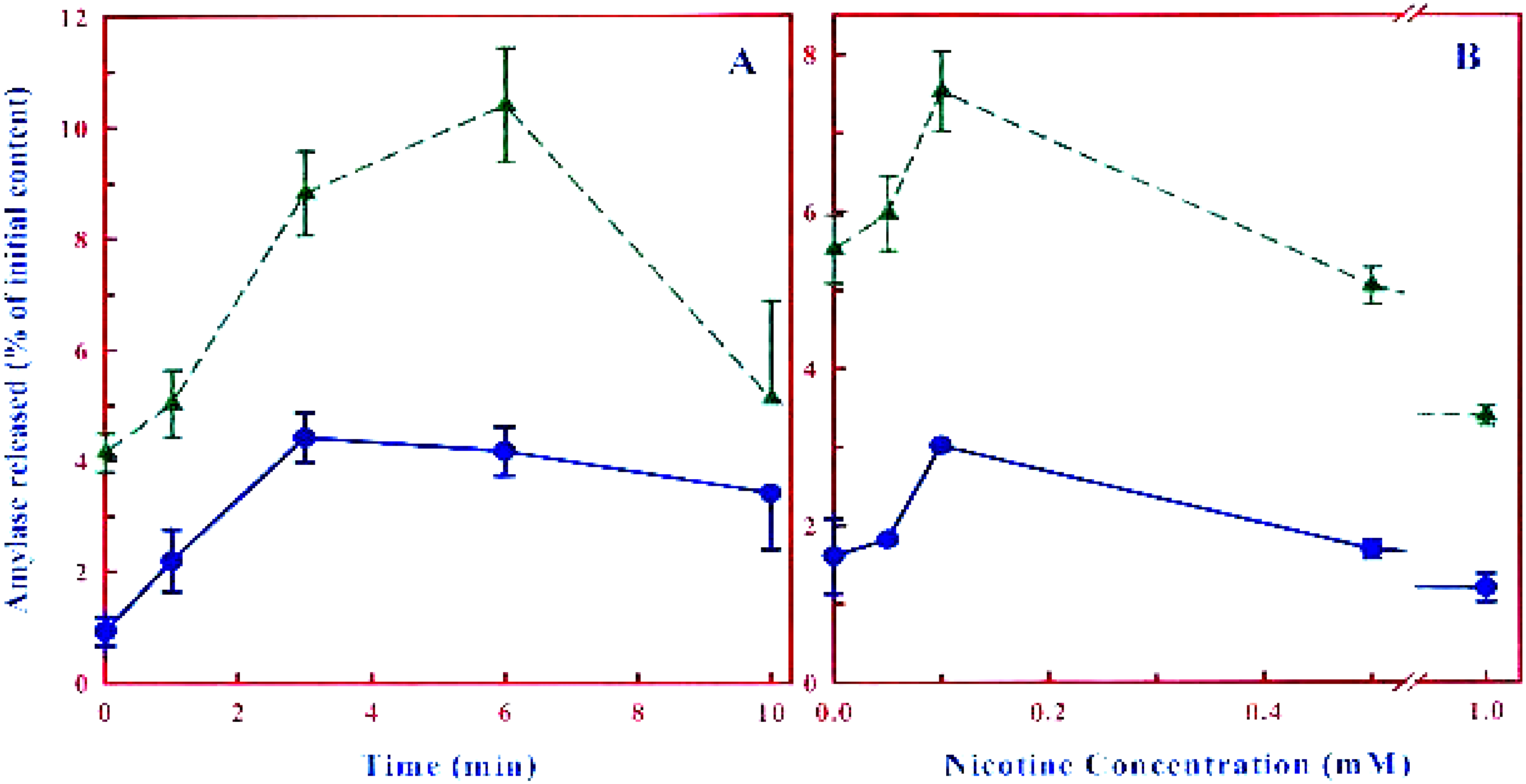

ERK Inhibitors Have No Effect On Nicotine Induced Functional Response In AR42j Cells

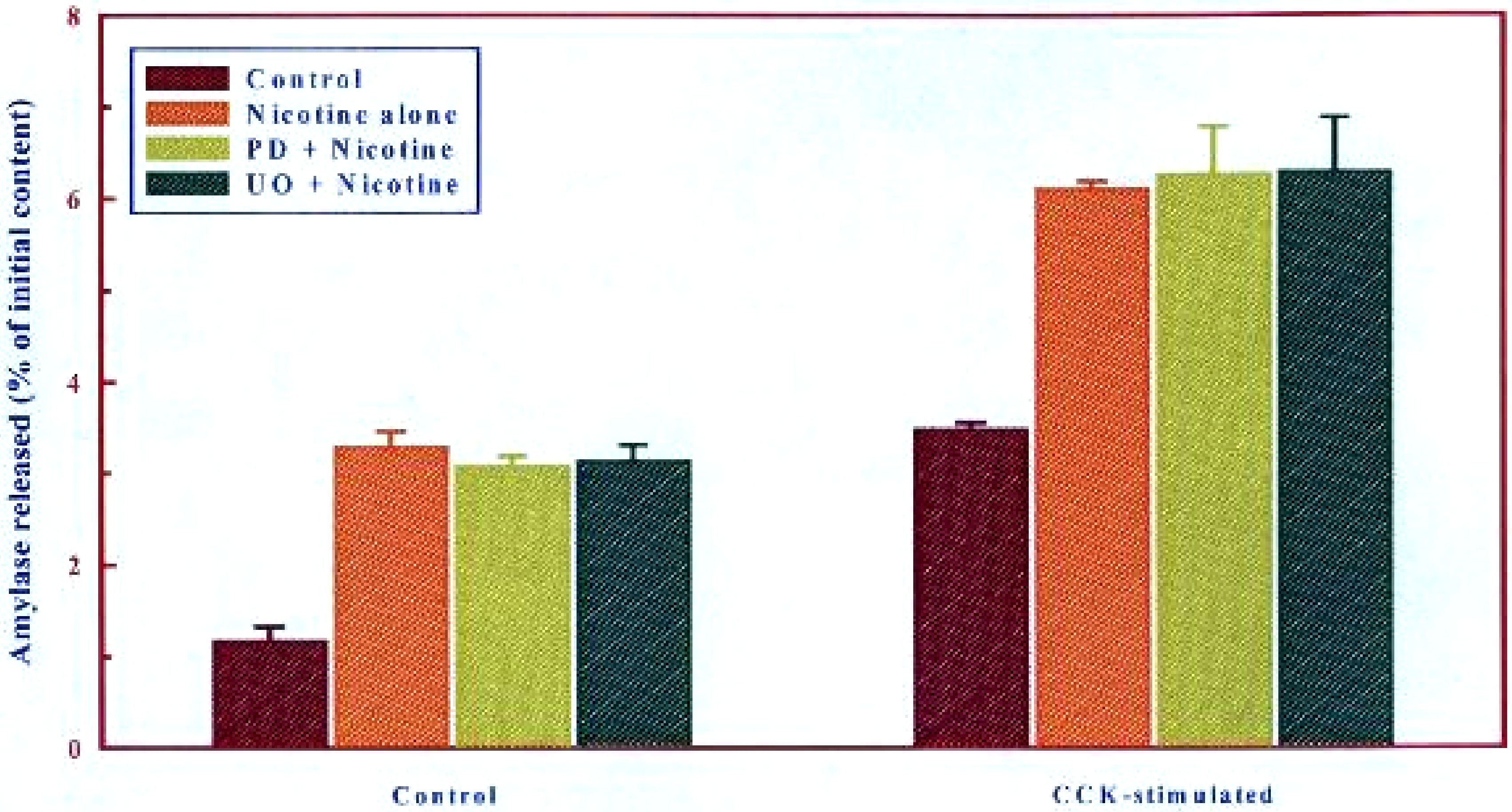

Influence Of A Nicotine Receptor Antagonist On Basal And CCK-stimulated Functional Response

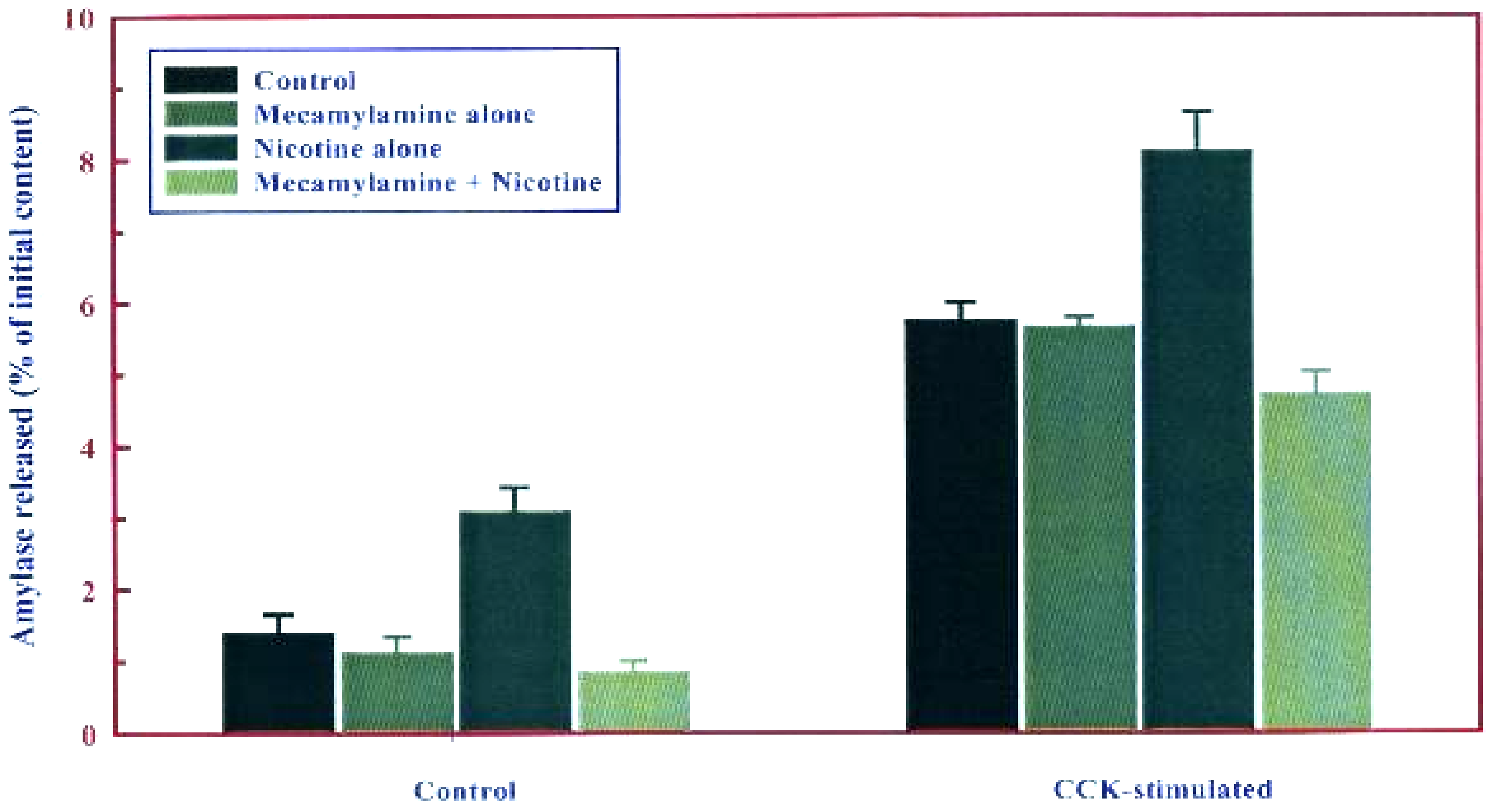

Time Dependent Changes In JNK And P38 Activation By Nicotine In AR42J Cells

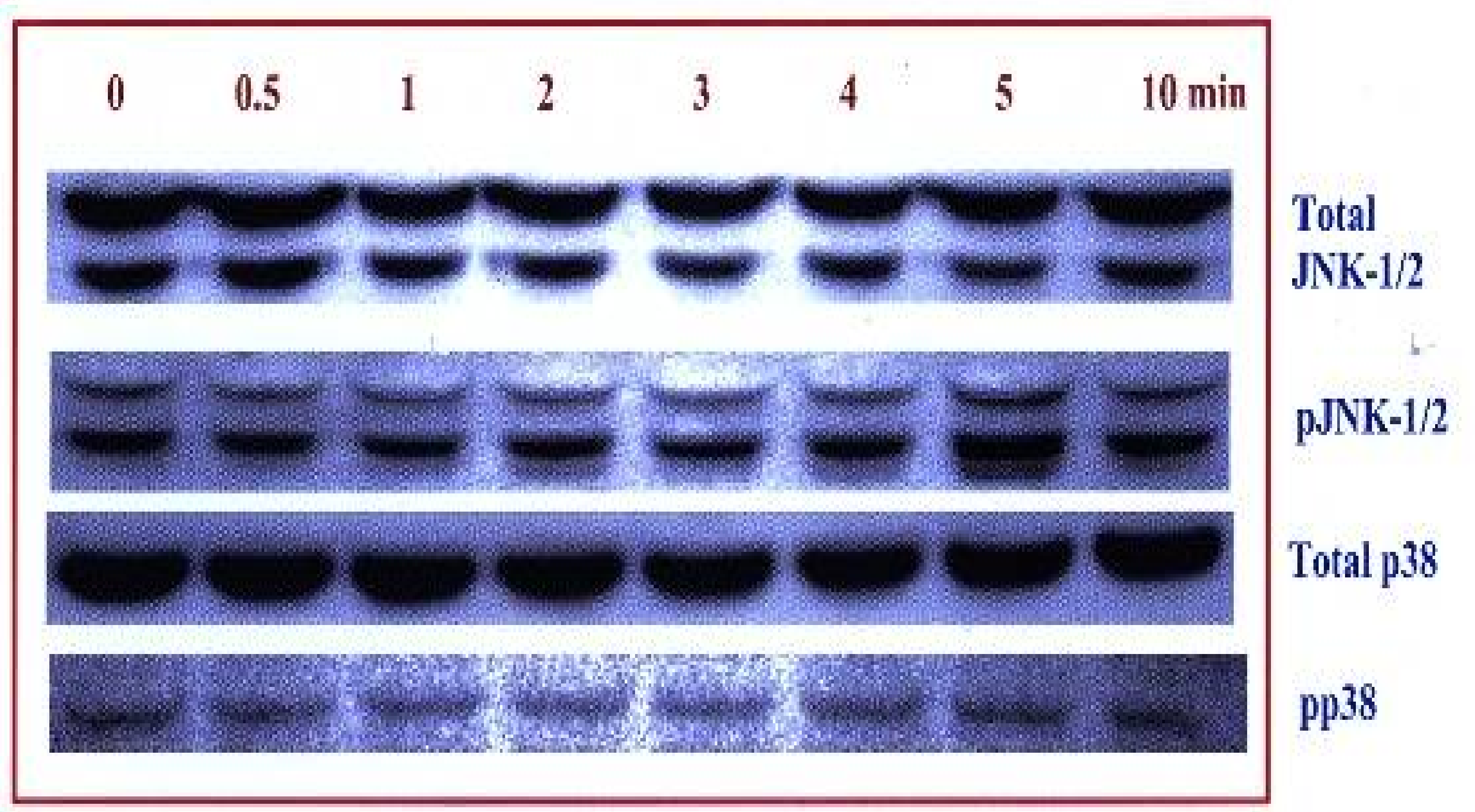

Photothermal Detection of Apoptosis induced by nicotine & Laser treatment in AR42J cells

Photothermal technique demonstrated capability to visualize Absorbing structures in different environments with PT microscope-spectrometer

The principle is to combine a dual-laser beam PT method with phase contrast technique

Changes in light absorbing structures by nicotine reflect in corresponding changes in laser-induced heat release and PT signal parameters

Control, no nicotine, 150 uJ pump energy

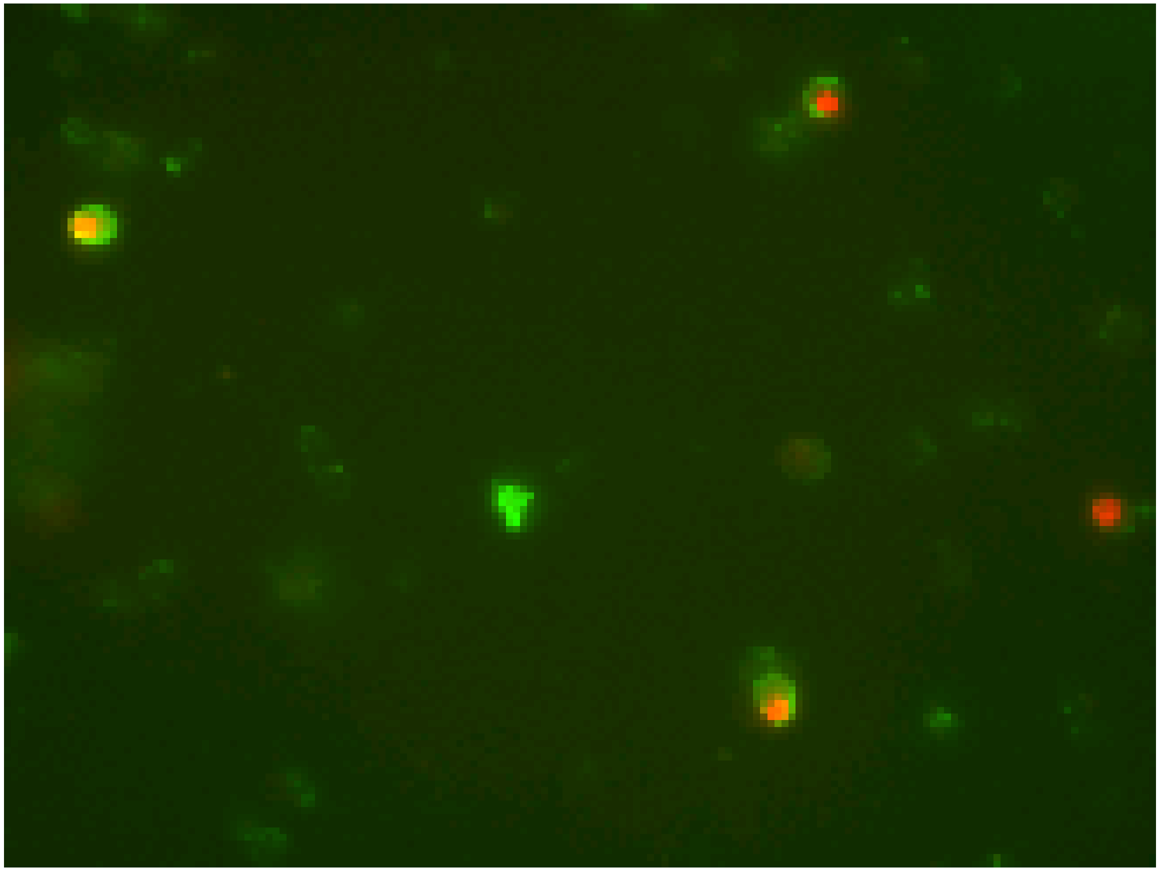

Nicotine, 10uM, 6 h + 150 uJ pump energy

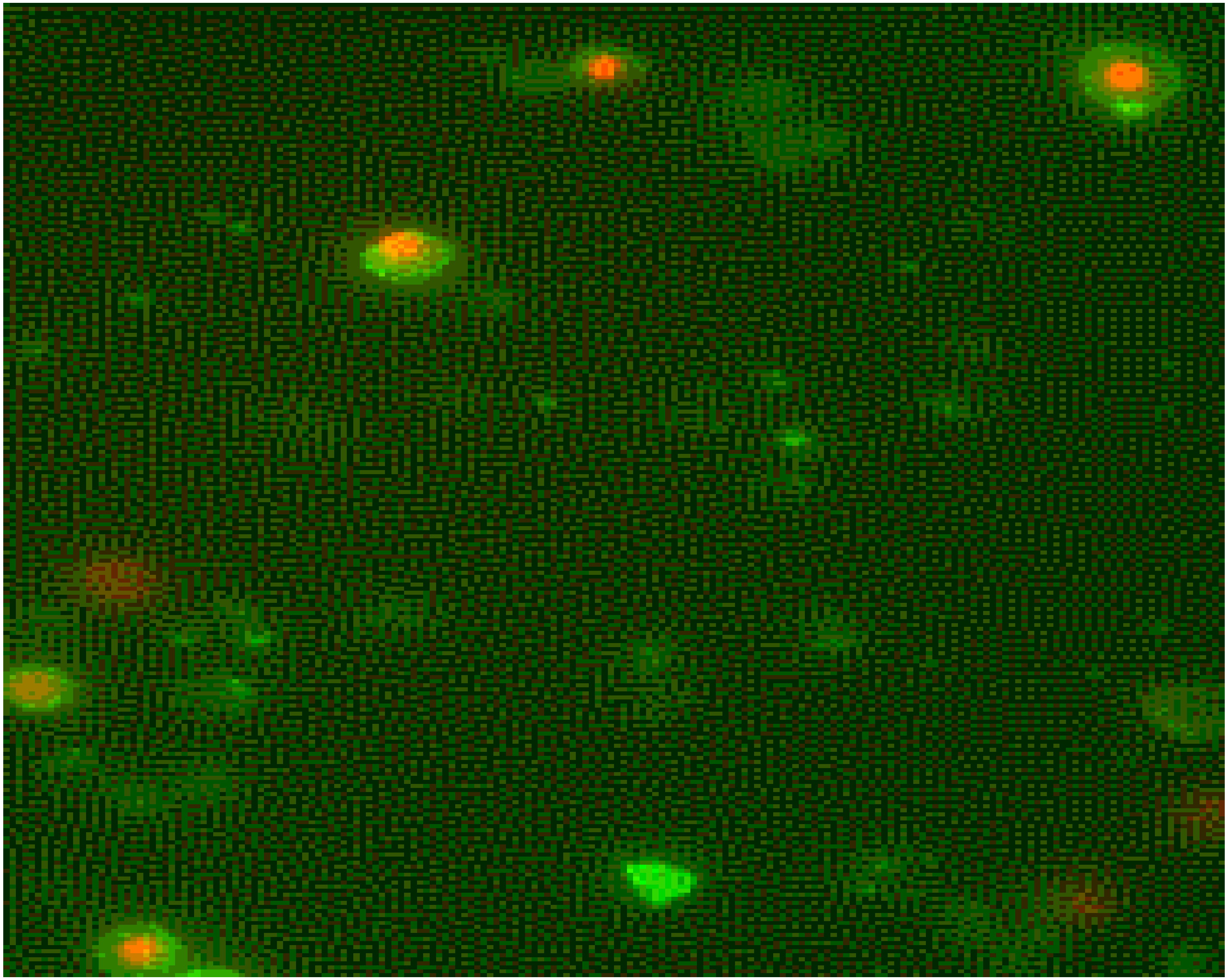

Nicotine 30 uM, 6h + 150 uJ pump energy

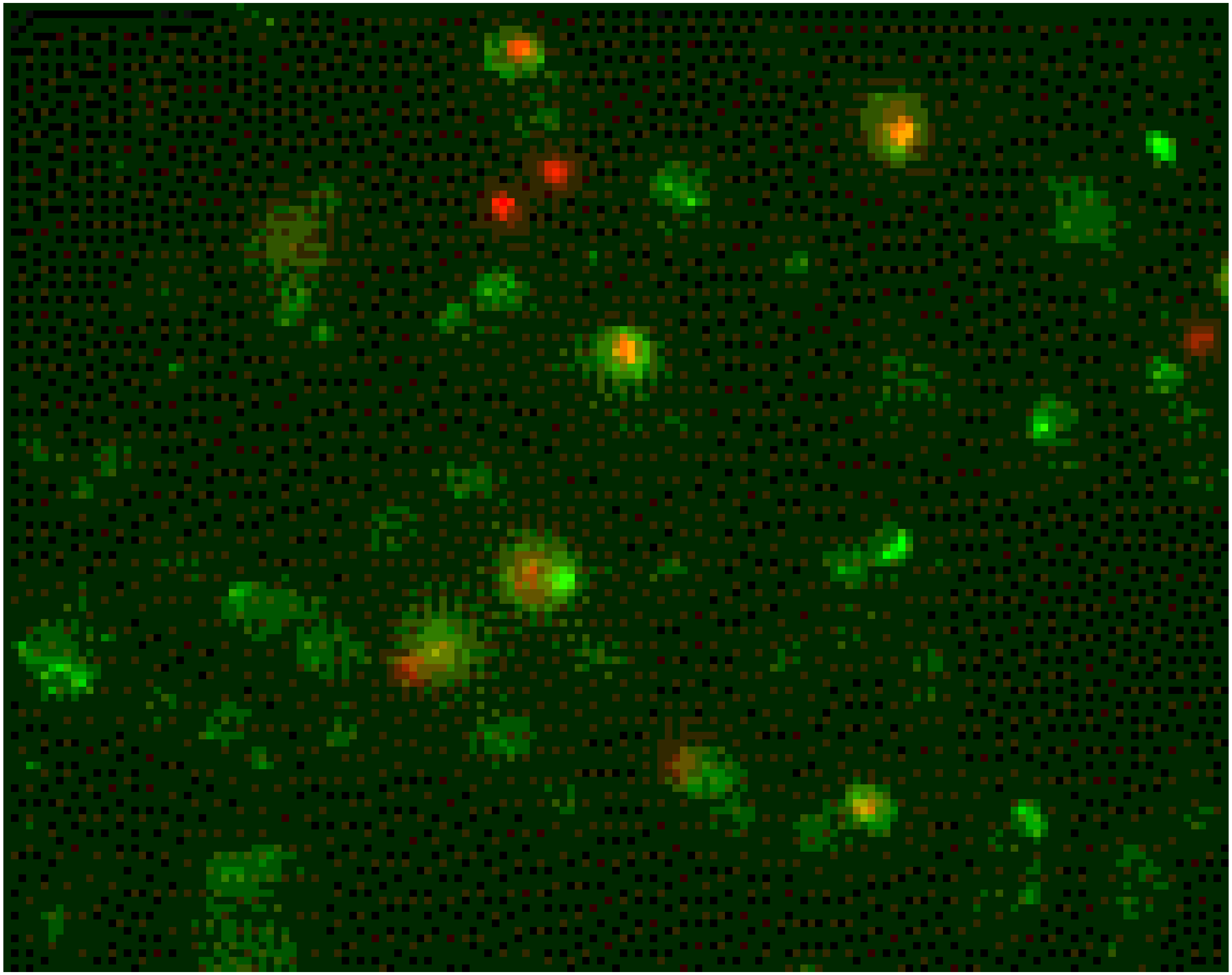

IMPLICATION

UNDERSTANDING OF PATHOLOGICAL AND BIOCHEMICAL MECHANISMS OF PANCREATIC CHANGES INDUCED BY NICOTINE WOULD ENABLE FUTURE DEVELOPMENT OF PREVENTIVE MEASURES

1) EDUCATION BASED PREVENTIVE APPROACH
2) NICOTINE GUM TREATMENT
3) DEVELOPMENT OF BLOCKING OR NEUTRALIZING AGENTS

Summary & Conclusions

Nicotine induced effects on the pancreas as demonstrated in in-vivo and in cell culture models appear consistent with the early pancreatic injury encountered in human pancreatitis

Further studies need to be conducted on the proto-oncogene expression and activation during the initial phase of the injury leading to pancreatic carcinogenesis

Besides nicotine, the role of other components of cigarette smoke need to be studied both in intact and cell culture models

Intervention approach will only be successful once the mechanism by which nicotine and other components of cigarette smoke induced pancreatic injury is known
