# Supplementary material for: Meeting materials from the 3rd Annual Meeting of the International Society for the Prevention of Tobacco Induced Diseases
Source: Tob Induc Dis. 2004 Dec 15;2(4):168. doi: 10.1186/1617-9625-2-4-168 (PMC2671527; doi:10.1186/1617-9625-2-4-168)
Supplement: Additional file 1 [file 1617-9625-2-4-168-S1.zip › Abstract 12-Smoking and pancreatic cancer.pdf]

Abstract 12

Sunday 09.00

Smoking and pancreatic cancer: Animal models of pancreatic dysfunction

Parimal Chowdhury

There is strong evidence in the literature linking cigarette smoking with pancreatic diseases. Smoking related deaths in the United States exceed 400,000 per year. Report of the Surgeon General published in 2004 showed that an increased risk for pancreatic cancer in smokers occurs as compared to lifetime non-smokers. We believe that the development of pancreatic cancer in smokers predisposes to the induction of pancreatitis like episode and nicotine, an addictive component in the cigarette smoke, enhances this process. In an effort to understand the mechanism by which nicotine induces this process we have conducted experiments in both in-vivo animal and in in-vitro cell culture models. For in-vivo experimentation we have exposed the Male Adult Sprague Dawley rats to nicotine by various routes. For cell culture models, we have used either primary cells from the animals, or used a stable rat pancreatic tumor acinar cell line, AR42J, and then exposed the cells to various concentrations of nicotine. The results from these experiments will be discussed. Based on our data from these experiments we conclude that the effects of nicotine on the pancreas could be stimulatory at low doses but at higher doses, representing increased number of smoked cigarettes, nicotine induces compromised pancreatic function that ultimately lead to oncogene expression and possible carcinogenesis. More detailed experimentation will be needed to confirm the current results.
