# Supplementary material for: Meeting materials from the 3rd Annual Meeting of the International Society for the Prevention of Tobacco Induced Diseases
Source: Tob Induc Dis. 2004 Dec 15;2(4):168. doi: 10.1186/1617-9625-2-4-168 (PMC2671527; doi:10.1186/1617-9625-2-4-168)
Supplement: Additional file 1 [file 1617-9625-2-4-168-S1.zip › Abstract 13-Systemic host responses in pregnant women.pdf]

Abstract 13

Sunday 09.30

Systemic host responses in pregnant women: Impact of smoking and periodontitis

J.L. Ebersole, M. J. Steffen, J. S. Hodges, and the OPT Study Group.
Univ. of Kentucky, Univ. of Minnesota, Univ. of Mississippi, and Columbia Univ.

Evidence is widely available linking smoking to numerous health problems affecting virtually all organ systems of the body. Recent data have even provided a calculation linking years of smoking to quantitative decrease in life-span due to the various negative health sequelae of smoking. Extensive health lobbying has occurred to encourage pregnant women to stop smoking due to the documented maternal and fetal adverse health affects. Moreover, smoking has been identified as a critical factor in contributing to periodontal disease, as well as response to therapy. The Obstetrics & Periodontal Therapy (OPT) project is a multi-center randomized clinical trial to evaluate the effect of periodontal therapy on the incidence of pre-term/low birthweight infants (PTB/LBW). This report describes some initial baseline findings on patients participating in the OPT study from 4 sites. Specifically, we report results on systemic host response biomarkers in 197 pregnant women with periodontitis. This population includes approximately 12.7% women who smoked or continue to smoke during their pregnancy. We evaluated a range of biomarkers in serum, many of which have been linked to PTB/LBW, that was collected at the time of entry into the study, including: PGE_2, MMP-9, CRP, fibrinogen, IL-1ß, IL-6, IL-8, TNFα, and IgG antibodies to 7 oral bacteria (*A. actinomycetemcomitans, P. gingivalis, P. intermedia, F. nucleatum, T. denticola, T. forsythia, C. rectus*). The results will describe the characteristic systemic response profiles in this cohort. Supported by DE014338 from the National Institute of Dental and Craniofacial Research.
