# Supplementary material for: Meeting materials from the 3rd Annual Meeting of the International Society for the Prevention of Tobacco Induced Diseases
Source: Tob Induc Dis. 2004 Dec 15;2(4):168. doi: 10.1186/1617-9625-2-4-168 (PMC2671527; doi:10.1186/1617-9625-2-4-168)
Supplement: Additional file 1 [file 1617-9625-2-4-168-S1.zip › Abstract 14-Smoking and wound healing.pdf]

## Abstract 14

## Sunday 10.30

### Smoking and wound healing

Ozan Akça, MD
Outcomes Research Institute, Department of Anesthesiology and Perioperative Medicine
University of Louisville, Kentucky, USA

Smoking has deleterious effects on the fundamental systems of organism. Its various ingredients including nicotine, carbon monoxide, and tar as well as the direct effects of these ingredients on endogenous triggers such as serotonin and inflammatory triggers initiate serious compromise in the recovery of tissue after surgical trauma. Suppression of fibroblasts and compromise in collagen formation directly interfere with wound healing. A few possible mechanisms may be responsible from the key physiopathology. Ischemia is the first one of these mechanisms. It was repeatedly shown that smoking decreases tissue oxygenation mostly due to nicotine derived vasoconstriction. Secondly, smoking interferes with basic immune response, and it initiates inflammatory cascade. Finally, smoking compromises hydroxyprolene deposition and suppresses fibroblasts, which means it directly, interferes with the collagen formation.

In this brief review, the evidence-based effects of smoking on wound healing and infections are discussed. Current evidence includes the harmful effects of smoking on bone formation after long bone and spine surgery; wound dehiscence in plastic surgical procedures including abdominoplasties and breast reconstructions, and wound healing and infection problems in colon surgery. There is considerable evidence on harmful effects of smoking on wound healing. Additionally, there are conflicting evidences on the effect of smoking on surgical wound infections. In summary, smoking compromises tissues to recover after surgical interventions, and most likely explanation of the mechanism is hypoxia-triggered cascades.
