# Supplementary material for: Meeting materials from the 3rd Annual Meeting of the International Society for the Prevention of Tobacco Induced Diseases
Source: Tob Induc Dis. 2004 Dec 15;2(4):168. doi: 10.1186/1617-9625-2-4-168 (PMC2671527; doi:10.1186/1617-9625-2-4-168)
Supplement: Additional file 1 [file 1617-9625-2-4-168-S1.zip › Abstract 15-Sudden infant death syndrome.pdf]

Abstract 15

Sunday 11.00

Sudden infant death syndrome

Karen A. Waters, MBBS, PhD
Department of Pediatrics (Sleep Medicine)
University of Louisville, Kentucky, USA

Exposure to cigarette smoke, either in utero or after birth, is a known risk factor for a variety of diseases during infancy and childhood. This talk will focus on cigarette smoke exposure as a risk factor for sudden infant death syndrome, and the mechanisms by which it has been shown to affect the development of pathways which control breathing. A background of recent developments in the epidemiology of SIDS and its known risk factors, will provide background for why there is a current focus on cigarette smoke exposure as a preventable, but major risk factor for this major cause of infant mortality.

Basic mechanisms which may be responsible for death in infants will then be discussed. These will focus on effects of early exposure to nicotine, and its effect on development of the autonomic nervous system, and sequelae of cellular exposure to hypoxia. Because nicotine has direct neurotransmitter effects on the nicotinic receptors of the autonomic nervous system, many studies have examined the effects of nicotine exposure alone. However, hypoxic-hypoxia which can be induced by carbon monoxide, is another mechanism of relevance which has been studied. Studies in my laboratory focus on the interactions between hypoxia (with or without hypercapnia) and its interactions with nicotine, and results from these studies will be presented.
