# Supplementary material for: Meeting materials from the 3rd Annual Meeting of the International Society for the Prevention of Tobacco Induced Diseases
Source: Tob Induc Dis. 2004 Dec 15;2(4):168. doi: 10.1186/1617-9625-2-4-168 (PMC2671527; doi:10.1186/1617-9625-2-4-168)
Supplement: Additional file 1 [file 1617-9625-2-4-168-S1.zip › Abstract 16-Birth defects and their association with tobacco smoke.pdf]

Abstract 16

Sunday 11.30

Birth defects and their association with tobacco smoke

D.L. Molfese, V.J. Molfese, M. Ferguson, A.P.F. Key, S. Straub, K. Peach & N. Pratt.
Psychological & Brain Sciences, University of Louisville, Kentucky, USA

Auditory event-related brain potentials (ERPs) were recorded from 128 electrodes placed on the scalp of two groups of infants, one of whom were born to mothers who smoked approximately one pack of cigarettes per day during the pregnancy and a control group born to non-smoking mothers. The two groups were matched for sex, birth weight, gestational age, apgar scores, mother's age, gravidity, mothers education level, and annual income. ERPs to speech differed between the two groups. The Control group exhibited a standard left hemisphere advantage for processing the speech sounds while the Smoking exposed group exhibited no brain lateralization for speech. In addition, processing of speech occurred over a few brain regions while infants exposed prenatally to smoke required more brain areas to process the same information. These findings suggest marked changes in brain organization from birth in infants prenatally exposed to cigarette smoke. Moreover, the brains of these infants appeared to process the speech in atypical ways that required more brain involvement, suggesting less efficient brain processing resulting from the smoking history.
