# Supplementary material for: Meeting materials from the 3rd Annual Meeting of the International Society for the Prevention of Tobacco Induced Diseases
Source: Tob Induc Dis. 2004 Dec 15;2(4):168. doi: 10.1186/1617-9625-2-4-168 (PMC2671527; doi:10.1186/1617-9625-2-4-168)
Supplement: Additional file 1 [file 1617-9625-2-4-168-S1.zip › Abstract 17-Treating tobacco dependence.pdf]

Abstract 17

Monday 08.40

Treating tobacco dependence

Richard D. Hurt, MD, Professor of Medicine
Director, Mayo Clinic Nicotine Dependence Center, Rochester, Minnesota, USA

State of the art treatment of tobacco dependence involves four components: behavioral modification, addictions treatment, pharmacotherapy, and relapse prevention. Behavioral treatment can be provided by a wide range of health care providers, individual counseling or group programs. The USPHS Guideline of 2000 calls for intra and extra treatment support in addition to motivational interviewing as a technique for behavioral counseling. Certainly, providing such services in a healthcare setting capitalizes on “teachable moments”. It is also important for patients and clinicians alike to understand that tobacco dependence has all the hallmarks of an addictive disorder. There are now six approved pharmacotherapies for treating tobacco dependence, and the USPHS Guideline recommends using at least one for every patient who is trying to stop smoking. Approved medications include nicotine gum, nicotine lozenge, nicotine patches, nicotine nasal spray, nicotine inhaler, and bupropion. Clinical decision making about pharmacotherapy is based upon clinicians using their clinical skills and knowledge of pharmacotherapy to individualize each patient’s drug dose. From a practical standpoint, nicotine patch therapy or bupropion are viewed as a floor on which to build pharmacotherapy. Either can be used alone in treating patients with mild-moderate tobacco dependence. Depending upon the patient, nicotine patch therapy may be used in combination with bupropion, while shorter acting NRT products are used to control intermittent withdrawal symptoms. Much of the clinical decision making is based on the patient’s past experience and the patient’s preference. Relapse prevention strategies include: face-to-face follow-up and ongoing support, support groups, telephone counseling, or prolonged pharmacotherapy.
