# Supplementary material for: Meeting materials from the 3rd Annual Meeting of the International Society for the Prevention of Tobacco Induced Diseases
Source: Tob Induc Dis. 2004 Dec 15;2(4):168. doi: 10.1186/1617-9625-2-4-168 (PMC2671527; doi:10.1186/1617-9625-2-4-168)
Supplement: Additional file 1 [file 1617-9625-2-4-168-S1.zip › Abstract 18-Tobacco cessation - Rates of success.pdf]

Abstract 18

Monday 10.45

Tobacco cessation – Rates of success

Kazunari Satomura, MD, PhD
Dept. of Public Health, Faculty of Medicine, Kyoto University

However smoking is one of the biggest harm for health of not only smokers but also non-smokers around smoking, smoking rates of male are over 30% and those of female are over 10% in the world. To reduce smoking rates several kinds of smoking cessation programs were performed. Their success rates were about 30% in almost all programs. The reasons of low success rates are nicotine dependency and types of smoking. By metaanalysys of results showed some useful points of increasing success rates. For example, advice by physical clinician are useful, nicotine replacement therapies are useful and so forth. To increase success rates, combinations of several kinds of smoking cessation methods according to individual dependency level of nicotine and types of smoking should be developed.
