# Supplementary material for: Meeting materials from the 3rd Annual Meeting of the International Society for the Prevention of Tobacco Induced Diseases
Source: Tob Induc Dis. 2004 Dec 15;2(4):168. doi: 10.1186/1617-9625-2-4-168 (PMC2671527; doi:10.1186/1617-9625-2-4-168)
Supplement: Additional file 1 [file 1617-9625-2-4-168-S1.zip › Abstract 19-Smoking cessation following cancer diagnosis.pdf]

Abstract 19

Monday 10.10

Smoking cessation following cancer diagnosis

Jamie L. Studts, PhD
James Graham Brown Cancer Center and University of Louisville School of Medicine
Louisville, Kentucky, USA

While most individuals accept that smoking increases risk for several types of cancer, the benefits of smoking cessation following a cancer diagnosis are less commonly discussed. Among individuals newly diagnosed with smoking-linked malignancies of the lung, head, and neck, there are numerous incentives to quit smoking. Incentives include: (1) reduced risk of second primary malignancies, (2) improved quality of life, (3) fewer treatment-related symptoms, and (4) longer overall survival. Incentives to quit smoking exist for other malignancy groups as well, including improved wound healing following surgery, as well as cardiovascular, pulmonary, and overall physical functioning benefits. However, surprisingly high rates of cancer survivors continue to smoke throughout treatment or return to smoking at the end of treatment. Cancer survivors who smoke following diagnosis and treatment are often the individuals who are most likely to receive the greatest benefit of cessation – patients diagnosed with limited disease. Several authors have suggested that a cancer diagnosis should be considered a “teachable moment” for smoking cessation. Certain strategies have been proven to be effective in the general population of smokers and are likely to be effective in individuals diagnosed with cancer. However, the additional sense of urgency and distress surrounding diagnosis and treatment frequently complicate cessation efforts, requiring more intensive interventions. Individuals diagnosed with cancer who continue to smoke may require a higher level of care that provides comprehensive interventions designed to address multiple aspects of nicotine dependence, including physical, psychological, social, and behavioral components.
