# Supplementary material for: Meeting materials from the 3rd Annual Meeting of the International Society for the Prevention of Tobacco Induced Diseases
Source: Tob Induc Dis. 2004 Dec 15;2(4):168. doi: 10.1186/1617-9625-2-4-168 (PMC2671527; doi:10.1186/1617-9625-2-4-168)
Supplement: Additional file 1 [file 1617-9625-2-4-168-S1.zip › Abstract 20-Youth smoking and youth smoking prevention (YSP) in Hong Kong.pdf]

Abstract 20

Monday 10.40

Youth smoking and youth smoking prevention (YSP) in Hong Kong

Lam TH, Ho SY, Lai MK
Department of Community Medicine, University of Hong Kong, Hong Kong, China

The Hong Kong Council on Smoking and Health (COSH) commissioned three Youth Smoking Surveys in Hong Kong in 1994, 1999 and 2003, to study smoking prevalence, factors associated with smoking, respiratory symptoms, quitting and to monitor smoking trends. The Global Youth Tobacco Survey has been incorporated in the 2003 survey.

The 1994 survey showed significant linear increase in respiratory symptoms with advancing smoking status (from weekly to more than 6 cigarettes daily) with adjusted odds ratios (OR) of 1.60 to 2.23 for throat problems, 1.56 to 3.02 for cough, 1.42 to 4.84 for phlegm and 1.39 to 3.48 for wheezing. Associations and significant linear trends were also found between respiratory symptoms and the number of smoking household members (an indicator of passive smoking) in never-smoking students. Risk factors for ever smoking included poor knowledge about health hazards of smoking (adjusted OR: 1.91, 95% CI: 1.56-2.31), positive attitudes toward smoking (2.08, 1.82-2.39), participation in cigarette promotion or sponsored activities (1.24, 1.08-1.42) and perceiving cigarette advertisements as attractive (2.68, 2.33-3.07).

The 1999 survey found widespread overestimation of peer smoking prevalence in students regardless of sex and smoking status, and over half of all students grossly overestimated (over two times) the actual prevalence of smoking. Such students had excess risks of 162% (95% CI: 92-259%) for current smoking, 66% (40-97%) for ever smoking, 82% (36-143%) for intention to smoke when offered, 91% (39-162%) for intention to smoke within the next year, and 87% (34-160%) for intention to smoke when grown up. The proportion of current smoking students who had ever wanted to quit smoking increased from 48.7% in 1994 to 67.1% in 1999.

The prevalence of current smoking in Form 1, 2 and 3 students had increased from 6%, 8% and 10% in 1994 to 9%, 12% and 13% in 1999, respectively. Such increases were observed in both boys and girls but the doubling from 6% to 12% in Form 3 girls was most striking.

Data analysis of the third Youth Smoking Survey is on-going.

In Hong Kong in 2001, the tobacco industry gave HK$18 million (US$2.31 million) to the Youth Smoking Prevention (YSP) programme. With two years, the YSP has successfully created much confusion and distraction, and has become a major obstacle for tobacco control in Hong Kong. Globally, at least 130 YSP programmes have been funded by the tobacco industry. The WHO has published an anti-YSP booklet and the Hong Kong COSH has published a Chinese version and held a press conference to start fighting back against the YSP.
