# Supplementary material for: Meeting materials from the 3rd Annual Meeting of the International Society for the Prevention of Tobacco Induced Diseases
Source: Tob Induc Dis. 2004 Dec 15;2(4):168. doi: 10.1186/1617-9625-2-4-168 (PMC2671527; doi:10.1186/1617-9625-2-4-168)
Supplement: Additional file 1 [file 1617-9625-2-4-168-S1.zip › Abstract 21-Predictors of intention to quit and successful quitting among the Chinese.pdf]

Abstract 21

Predictors of intention to quit and successful quitting among the Chinese elderly smokers

Abu Saleh M. Abdullah*[1], Lai-Ming Ho[1], Yam H Kwan[1], Cheung WL[2], McGhee SM[1].
[1]Department of Community Medicine, The University of Hong Kong, Hong Kong SAR, China.
[2]Caritas Medical Centre, Hong Kong SAR, China.

Purpose. To assess the predictors of smoking cessation and intention to quit among the Chinese elderly smokers.
Design. A cross sectional survey of elderly smokers.
Setting. Shamsuipo district, Hong Kong.
Subject. A total of 1318 elderly were interviewed (response rate = 83%).
Measures. A structured questionnaire was used for data collection. The questionnaire sought information on the subject's socio-demographic background and smoking habits. The predictors for successful quitting and intention to quit were assessed by chi-square tests and multiple logistic regression.
Results. Of the respondents, 20.2% were current smokers, 25.4% were ex-smokers and 54.4% were never smokers. Of the smoker (current and ex-smokers) respondents, 55.7% (335/601) had successfully quit at the time of enumeration. The predictors of quitting were being attender of mobile clinic, living with others, receiving assistance for mobility, being non-drinkers, smoking for 30 years or less and smoking more than 10 cigarettes per day. Being aged between 60 and 70, smoking for 30 years or less, smoking 10 cigarettes per day or less and having health problems in the past were predictors of intention to quit smoking.
Conclusion. The study identified several predictors of successful quitting which could help improve the provision of current smoking cessation services. Population-based smoking cessation programs, especially those targetted to elderly, should take these predictors into consideration in the design of interventions.
