# Supplementary material for: Meeting materials from the 3rd Annual Meeting of the International Society for the Prevention of Tobacco Induced Diseases
Source: Tob Induc Dis. 2004 Dec 15;2(4):168. doi: 10.1186/1617-9625-2-4-168 (PMC2671527; doi:10.1186/1617-9625-2-4-168)
Supplement: Additional file 1 [file 1617-9625-2-4-168-S1.zip › Abstract 22-Predictors of household smoking policies among San Diego residents of.pdf]

Abstract 22

Predictors of household smoking policies among San Diego residents of Mexican descent

Marc A. Adams MPH, Ana P. Martinez-Donate PhD, Melbourne F. Hovell PhD, C. Richard Hofstetter PhD, Carlos Vera, Gloria Valerio
Center for Behavioral Epidemiology and Community Health Graduate School of Public Health, San Diego State University.

Residential smoking bans are associated with decreased levels of smoking and environmental tobacco smoke (ETS) exposure. The present study explored correlates of home smoking policies among Mexican-descent residents in San Diego, CA, the largest regional minority group. Preliminary data (N=813) were analyzed from an ongoing population survey on tobacco use and policies. Using a cross-sectional design, participants were contacted through random digit dialing and interviewed either in Spanish or English on household policies, tobacco use, and attitudes regarding ETS exposure. Based on the Behavioral Ecological Model, the interview included measures on ecological factors, such as characteristics of household residents, social contingencies regarding tobacco use, ETS exposure, and acculturation. About 91.8% of participants reported a complete smoking ban in their home. A logistic regression model indicated that, after adjusting for demographics, participants living with a smoker (OR=.28, 95% CI, .14-.53) were less likely to report a complete smoking ban. A complete ban was associated with having smoked <100 cigarettes during their lifetime (OR=2.8, 95% CI, 1.2-6.6) and holding negative attitudes towards ETS exposure (OR=3.4, 95% CI, 1.5-7.5). At the inter-personal level, spouse disapproval of smoking (OR=1.5, 95% CI, 1.02-2.3), family and friends' support for ETS policies (OR=1.8, 95% CI, 1.4-2.4), and their intolerance towards smoking (OR=1.4, 95% CI, 1.1-1.9) were positively associated with a home ban. The results suggest the importance of the social environment, in addition to intrapersonal factors, on the adoption of smoke free residential policies among Mexican-Americans, and have important implications for the design of risk reduction interventions.
