# Supplementary material for: Meeting materials from the 3rd Annual Meeting of the International Society for the Prevention of Tobacco Induced Diseases
Source: Tob Induc Dis. 2004 Dec 15;2(4):168. doi: 10.1186/1617-9625-2-4-168 (PMC2671527; doi:10.1186/1617-9625-2-4-168)
Supplement: Additional file 1 [file 1617-9625-2-4-168-S1.zip › Abstract 23-The effects of cigarette smoke on hepatic and pulmonary xenobiotic metabolizing.pdf]

Abstract 23

The effects of cigarette smoke on hepatic and pulmonary xenobiotic metabolizing enzymes in rats

B.C. Eke , M. __can*
Department of Toxicology, Faculty of Pharmacy, Ankara University,
06100 Tando_an- Ankara, TURKEY

The effects of cigarette smoke (CS) on hepatic and pulmonary monooxygenase (MO) activities (aniline 4- hydroxylase, AH; aminopyrine N-demethylase, AMND; 7-ethoxyresorufin O- deethylase, EROD; p- nitroanisole O- demethylase, p-NAOD), lipid peroxidation (LP) and reduced glutathione (GSH) levels and glutathione S-transferases (GST)s activities toward several substrates (1-chloro-2,4-dinitrobenzene, CDNB; 1,2-dichloro-4-nitrobenzene, DCNB; ethacrynic acid, EAA; 1,2-epoxy-3-(p-nitrophenoxy)-propane, ENPP) were determined in adult male rats. The male rats were exposed to CS 5 times a day, with 1 h intervals, for 3, 7 and 15 days in a chamber where smoke and fresh air lead alternatively and were killed 16 hours after the last treatments. The maximum alterations were observed in the activities of MOs and GSTs after exposing the animals to CS 5 times a day for 3 days in a chamber alternatively supplied with smoke and fresh air. Therefore, this regimen of treatment has been chosen to find out the pattern of influence of smoke from cigarettes with different tar contents on hepatic and pulmonary activation and detoxication enzymes in male rats.

CS of low and high tar content cigarettes significantly increased hepatic and pulmonary EROD and p-NAOD activities as compared to controls. Hepatic AMND and pulmonary AH activity significantly increased with CS of high tar content cigarettes whereas did not change with that of low content cigarettes. LP levels significantly decreased and increased with CS of high tar content cigarettes, respectively, but remained unaltered with that of low content cigarettes in liver and lung. GSH levels significantly increased with CS of low and high tar content cigarettes in lung. In liver, GSH levels significantly increased with CS of only high tar content cigarettes. Hepatic GST activities toward EAA significantly increased with CS of high tar content cigarettes whereas decreased with that of low tar content cigarettes. ENPP GST activity decreased with CS of low and high tar content cigarettes in liver. In lung, GST activity toward CDNB, DCNB, EAA and ENPP significantly decreased with CS of high tar content cigarettes whereas only GST activity toward DCNB significantly decreased with CS of low tar content cigarettes. All other GST activities toward CDNB, DCNB, EAA and ENPP did not change in liver and lung, respectively.

These studies reveal that the regulations of the hepatic and pulmonary MO and GST are differentially influenced by CS as a function of time and tar content.
