# Supplementary material for: Meeting materials from the 3rd Annual Meeting of the International Society for the Prevention of Tobacco Induced Diseases
Source: Tob Induc Dis. 2004 Dec 15;2(4):168. doi: 10.1186/1617-9625-2-4-168 (PMC2671527; doi:10.1186/1617-9625-2-4-168)
Supplement: Additional file 1 [file 1617-9625-2-4-168-S1.zip › Abstract 24-Epidemiology and prevention of tobacco use in Tunisia.pdf]

Abstract 24

Epidemiology and prevention of tobacco use in Tunisia

Radhouane FAKHFAKH*, Mohamed HSAIRI, Noureddine ACHOUR
Institut Naitonal de Sante Publique, 5-7 rue Khartoum IMM Diplomat 1002 Le belvedere, Tunis

Introduction: Our work describes trends in tobacco sales and smoking prevalence in the Tunisian population, it estimates the consequences of smoking on mortality of this population, and discusses anti-tobacco actions: Educational actions, legislative measures, and price increases.

Data sources: Sales Data were collected from the Tunisia tobacco monopoly, smoking prevalence data from surveys conducted by several institutes, and, Numbers of deaths by causes have been estimated from WHO for 1998 year.

Results: Tobacco sales increased from 4,96g per adult per day in 1981 to 6,3g in 1993, then decreased widely. The proportion of smokers was 30% in 1996, 55% among men versus 5,6% among women. Among 12 to 20 years Tunisian schooled teenagers, 36,3% smoked at least one cigarette in their life. Boys try cigarettes more frequently than girls (55.8% vs 17,7%). Besides 12,1% of teenagers smoked tobacco with a higher prevalence among boys than girls (21,3% vs 3,3%) and in a more regular way. Mortality attributable to tobacco in Tunisia has been estimated to 6430 deaths in 1997 (5580 among men versus 850 among women). These deaths represent 22% of total male deaths and 4% of female ones. Anti-tobacco measures have been reinforced by the enactment of anti-tobacco law.

Conclusion: Proportion of young smokers, remaining high, it is expected that the consequences of tobacco addiction in Tunisia in term of mortality will be even heavier in the next two decades if efficient anti-tobacco actions are not implemented.
